## [Editor Report]

This important work builds on the conceptual framework of host-pathogen interactions and co-evolution, adding new examples of co-divergence of primate herpesviruses with their respective host restriction factors. The authors convincingly outline the degree to which their initial findings (BORF2 and A3B interactions) are conserved across other herpesvirus RNRs, and place them in the context of the evolution of the A3 gene locus and expansion. This work will be of great interest to virologists. Especially those that work in the field of host pathogen evolution and the molecular arms race.

---

## [Decision Letter]

[Editors' note: this paper was reviewed by Review Commons.]

---

## [Author Response]

We would like to thank all three reviewers for their time and thorough assessment of our manuscript. We appreciate their constructive feedback and believe our work has been considerably strengthened by addressing the comments, suggestions, and concerns raised during peer review. In the following responses, we address the reviewer’s critiques point-by-point.

Reviewer 1Reviewer #1 (Evidence, reproducibility and clarity (Required)):Moraes et al. build upon their recent studies of APOBEC3 antagonism by EBV BORF2 by showing that additional RNR subunits encoded by other herpesviruses share this activity, suggesting that the host-virus arms races involving APOBEC3 proteins is more widespread than previously thought. Furthermore, the authors show that herpesviruses infecting primates that lack A3B (New World Monkeys) do not apparently exhibit a capacity to antagonize human A3B, suggesting that this function was not required during the evolution of those viruses (while it seemingly was important for viruses infecting hosts that encode A3B). Overall, this is a technically sound submission that combines confocal immunofluorescence, co-immunoprecipitation, and enzymatic assays to comprehensively test the sensitivity of different A3s to counteraction by viral RNRs. The enzymatic (deamination) assays performed prove to be the most insightful, since co-IP and colocalization microscopy was not entirely sufficient to reveal which domains of A3 are important for targeting by RNRs. It is well-written, well-organized, and well-referenced, and will be of interest to readers who study APOBEC3s, herpesviruses, and host-virus arms races more generally.

Thank you for appreciating the technical aspects and broader impact of our studies.

Major points:1. Figure 4B: the fluorescence microscopy data does not match well with the Co-IP (in Figure 4A). For example, L1B in A3A enhances the A3A-BORF2 co-IP but no clear differences are observed in colocalization. Or are the authors claiming the presence of L1B results in greater colocalization between A3A and BORF2, because there is slightly less diffuse BORF2 in the cytoplasm under these conditions? If that is the case, then quantitative colocalization analysis will need to be performed. In general, virtually none of the colocalization analysis in Figure 4B matches well with the co-IP results of Figure 4A. The authors take this to suggest that L7, and not L1, is most important determinant for BORF2 binding to A3s, but in that case, then the colocalization data is disconnected functionally from co-IP results. This is not necessarily a large problem, since the authors ultimately test the enzymatic activity of A3s in the presence of different RNRs. These latter functional experiments more objectively define what regions of A3s are important for antagonism by RNRs.

Thank you for giving us an opportunity to clarify these important points. We understand how the fluorescence microscopy data in Figure 4B may at first appear to disagree with the co-IP results in Figure 4A. However, we would like to point out that WT A3A—which has a shorter L1 region and binds less strongly to BORF2 compared to A3B—is nevertheless efficiently relocalized by BORF2 (PMID 35476445, 31534038, 31493648). We believe that this observation can be explained by compensatory avidity interactions during cytoplasmic aggregate formation in living cells, a process mediated by the formation of a non-canonical BORF2-BORF2 dimer as detailed in our recent cryo-EM studies (PMID 35476445). These avidity interactions explain how a weaker interaction (as indicated by weaker co-IP levels) can still result in the formation of large cytoplasmic aggregates. We have therefore revised our text to explain this apparent incongruity (page 11, lines 10-13).

2. Can the authors discuss/cite more about the actual subcellular compartments that the A3s are being relocated towards by the RNPs? In general, the authors' comments are limited to whether the A3 is predominantly in the nucleus, or not.

Previous imaging studies with markers for cytoplasmic organelles by our lab suggested that BORF2-A3B aggregates accumulate within the endoplasmic reticulum (ER) (PMID 30420783). However, in our recent cryo-EM studies of the BORF2-A3B complex (PMID 35476445), we discovered that disrupting BORF2-BORF2 dimerization prevents aggregate formation but does not affect EBV BORF2’s ability to bind to A3B and relocalize the complex to the cytoplasmic compartment. In other words, dimerization-deficient mutants of BORF2 clearly cause A3B-BORF2 heterodimers to appear diffusely cytoplasmic. Therefore, we no longer have a reason to implicate the ER and we aim to clarify this in future studies aiming to define the full molecular composition of the large cytoplasmic aggregates.

3. Since the authors draw a connection between the absence of A3B in New World Monkeys and the fact that New World Monkey-specific viruses don't seem to counteract A3s, can the authors discuss what could be learned by studying human individuals who lack A3B and the evolution of herpesviruses in those individuals?

This is a very interesting point, but we would prefer not to speculate on this in our manuscript. Although there is indeed an *A3B* deletion allele in the human population (predominantly southeast Asia), its worldwide allele frequency is quite low and most people still have 1 or 2 copies of this antiviral gene. Thus, the deletion allele frequency is not high enough to remove the selective pressure on the virus to maintain A3B counteraction activity through its RNR.

However, we did discover one Old World monkey species that completely lacks *A3B* (*Colobus angolensis*). We showed that the RNR from the γ-herpesvirus that infect these monkeys (ColHV-1) lacks the ability to antagonize human A3B, ancestral A3B, human A3A, or the endogenous A3A of its natural host (Figure 8 and Figure 8—figure supplement 3). Thus, as you predicted, relieving the selective pressure within a species over an evolutionary period of time likely resulted in loss of A3B-antagonism activity by the viral RNR (page 19, lines 8-16).

Minor points:1. I'm not sure it makes sense to call out Figures 1A-D in the Introduction section, rather than the Results section.

We have changed the Introduction and removed the original Figure 1 completely. We have however added a new Figure 1, which provides a structural rationale for our overall experimental approach.

Reviewer #1 (Significance (Required)):This work represents a step-wise advance from the authors' previous work on herpesvirus RNPs and counteraction of host APOBEC3s. I study host-virus molecular arms race on evolutionary scales and this article is of interest and significance to me, and I assume to others in the field as well. The findings found within the submission are interesting but not necessarily informative about human health and disease. However, the subsequent work that this manuscript inspires is likely to tell us more about herpesvirus evolution in human patients and the mechanisms by which APOBEC3s promote cancer.

We thank you again for appreciating the broader significance of our work and how the results present here may inspire important future studies.

Reviewer 2Reviewer #2 (Evidence, reproducibility and clarity (Required)):Summary: Building off the groups prior work on A3B and EBV BORF2 interactions, here they have expanded their studies to examine additional herpesvirus RNRs, demonstrating which features are conserved. Using a combination of IP experiments and IF, they have included KSHV ORF61 and HSV-1 ICP6 RNRs, and demonstrated that the A3 loop structures, L1, L3, and L7 from A3A, A3B, and A3G play varying roles in determining the ability to interact with the different RNRs. They then go on to demonstrate that the ability of BORF2 to block the deaminase activity of A3B is dependent on the tyrosine at position 481. Lastly, and most interestingly, they show that RNRs from Old World monkeys, but not New World monkeys, can bind to A3A and A3B, lead to their re-localization, and block deaminase activity.

We thank you for appreciating the molecular details and broader impact of our studies. Please note that we have revised the paper to focus on γ-herpesviruses by removing the less informative results with HSV-1 and adding new studies on Old/New World viral RNRs including comparisons with ancestral A3B.

Major comments: The vast majority of this work is very convincing. The authors claims are clearly reflected in the data presented for the most part. However, the work done with HSV-1 ICP6 co-IP is not very convincing. The authors claim that L7 and L3 swaps from A3Bctd to A3Gctd decreases pulldown (lines 5-12, p.7; lines 18-21, p.8; line 17, p.16). The figures (2A, 3A, 4A) however show only A3A being pulled down with ICP6. The re-localization data however does seem more consistent with the above claims. The authors note this in line 9, p.8. However, they come to a different conclusion in line 2, p.8, regarding the discrepancy between IP and IF data.

As mentioned above, we have removed the less convincing results with HSV-1 ICP6. We believe uncovering the mechanistic details of HSV-1 ICP6 interaction with A3B will require significant additional work and, therefore, would prefer to address this question in future studies.

The data and methods are clearly presented, with the exception of the supplemental figures, where it is unclear how the predicted modeling was conducted.

We apologize for the brief description in our earlier submission. We have revised our Methods section and included a more detailed description regarding the generation of protein structural models (page 29, lines 20-23; page 30, lines 1-6).

Experiments all seem to be sufficiently replicated.

Thank you.

Minor comments:The references to prior studies seem comprehensive. Text and figures were all very clear. Introducing the supplemental figure 1 earlier, may provide clarity to the argument about degree of relatedness (line 2, p.7).

We agree with this suggestion and have made changes to introduce the structural model of KSHV ORF61 in our new Figure 1.

The suggestion of ORF61 interaction with L3 as an anchor region (line 10-12, p.9) was not very clear/could benefit from a bit more elaboration.

We agree with this comment and have placed the predicted structural model of KSHV ORF61 bound to A3Bctd in our new Figure 1 and we have changed the text to clarify the role of A3B L3 in binding to KSHV ORF61 (page 10, lines 4-8).

Reviewer #2 (Significance (Required)):This work builds on the conceptual framework of host-pathogen interactions and co-evolution, adding new examples of co-divergence of primate herpesviruses with their respective host restriction factors. Following up on past findings (Cheng et al., 2019; Shaban et al., 2021), and reports from others (Stewart et al., 2019), they outline the degree to which their initial findings (BORF2 and A3B interactions) are conserved across other herpesvirus RNRs, and place them in the context of the evolution of the A3 gene locus and expansion.This work will be of great interest to virologists. Especially those that work in the field of host pathogen evolution and the molecular arms race.My background is in host-pathogen interactions and herpesvirus evolution. I lack the sufficient expertise to evaluate the predicted modeling.

We thank you again for appreciating the novelty and significance of our work. We are also hopeful that it will be of great interest to virologists.

Reviewer 3Reviewer #3 (Evidence, reproducibility and clarity (Required)):In this manuscript, Moraes and colleagues build upon previous publications from this group to (1) characterize the variation in the ability of orthologs of BORF2 from six different herpesviruses to bind and/or relocalize and/or inhibit the deaminase activity of A3A and A3B; (2) use swaps and other mutagenesis to measure whether various regions and amino acids in A3A, A3B, and A3G contribute to the observed variation in the ability of RNR subunits from different viruses to bind these A3s.The data convincingly show that different regions of different A3s contribute differently to binding of RNRs from different viruses. These same regions also have variable effects on RNR-mediated relocalization and inhibition of the deamination activity of A3A, A3B, and A3G. In the last set of experiments presented in the manuscript, the authors show that the RNR from the four viruses isolated from humans and rhesus macaques are able to bind human A3B, while the RNRs from two New World monkey viruses are unable to bind human A3B. Finally, the authors suggest a correlation between the timing of the birth of A3B in the branch leading to the last common ancestor of hominoids/Old World monkeys and the gain of A3 binding/antagonism by herpesvirus RNRs. However, these evolutionary implications are not convincingly supported by the current datasets and would require a significant burden of initial experiments to test.

We thank the reviewer for the nice summary of our work and for appreciating the loop swaps experiments showing differential RNR binding to APOBEC3s. In our original submission, we compared the RNRs of 4 viruses infecting Catarrhini primates and 2 viruses infecting New World primate species. We found that only the RNRs from viruses that infect Catarrhini primates bind, relocalize, and inhibit human A3B. We have now performed additional experiments to further investigate this remarkable association (Figures 7, 8, and associated supplementary material) which are detailed in our revised manuscript and summarized below:

First, we have expanded the scope of our experiments to include all publicly available RNR sequences from primate γ-herpesviruses (*i.e*., 11 RNRs in contrast to our initial 6 RNRs). Second, we tested this whole panel against human A3B and found that only the RNRs from viruses that infect Old World primates that encode *A3B* are able to bind, relocalize, and inhibit human A3B (Figures 6 and 8). In comparison, binding to human A3A in co-IP experiments is invariably weaker and/or not detectable, relocalization phenotypes are less pronounced, and DNA deaminase activity is not inhibited (Figure 6 and Figure 8—figure supplement 2B). Third, a subset of this RNR panel was tested against the A3A enzymes of their natural host species (Figure 7) and, again, only the RNRs form viruses that infect Old World primates bind and relocalize the A3A enzymes tested. Fourth, as an addition test of this idea, we grafted a short helical loop structure (HLS) from EBV BORF2 into the marmoset CalHV-3 RNR and showed that this small change enabled the chimeric protein to bind to both human A3B and A3A (likely through L7), though not to the natural marmoset A3A protein. Fifth, we used all available present-day primate A3B sequences to reconstruct the most likely ancestral A3B sequence and showed that this enzyme is nuclear and as active (if not more active) than human A3B (Figure 8). This ancestral A3B protein is also bound, relocalized, and inhibited by most present day RNRs from γ-herpesviruses that infect species with A3B, but not by the RNRs of any of the NWM-infecting viruses tested. The only exception to this association between A3B and Catarrhini-infecting γ-herpesviruses is the RNR of the African Colobus virus ColHV-1, which we found can likely be explained by the loss of *A3B* in its host species due to a deletion that occurred approximately 10-14 mya after the split of the *Colobinae* subfamily into African and Asian tribes, which further supports the idea that A3-antagonism by γ-herpesvirus RNRs is maintained by the selective pressure imposed by the antiviral activity of A3B.

Major Comments1) The use of only the human orthologs of A3A and A3B limit the inferences that can be made regarding the ability of RNRs from various viruses to bind the A3s from the host species of that virus. For example, human A3A (and other hominoid A3As) have a rather distinct Loop 1 sequence, where that same loop in rhesus A3A is a much more similar to A3B. It follows that the RNRs from rhesus-tropic viruses could very well bind and inhibit A3A from rhesus. Likewise, the A3B-RNR interactions within and between species could differ markedly. Indeed, we know that the loops of A3s are some of the most rapidly evolving regions of these genes.

We agree fully with these points and have addressed them through several new experiments. We have now tested the RNRs from rhesus macaque and NWM γ-herpesviruses against the A3A enzymes of their natural host species (Figure 7) and found that only RNRs form viruses that infect Old World primates bind and relocalize the A3A enzymes tested. In addition, we used all available present-day primate A3B sequences to reconstruct the most likely ancestral sequence and showed that this ancestral A3B enzyme is antagonized exclusively by the RNRs of present-day γ-herpesviruses that infect A3B-encoding primates.

2) If RNR's ability to bind A3s correlated or was driven by the birth of A3B in catarrhine primates, the evolution of the binding/antagonism trait would be highly unparsimonious. The most parsimonious scenario would be emergence of A3 antagonism in the LCA of α and gammaherpesviruses (since the authors show A3 binding in HSV-1 and several gammaherpesviruses) with a loss of the trait in NWM-infecting viruses; alternatively, the trait could have been horizontally transferred gained 3 independent times, but this is certainly unlikely and not supported by any data. However, it is also possible that the RNRs from NWM infecting viruses do, in fact, bind/antagonize the A3 orthologs from NWMs. This needs to be tested before addressing the complexities of the birth of the antagonism trait.

Please see our responses above. All of our results support a model in which the birth of *A3B* in an ancestral primate selected for a γ-herpesvirus with A3B binding and neutralization activity and that this activity has been maintained through evolution and still manifests today in all of the tested present-day RNRs of γ-herpesviruses that infect species with A3B.

Minor Comments1) The authors should state more discreetly what is new to this paper and what was shown in previous paper and in some cases repeated here. For example, figure 1 is all repeated experiments from previous papers which is unusual for a manuscript.

This is a fair point and we have removed the original Figure 1 and replaced it with a structural model that provides a strong rationale for the rest of our studies. For the sake of clarity, we have also revised our text and made the necessary changes to ensure a clear distinction between new and repeated results.

2) The authors conclude that RNRs bind to A3s via partially distinct surfaces, but they don't actually test binding. Swaps and mutations do not show that the site of mutation is a site of interaction. but they do test the requirement of these AAs or regions for binding. Formally, these mutations could be exerting an allosteric effect on the binding interface of RNR and A3. In combination with the CryoEM data, these new data do support the model that these are different surfaces of interaction, but the wording should be more precise to present this.

In our revised manuscript we use a combination of *in silico* protein structure prediction and docking to model the binding interface between human A3Bctd and KSHV ORF61 (new Figure 1). This approach predicts an interaction with the L3 region of A3B, which we validate through co-IP and co-localization experiments (Figure 3). In contrast, EBV BORF2 requires the L7 region to bind to A3Bctd and this interaction is additionally strengthened by L1 residues (Figures 2 and 4). Taken together with our prior cryo-EM data, these results point to a model in which EBV BORF2 and KSHV ORF61 bind to different surfaces of A3B (albeit near the active site and likely due to evolutionary “wobbling”). We therefore believe it to be unlikely that this mechanism is allosteric given our prior structural studies and the likely common evolutionary origin of this A3B antagonism mechanism.

3) Similar to point 1, the authors repeatedly discuss the "most critical determinant of EBV BORF2 binding" and other "most critical" interactions. This is not supported by the data and should be changed to something along the lines of 'the site of largest effect among the sites we analyzed'.

We have endeavored to change this text as suggested except in cases referring to the interactions between EBV BORF2 and A3Bctd, since the results presented here together with our cryo-EM structure of the BORF2-A3Bctd complex (PMID 35476445) allow us to confidently say that L7 and L1 are the most critical determinants.

4) All microscopy figures need an A3 only panel (no RNR) to be able to judge relocalization.

Changed as suggested.

5) The matrix of labels above each IP blot is excessive since each lane only has one component that differs from the other lanes. A single label for each lane would make the plot easier to discern. These figures would also benefit from clearer labels including which virus each blot panel corresponds to (these could be along the left side of each blot; currently, the RNR gene name is provided, but this is a bit hard to find within the figure). Figures 2-4 would benefit from a label above each panel A indicating "L1" "L3" "L7".

Changed as suggested.

6) If the authors comment on pg9 ln 8 about intermediate relocalization effect, they should also mention 1C A3B L7G against BORF2.

Changed as suggested (page 8, lines 16-19).

7) Why is there no quantification of 6D relocalization? Could be supplemental if needed.

We have performed quantification of the relocalization phenotypes in Figures 2, 3 and 4 in order to allow direct comparison between WT and chimeric A3 enzymes in the presence of the same viral RNR (EBV BORF2 or KSHV ORF61). On the other hand, the images in Figure 6D are representative of the A3B/A relocalization phenotypes elicited by a larger panel of different viral RNRs. These representative images should be interpreted together with the co-IP and ssDNA deaminase activity assay data in Figures 6C and 6E, respectively.

8) Pg 6 ln 13 and Pg 8 ln2-4, ICP6 doesn't coIP w A3B; this should be clarified.

A similar concern was also raised by reviewer #2, and we agree that the ICP6 data present in the original version of this manuscript are not as easily interpretable compared to results with the RNRs from γ-herpesviruses such as EBV and KSHV. For the sake of clarity and cohesion, we decided to remove all of the HSV-1 ICP6 data from the revised version of our manuscript and focus on the A3B interactions with γ-herpesviruses.

9) Pg 8 ln21-23, the authors assume loss of function for A3G, but this swap could be functionally equivalent, but necessary for binding; it should be clarified that this is different than changing sequence and still binding.

Rephrased (page 9, lines 13-16).

10) Pg 9, ln 11, what is an anchor region?

We have removed the term “anchor region” and rephrased our text to more clearly describe the importance of L3 in KSHV ORF61 binding (page 10, lines 4-8).

11) Pg 9, ln 23, speculative – this might be explained by this 3 AA motif but it has not been tested.

Changed wording (page 10, lines 17-20).

12) Pg 10 ln 8, this doesn't show that this region is dispensable for binding, only that there is equivalent contribution or lack of contribution of function by the A and B loops, again assuming that the G loop is LOF.

Removed the phrase “indicating that this region may be dispensable for the interaction” (page 11, 1-2).

13) Pg 10 ln 10 – "can be explained by presence of bulky tryp" – this should be reworded to 'could or is likely caused by'.

Changed as suggested (page 11, lines 4-6).

14) Pg 11 ln 21, "can be explained by our cryo-EM" should be reworded to 'is supported by these contacts in cryo-EM'

Changed as suggested (page 12, lines 15-18).

15) Pg 13 ln 10 (and other places) dissociation rates are only part of affinity, Ka is equally important (pg 18 ln 8 also).

We agree and have revised our text account for this suggestion (page 13, lines 22-23; page 14, lines 1-2; page 22, lines 12-15).

16) Pg 14, ln 15 should be reworded to 'relocalize HUMAN cellular A3s'.

Changed as suggested (page 15, line 11).

17) Pg 16 Ln 16, this should be reworded as the data can't say it is completely dispensable without deletion of the loop.

Changed as suggested (page 21, lines 10-11).

18) New World monkeys have high activity of transposable elements of distinct types relative to catarrhines. It would be useful to mention that A3s restrict endogenous elements as well and how this might be a factor in the proposed evolutionary model.

This is a very interesting point that we plan to discuss in a future review. In addition, many future experiments will be needed to test the potential relationship between the birth of *A3B* and its potential impact on different classes of endogenous transposable elements.

19) Are the New World monkey viruses pathogenic in their native hosts? Perhaps not based on previous literature (reviewed in PMID: 11313011). This should be included in the discussion as it could certainly effect the evolutionary model for the birth/retention of A3 antagonism in these viruses.

While interesting, the observation that NWM herpesviruses do not cause disease in their native hosts is not unusual. In fact, most γ-herpesviruses (including human viruses like EBV and KSHV) have limited pathogenic potential when infecting their natural hosts (Fleckenstein B, Ensser A. Gammaherpesviruses of new world primates. Human Herpesviruses: Biology, Therapy, and Immunoprophylaxis. 2007). Additionally, although the mentioned study (PMID: 11313011) reports asymptomatic infection of squirrel monkeys with SaHV-2, pathogenic infection/ oncogenic transformation have been reported following natural infection of marmosets with CalHV-3 (PMID: 11158621).

20) In previous papers on this topic, the lab has tested the effect of mutations on viral titers. While this may be beyond the scope of this paper, this would certainly elevate the paper and should be more clearly discussed.

As noted by the reviewer, we have previously demonstrated that A3B restricts EBV replication though a mutation-dependent mechanism and that this is counteracted by EBV BORF2 (PMID 30420783). While we completely agree that investigating the effect of A3B-catalyzed mutations on the titers of different γ-herpesviruses would be interesting, this would be technically challenging as we are currently not equipped to work with KSHV or any non-human primate herpesvirus.

21) What is the degree of sequence similarity among these and other RNRs? Is there any sense of what region of RNR binds A3s from the CryoEM structures and differences within these regions that might explain the functional differences?

We thank the reviewer for raising this important point. We have now included a new Figure 1 where we leverage the cryo-EM structure of the EBV BORF2-A3Bctd complex to make inferences about which regions of KSHV ORF61 may be involved in binding A3B/A. As described above, we also graft a short helical loop structure (HLS) from EBV BORF2 into the marmoset CalHV-3 RNR and showed that this small change enables the chimeric protein to bind to bind both human A3B and A3A (likely through L7), though not to the natural host marmoset A3A protein (Figure 7). Many additional interspecies chimeras could be constructed but we feel these are better suited for future studies (and specially to accompany future structural work in this area).

Reviewer #3 (Significance (Required)):SignificancePrevious work from the Harris lab showed that a subunit of the ribonucleotide reductase of some herpesviruses acts as an antagonist of several human APOBEC3s. Mechanistically, these viral protein block A3 inhibition by relocalizing nuclear A3s as well as inhibiting A3 deamination by binding and occluding the A3 active site. For Epstein-Barr virus, deletion of the antagonist (BORF2) results in a decrease in viral replication and accumulation of mutations likely introduced by host A3B that is no longer inhibited. However, deletion of the A3 antagonist from herpes simplex virus^-1^ (ICP6) had no effect on viral titers. Most recently, this group published a cryoEM structure of BORF2 in complex with the c-terminal half of A3B. This structure showed extensive contacts between BORF2 and two loops of A3B – L1 and L7.The manuscript under review focuses on the previously suggested differences in the ability of different RNRs to bind A3A and A3B. This work provides an important contribution to this topic in defining specific regions of A3A and A3B and A3G that are necessary for viral RNRs to bind them. The variability in these interactions is surprising and likely testament to the impactful coevolution of herpesviruses and primate A3s. This manuscript will be of particular interest to virologists studying A3s or herpesviruses as well as evolutionary biologists interested in the rules of engagement between host restriction factors and viruses.

We thank you again for these thoughtful comments and for appreciating the overall significance of our work.