# Supplementary material for: Evidence linking APOBEC3B genesis and evolution of innate immune antagonism by gamma-herpesvirus ribonucleotide reductases
Source: eLife. 2022 Dec 2;11:e83893. doi: 10.7554/eLife.83893 (PMC9747160; doi:10.7554/eLife.83893)
Supplement: Figure 2—source data 1. [file elife-83893-fig2-data1.zip › Figure 2-source data 1/Figure 2-source data 1-uncropped.pdf]

Figure 2B

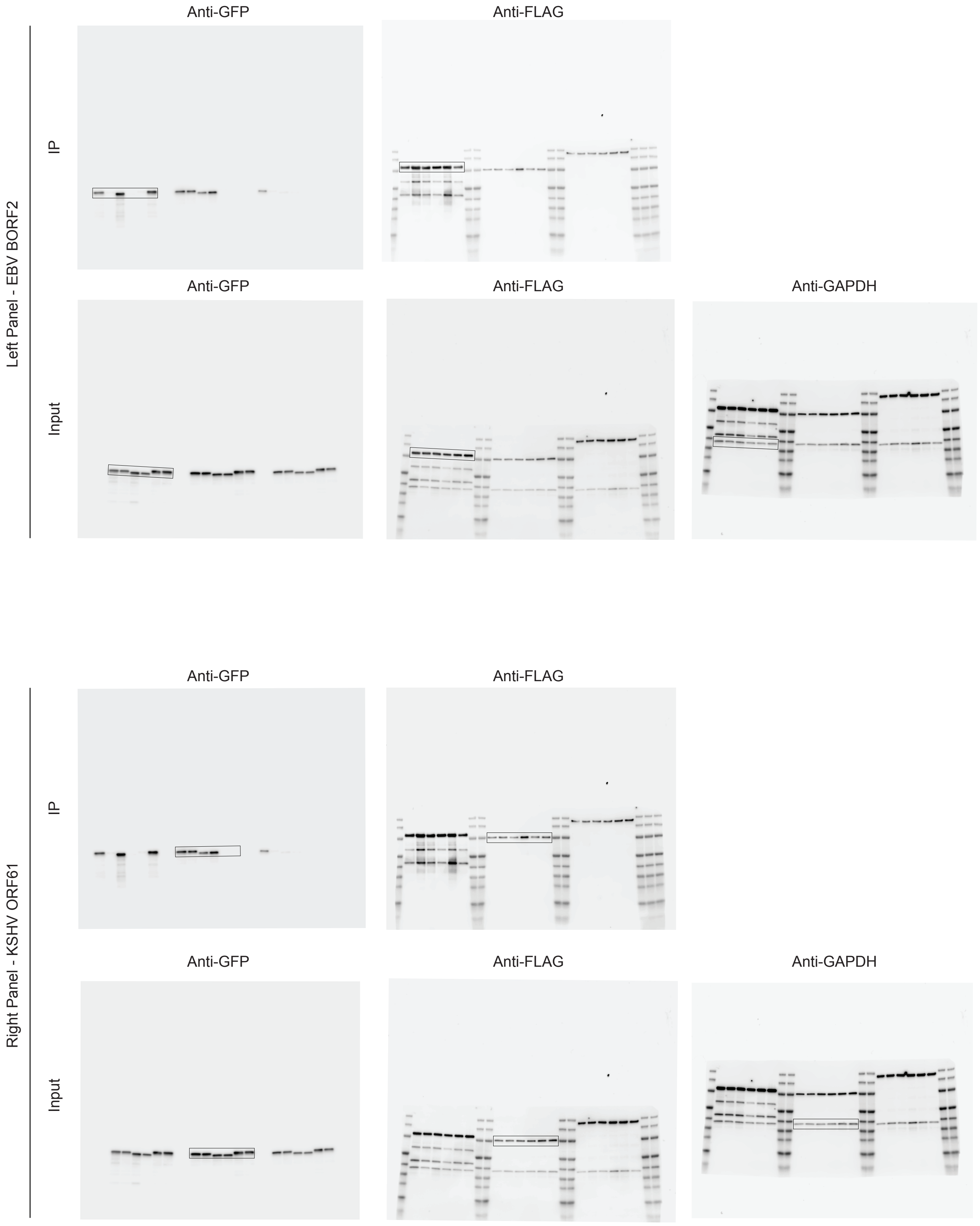
Left Panel - EBV BORF2
IP
Input
Anti-GFP
Anti-FLAG
Anti-GFP
Anti-FLAG
Anti-GAPDH
Right Panel - KSHV ORF61
IP
Input
Anti-GFP
Anti-FLAG
Anti-GFP
Anti-FLAG
Anti-GAPDH
