# Supplementary figures and images for: Evidence linking APOBEC3B genesis and evolution of innate immune antagonism by gamma-herpesvirus ribonucleotide reductases
Source: eLife. 2022 Dec 2;11:e83893. doi: 10.7554/eLife.83893 (PMC9747160; doi:10.7554/eLife.83893)

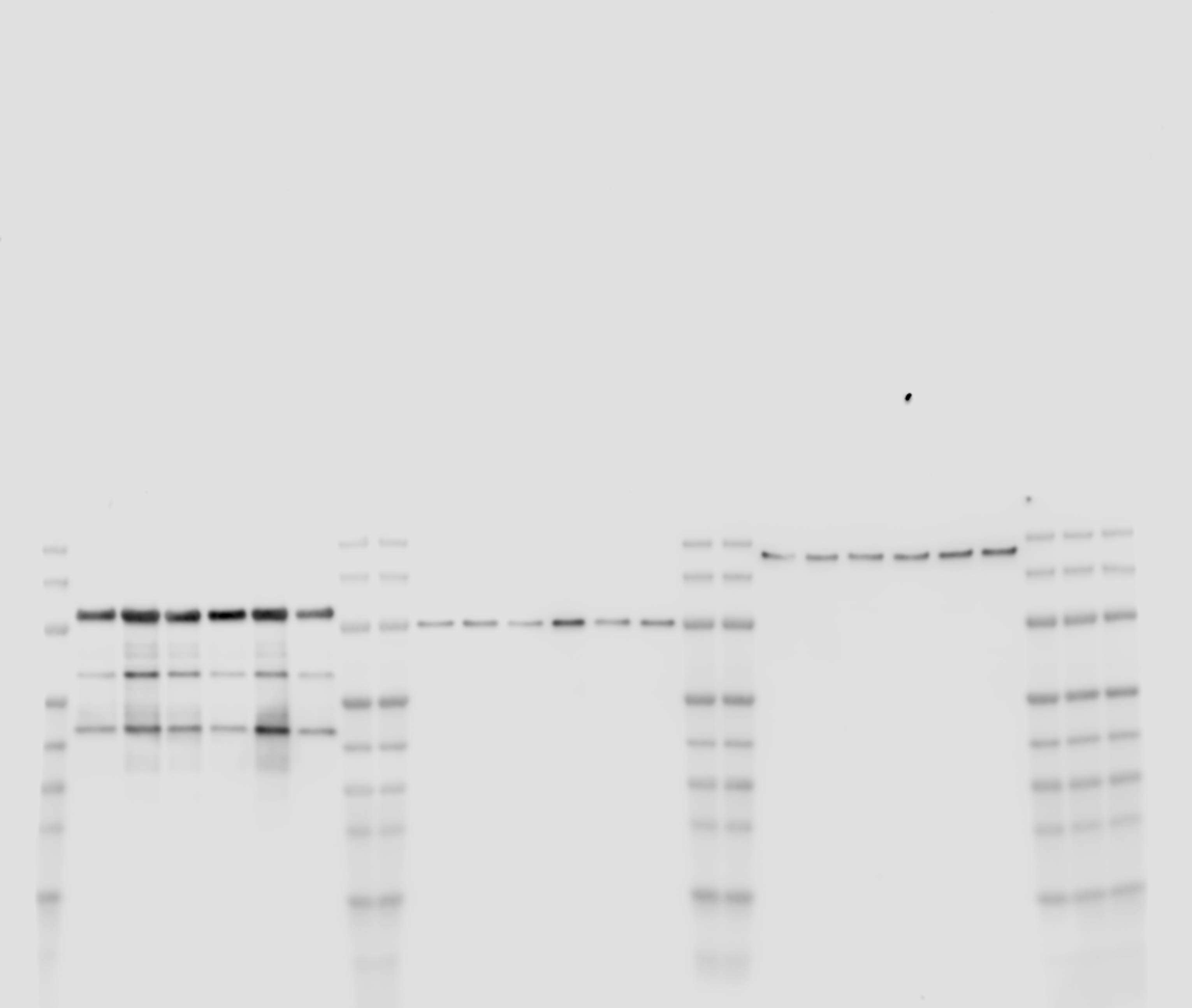

Supplement: Figure 2—source data 1. [file elife-83893-fig2-data1.zip › Figure 2-source data 1/Figure 2-source data 1-raw files/Figure 2-source data 1-IP FLAG channel.tif]

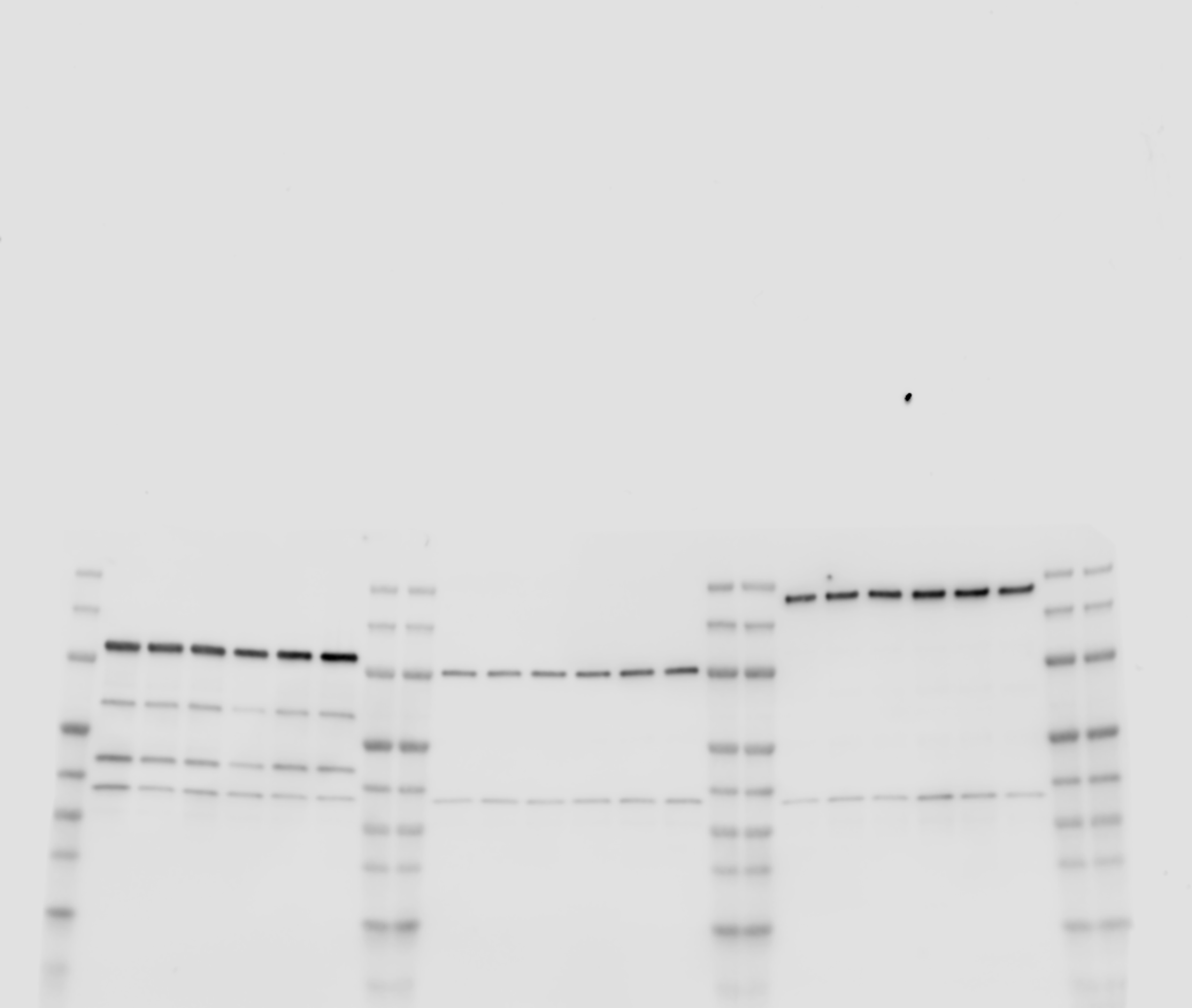

Supplement: Figure 2—source data 1. [file elife-83893-fig2-data1.zip › Figure 2-source data 1/Figure 2-source data 1-raw files/Figure 2-source data 1-input FLAG.tif]

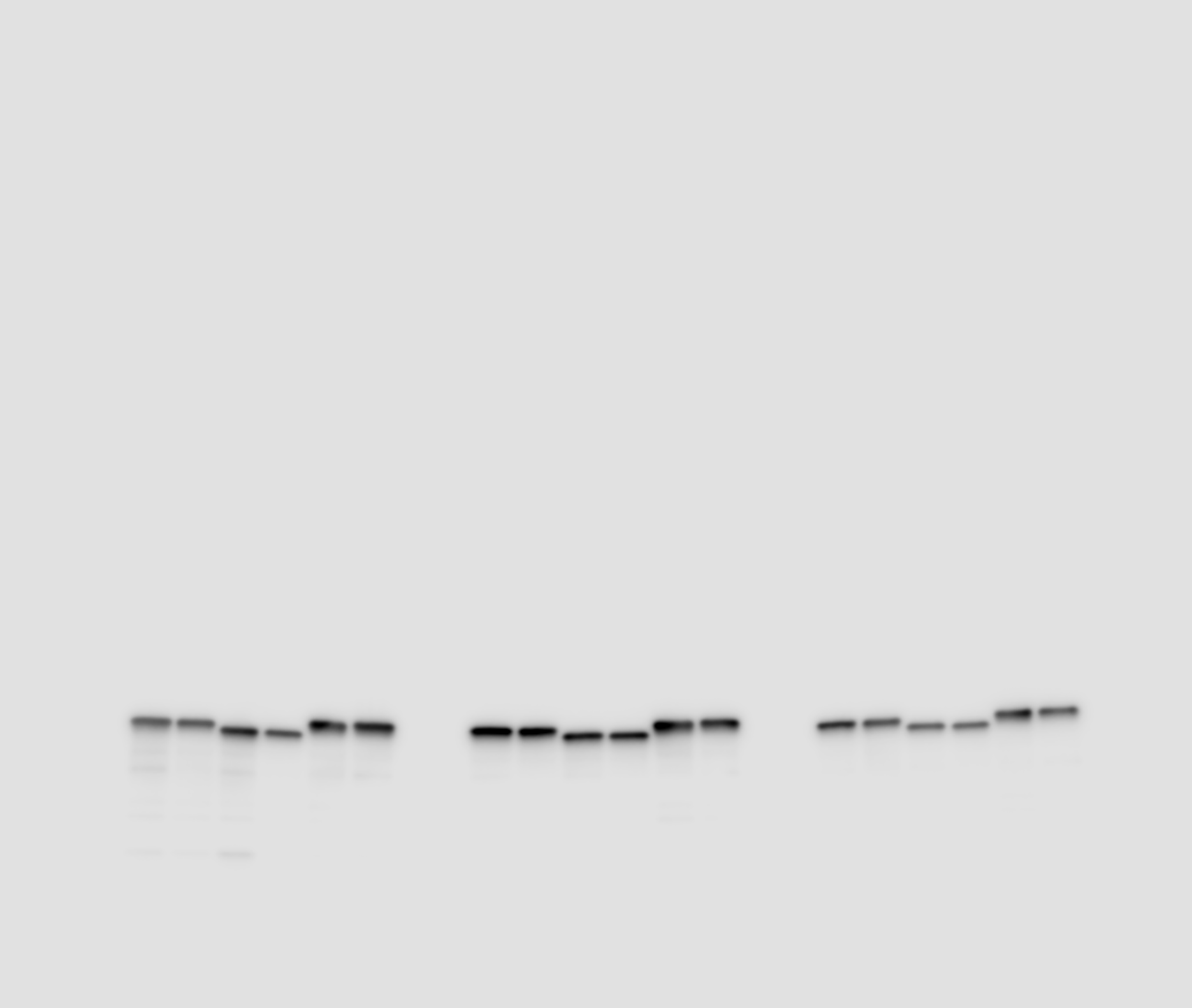

Supplement: Figure 2—source data 1. [file elife-83893-fig2-data1.zip › Figure 2-source data 1/Figure 2-source data 1-raw files/Figure 2-source data 1-input GFP channel.tif]

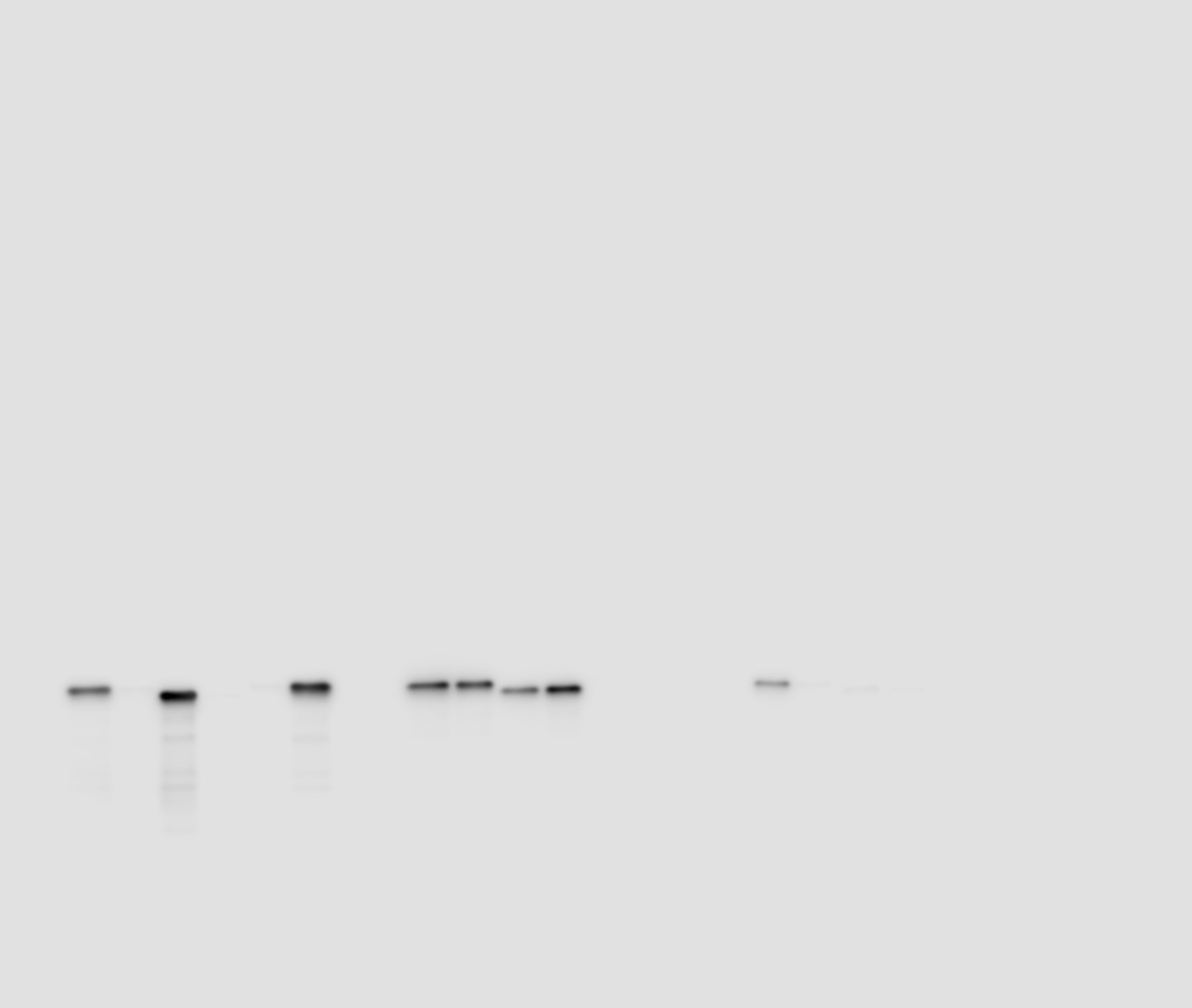

Supplement: Figure 2—source data 1. [file elife-83893-fig2-data1.zip › Figure 2-source data 1/Figure 2-source data 1-raw files/Figure 2-source data 1-IP GFP channel.tif]

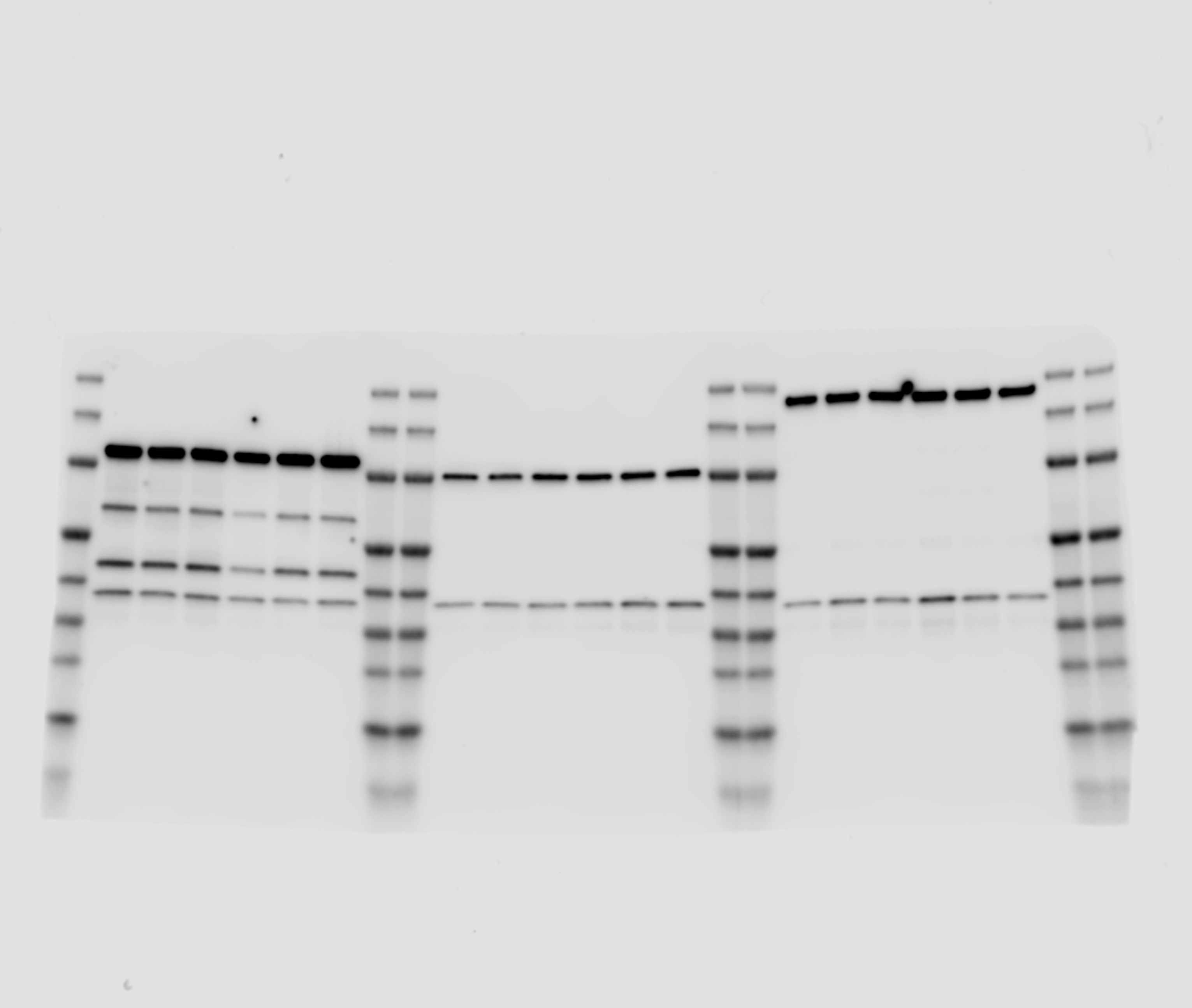

Supplement: Figure 2—source data 1. [file elife-83893-fig2-data1.zip › Figure 2-source data 1/Figure 2-source data 1-raw files/Figure 2-source data 1-input GAPDH channel.tif]

Figure 3B

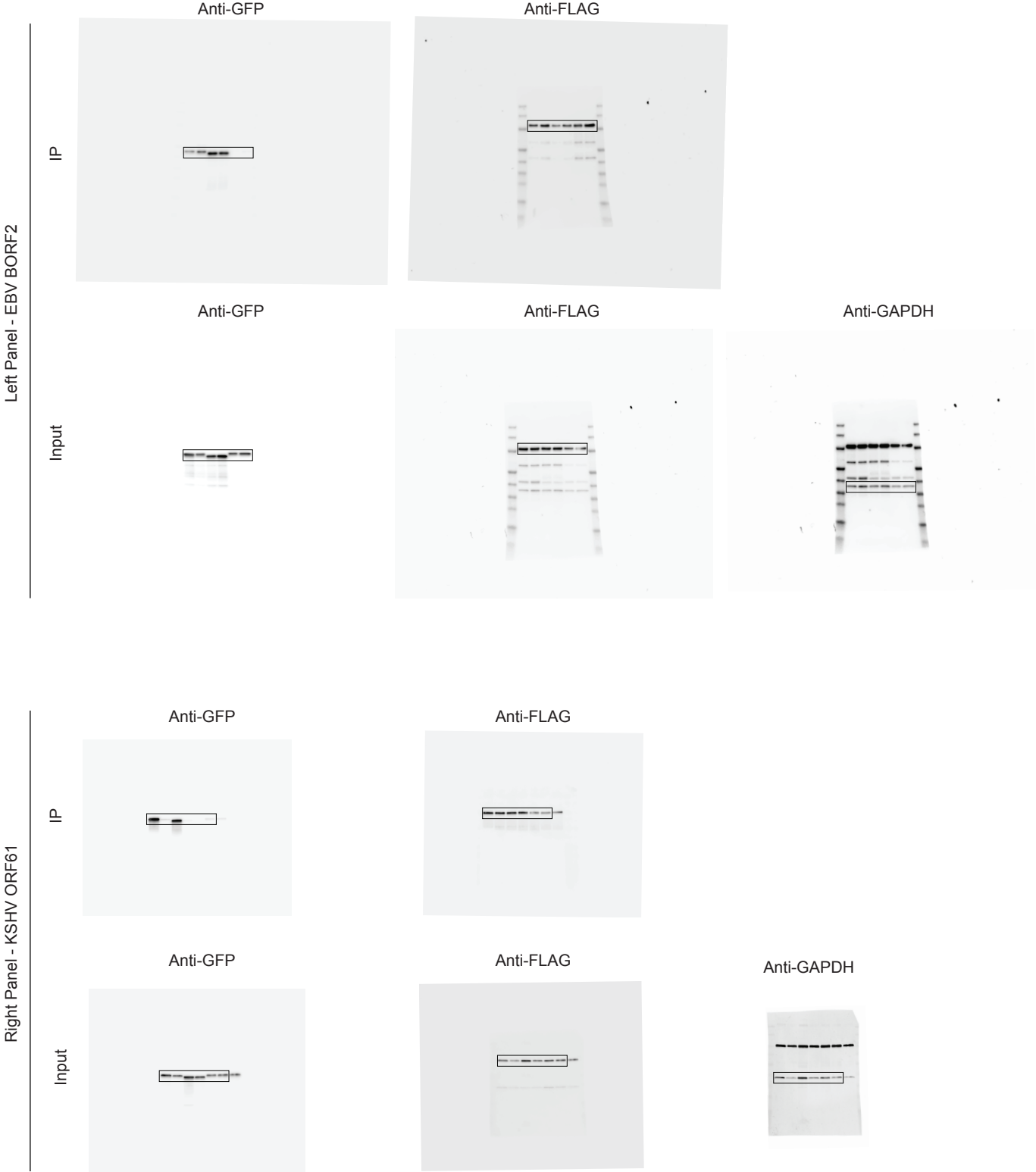

Supplement: Figure 3—source data 1. [file elife-83893-fig3-data1.zip › Figure 3-source data 1 /Figure 3-source data 1-uncropped.pdf]

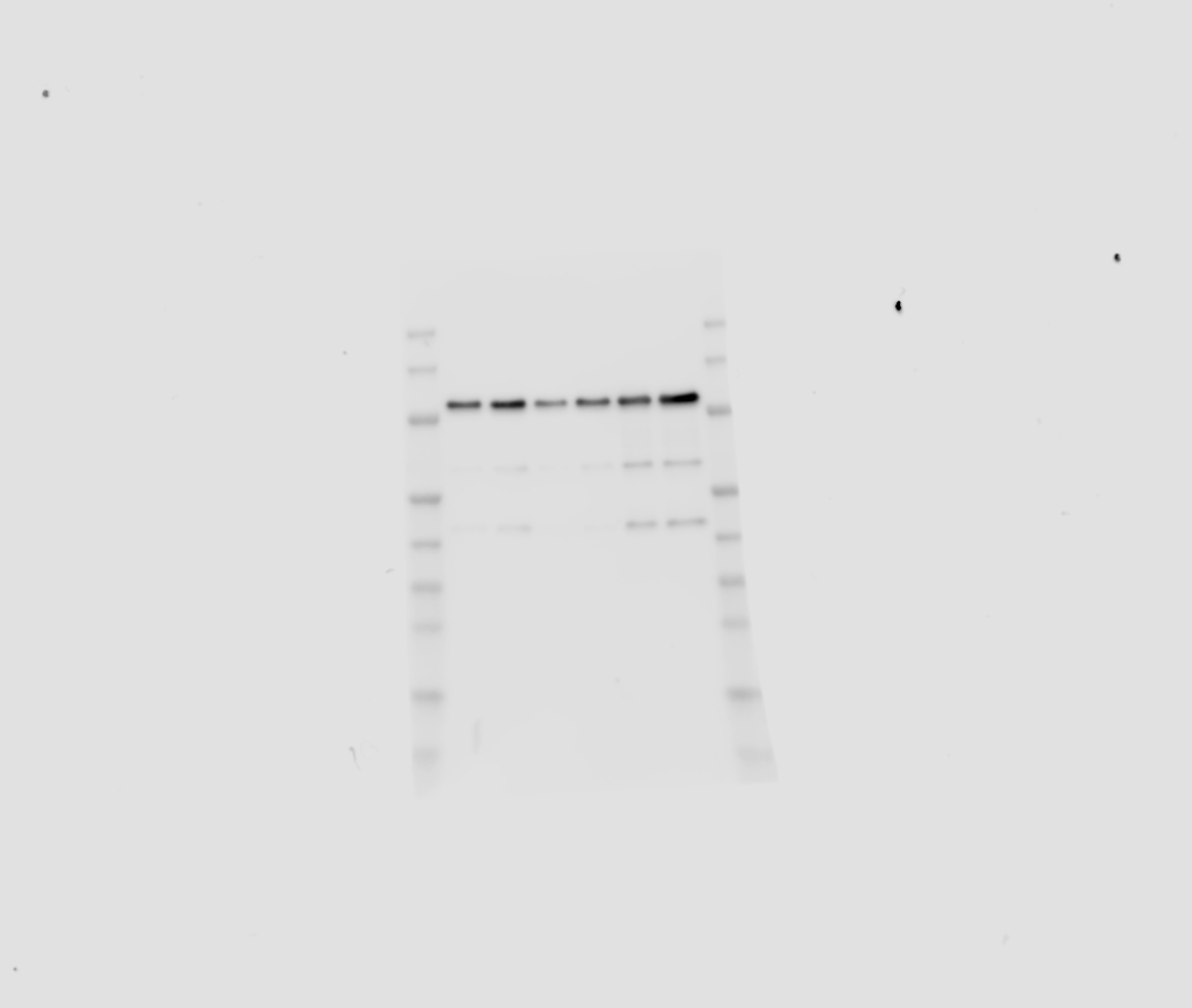

Supplement: Figure 3—source data 1. [file elife-83893-fig3-data1.zip › Figure 3-source data 1 /Figure 3-source data 1-raw files/Figure 3-source data 1-left panel-IP-FLAG channel.tif]

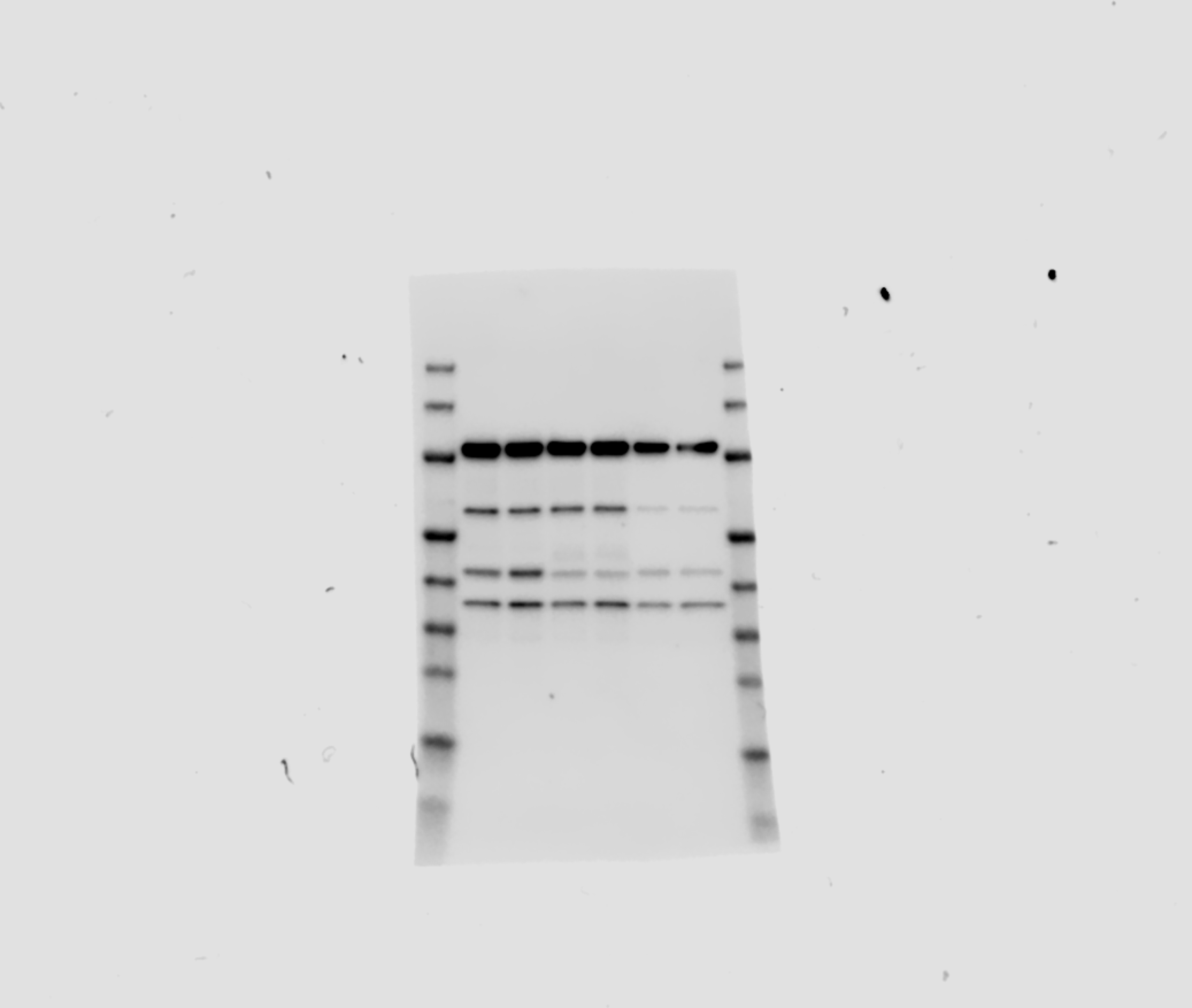

Supplement: Figure 3—source data 1. [file elife-83893-fig3-data1.zip › Figure 3-source data 1 /Figure 3-source data 1-raw files/Figure 3-source data 1-left panel-input-GAPDH channel.tif]

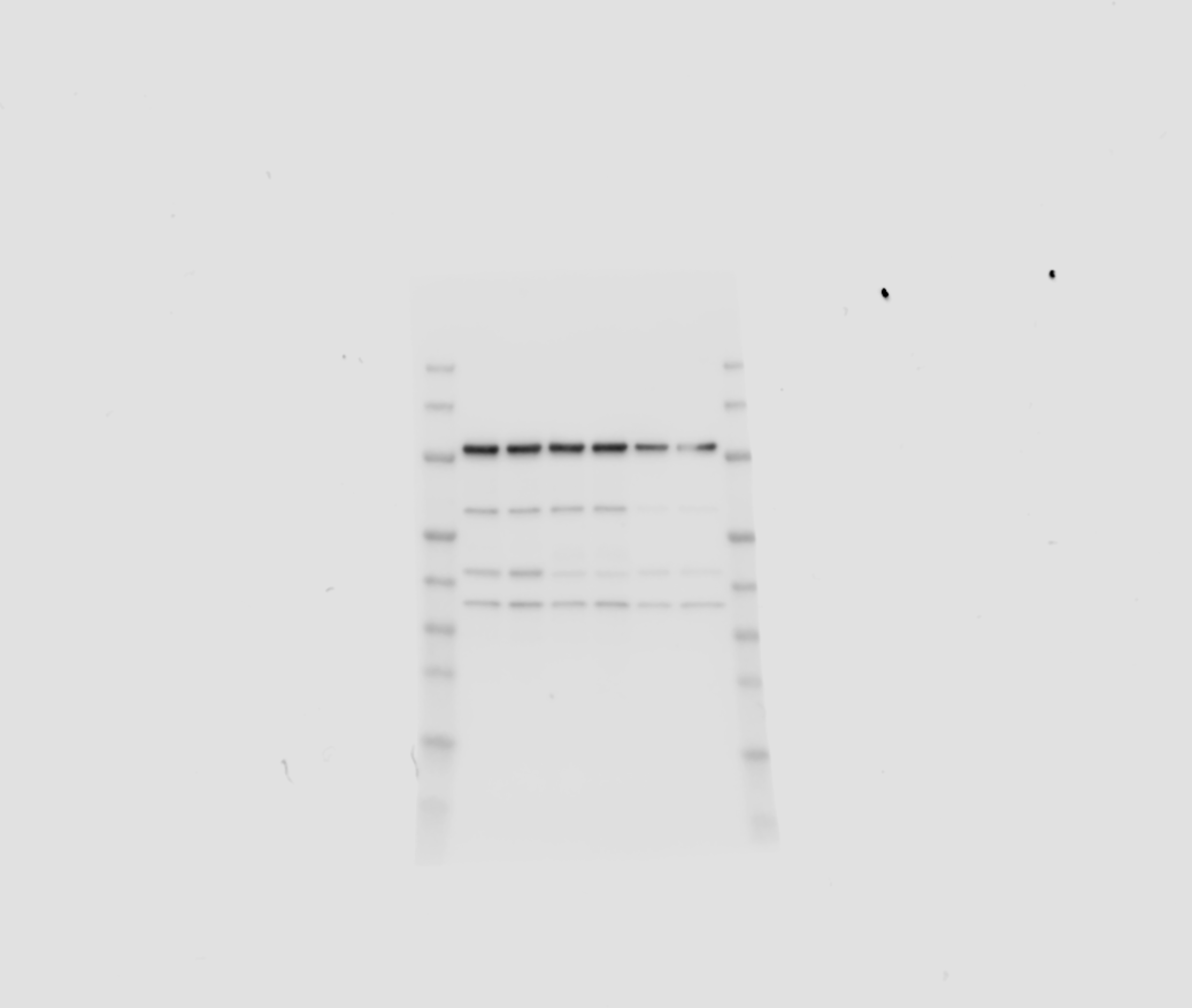

Supplement: Figure 3—source data 1. [file elife-83893-fig3-data1.zip › Figure 3-source data 1 /Figure 3-source data 1-raw files/Figure 3-source data 1-left panel-input-FLAG channel.tif]

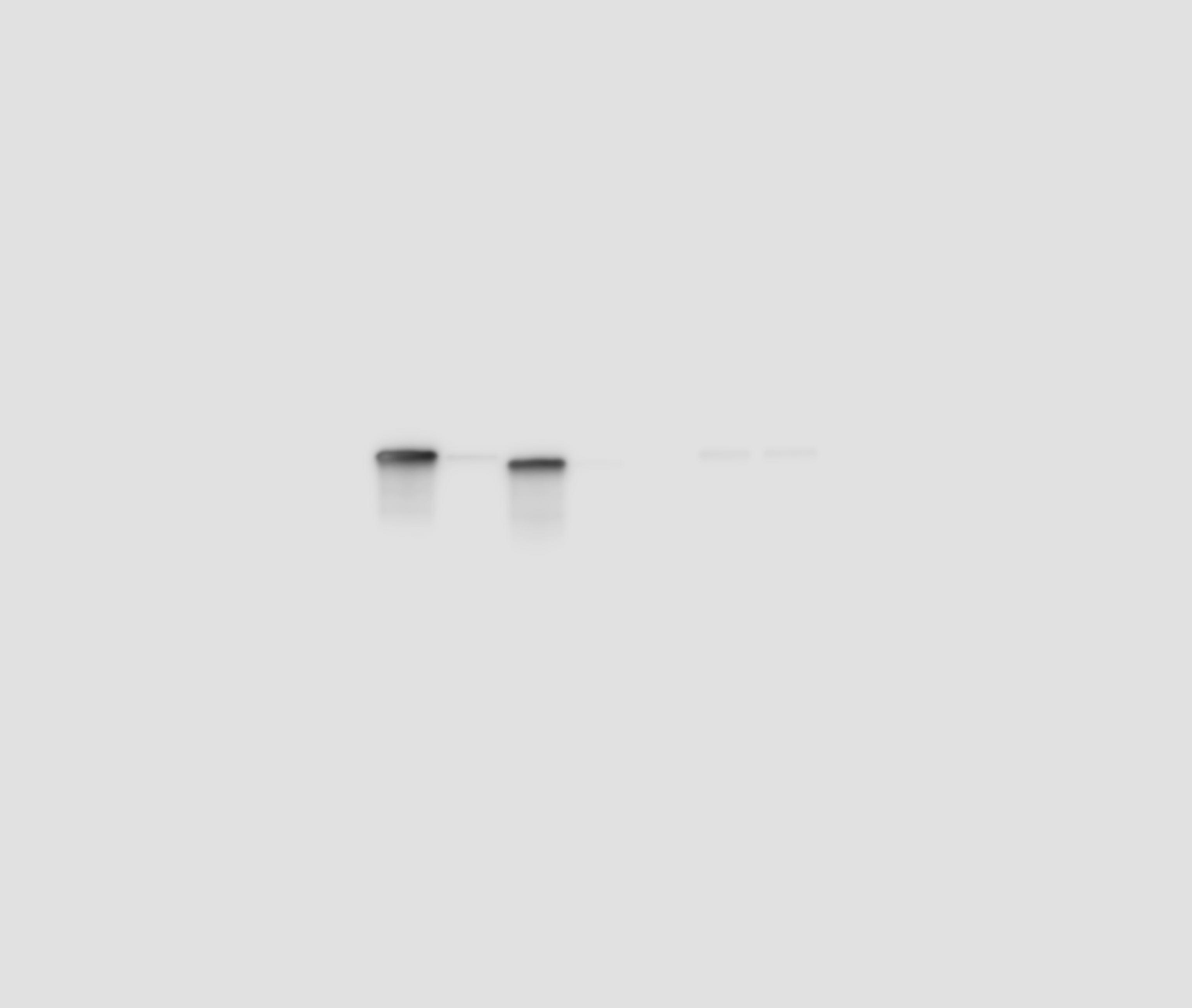

Supplement: Figure 3—source data 1. [file elife-83893-fig3-data1.zip › Figure 3-source data 1 /Figure 3-source data 1-raw files/Figure 3-source data 1-right panel-IP-GFP channel.tif]

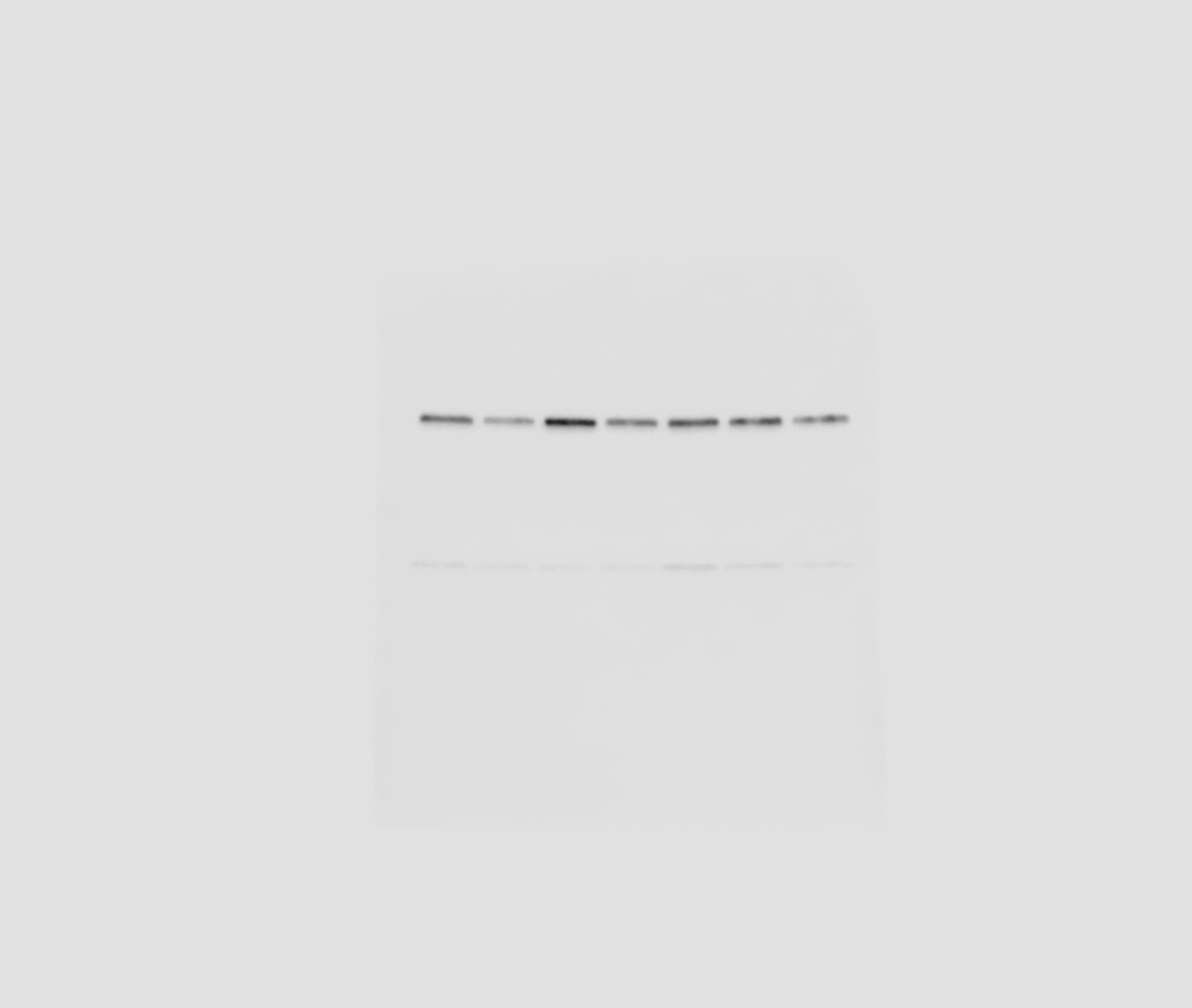

Supplement: Figure 3—source data 1. [file elife-83893-fig3-data1.zip › Figure 3-source data 1 /Figure 3-source data 1-raw files/Figure 3-source data 1-right panel-input-FLAG channel.tif]

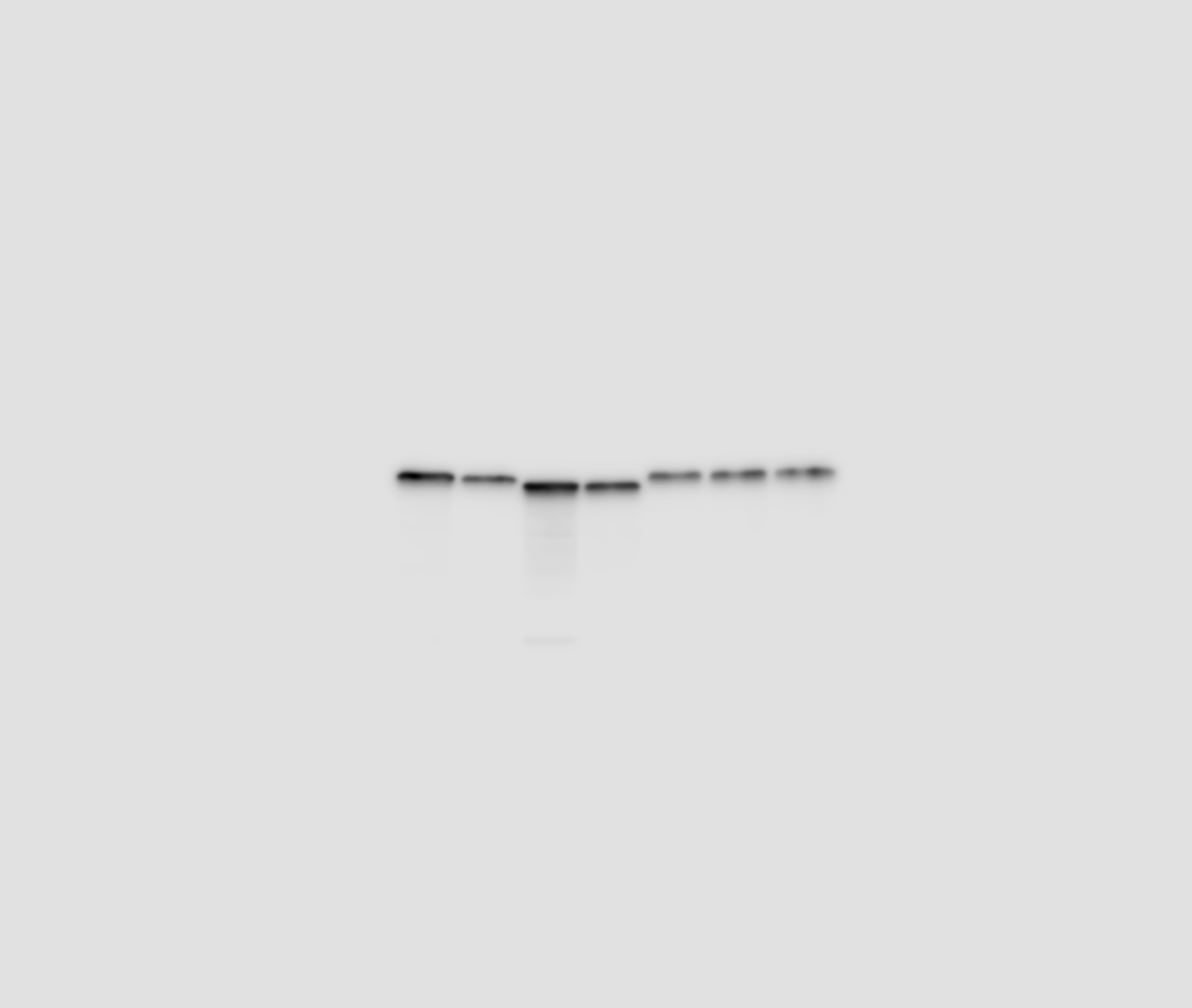

Supplement: Figure 3—source data 1. [file elife-83893-fig3-data1.zip › Figure 3-source data 1 /Figure 3-source data 1-raw files/Figure 3-source data 1-right panel-input-GFP channel.tif]

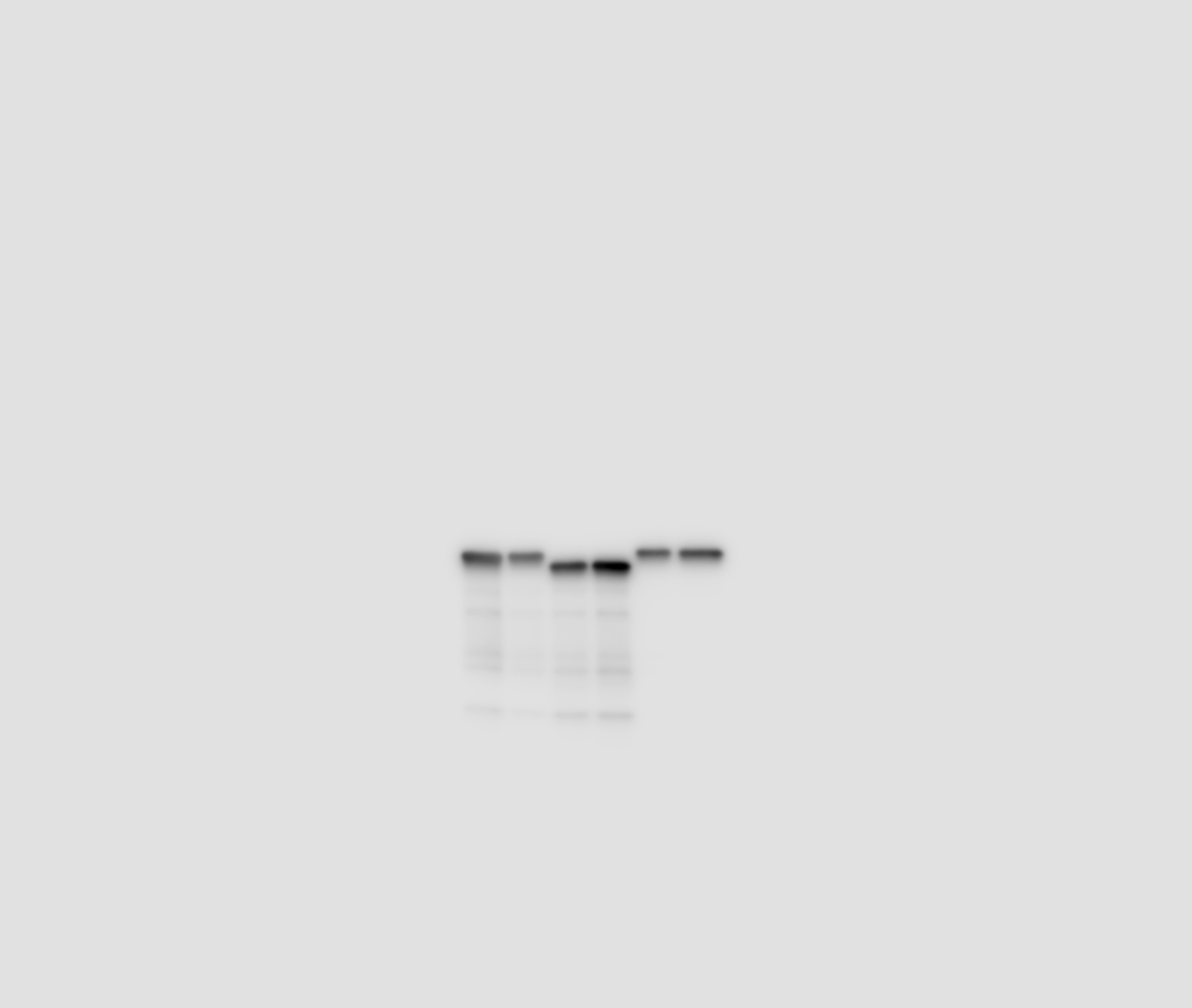

Supplement: Figure 3—source data 1. [file elife-83893-fig3-data1.zip › Figure 3-source data 1 /Figure 3-source data 1-raw files/Figure 3-source data 1-left panel-input-GFP channel.tif]

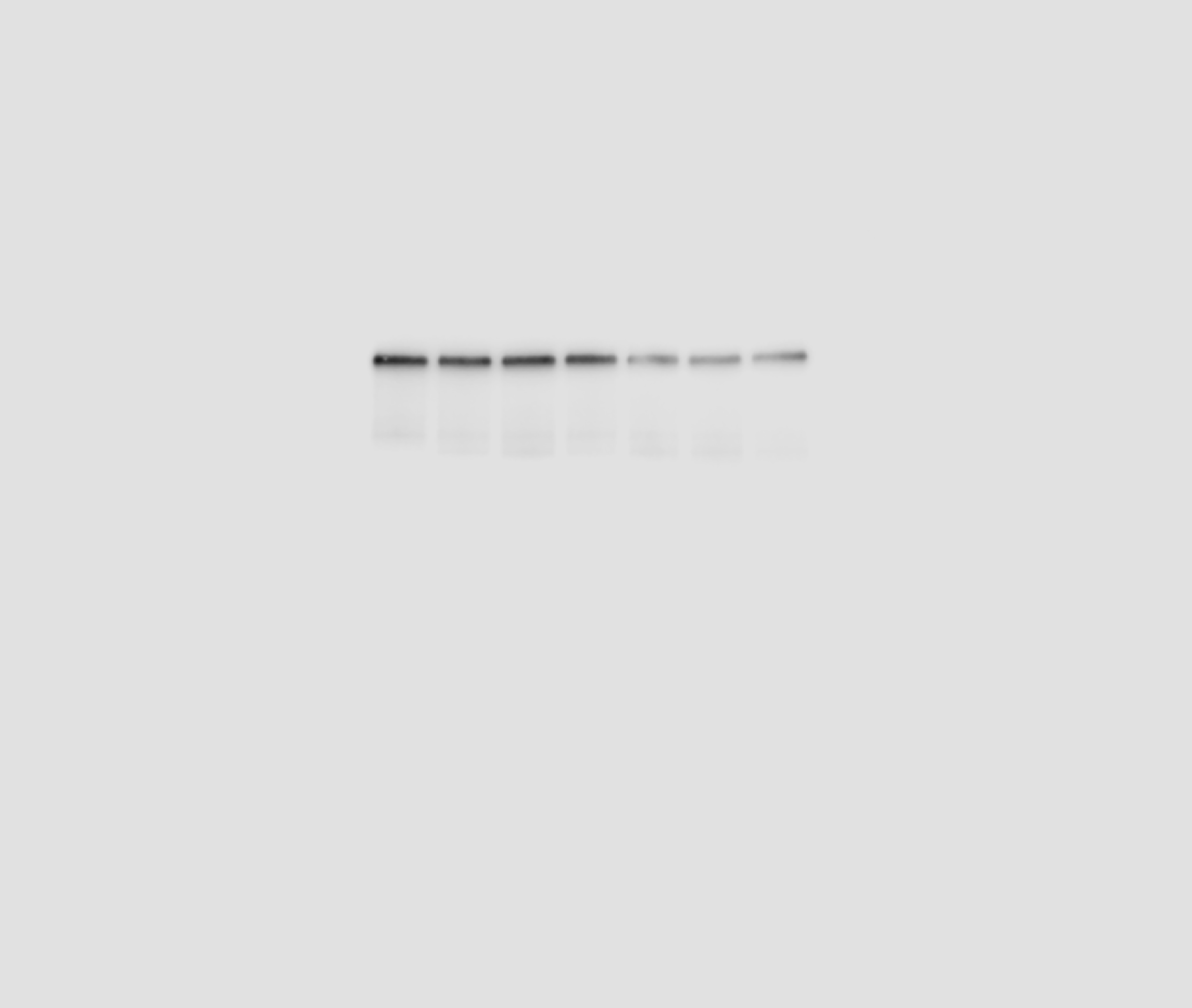

Supplement: Figure 3—source data 1. [file elife-83893-fig3-data1.zip › Figure 3-source data 1 /Figure 3-source data 1-raw files/Figure 3-source data 1-right panel-IP-FLAG channel.tif]

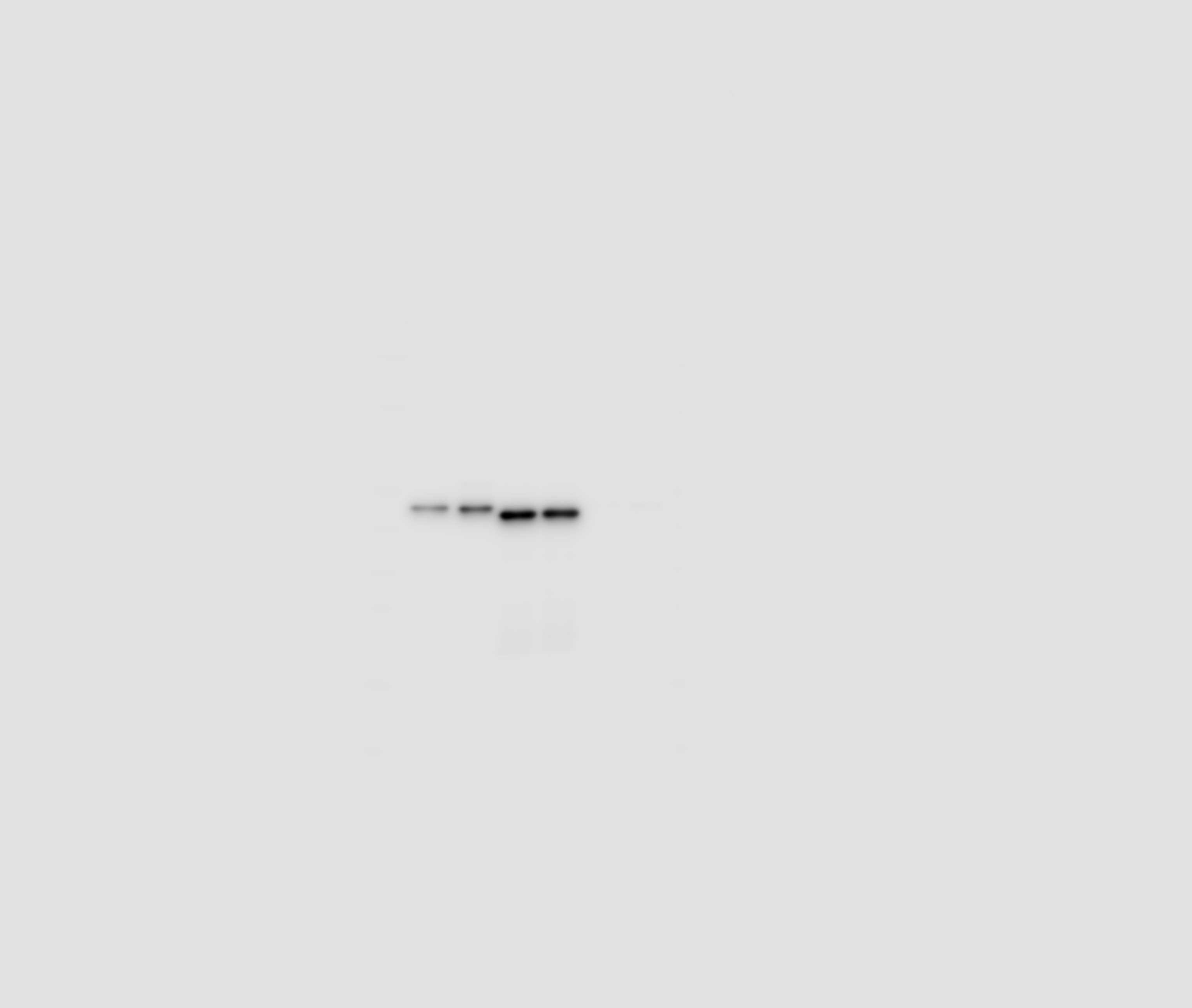

Supplement: Figure 3—source data 1. [file elife-83893-fig3-data1.zip › Figure 3-source data 1 /Figure 3-source data 1-raw files/Figure 3-source data 1-left panel-IP-GFP channel.tif]

Figure 4A

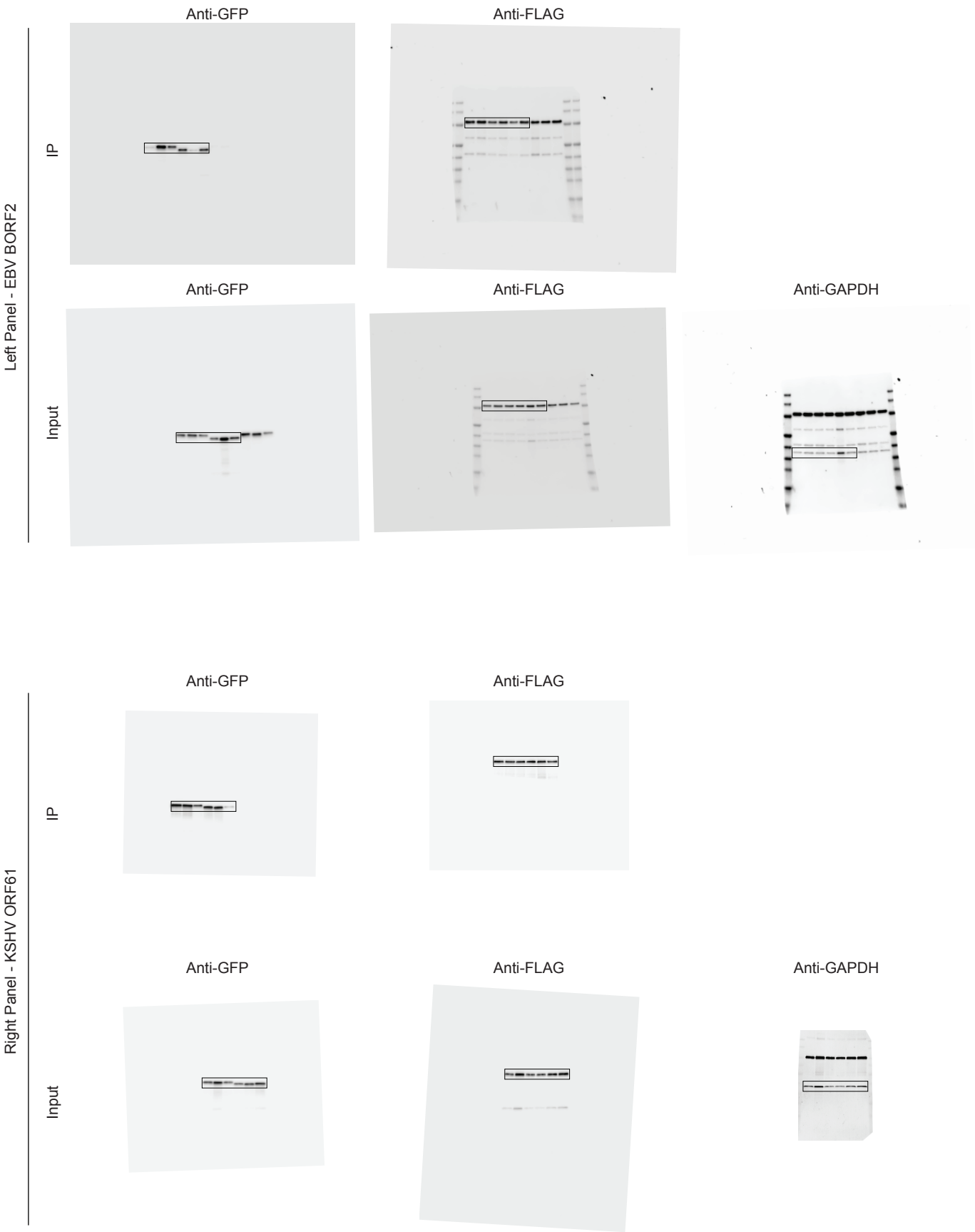

Supplement: Figure 4—source data 1. [file elife-83893-fig4-data1.zip › Figure 4-source data 1 /Figure 4-source data 1-uncropped.pdf]

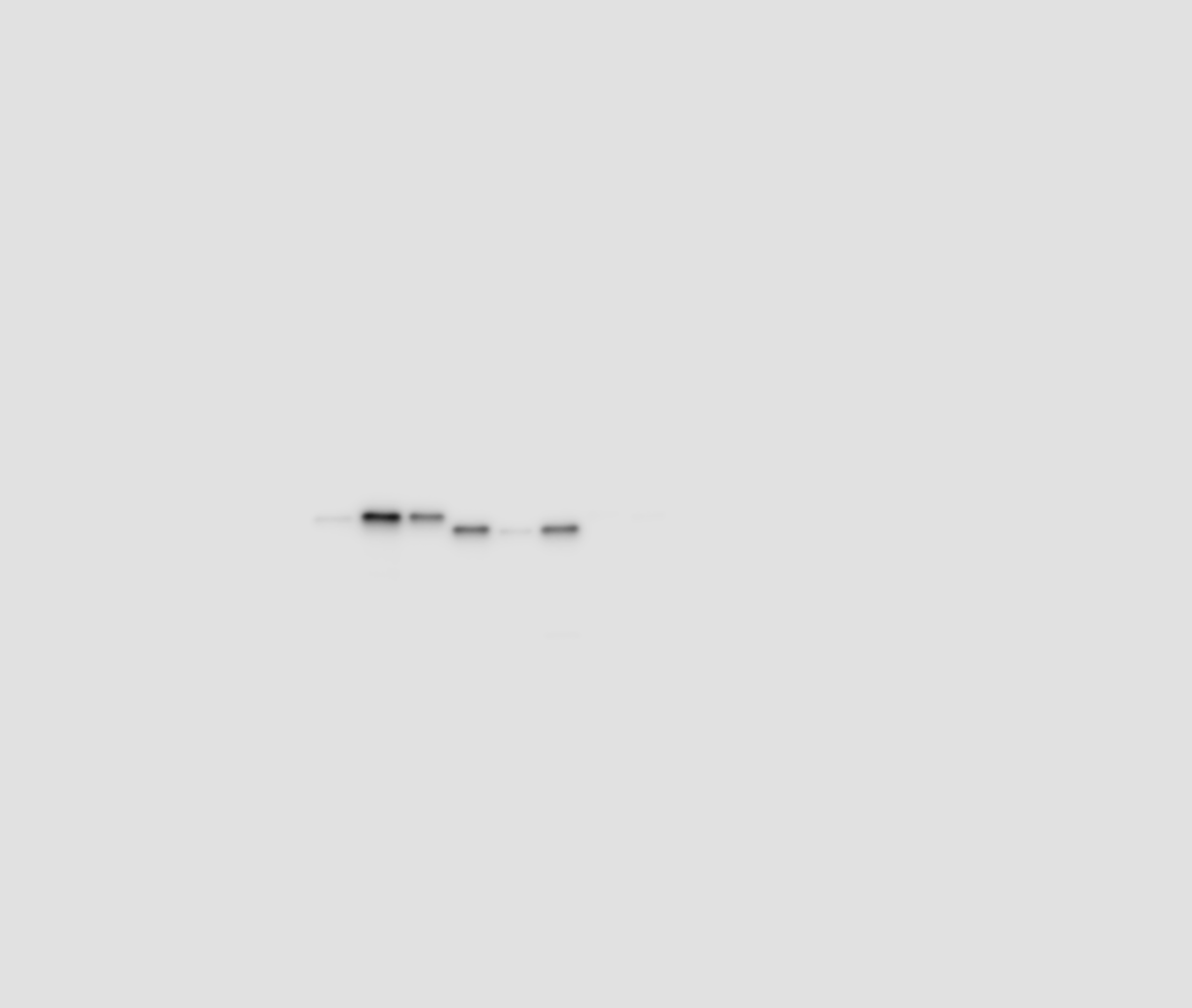

Supplement: Figure 4—source data 1. [file elife-83893-fig4-data1.zip › Figure 4-source data 1 /Figure 4-source data 1-raw files/Figure 4-source data 1-left panel-IP-GFP channel.tif]

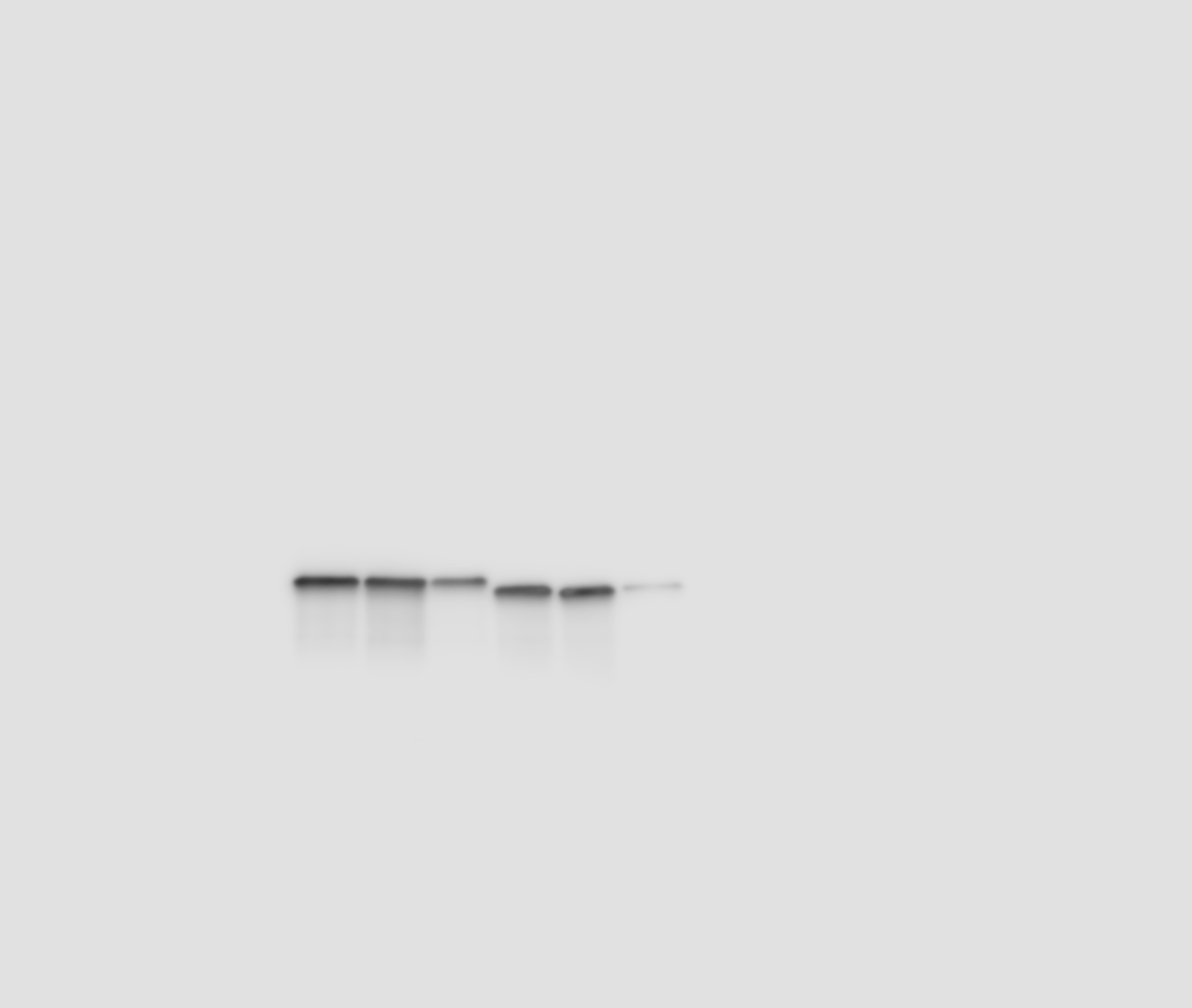

Supplement: Figure 4—source data 1. [file elife-83893-fig4-data1.zip › Figure 4-source data 1 /Figure 4-source data 1-raw files/Figure 4-source data 1-right panel-IP-GFP channel.tif]

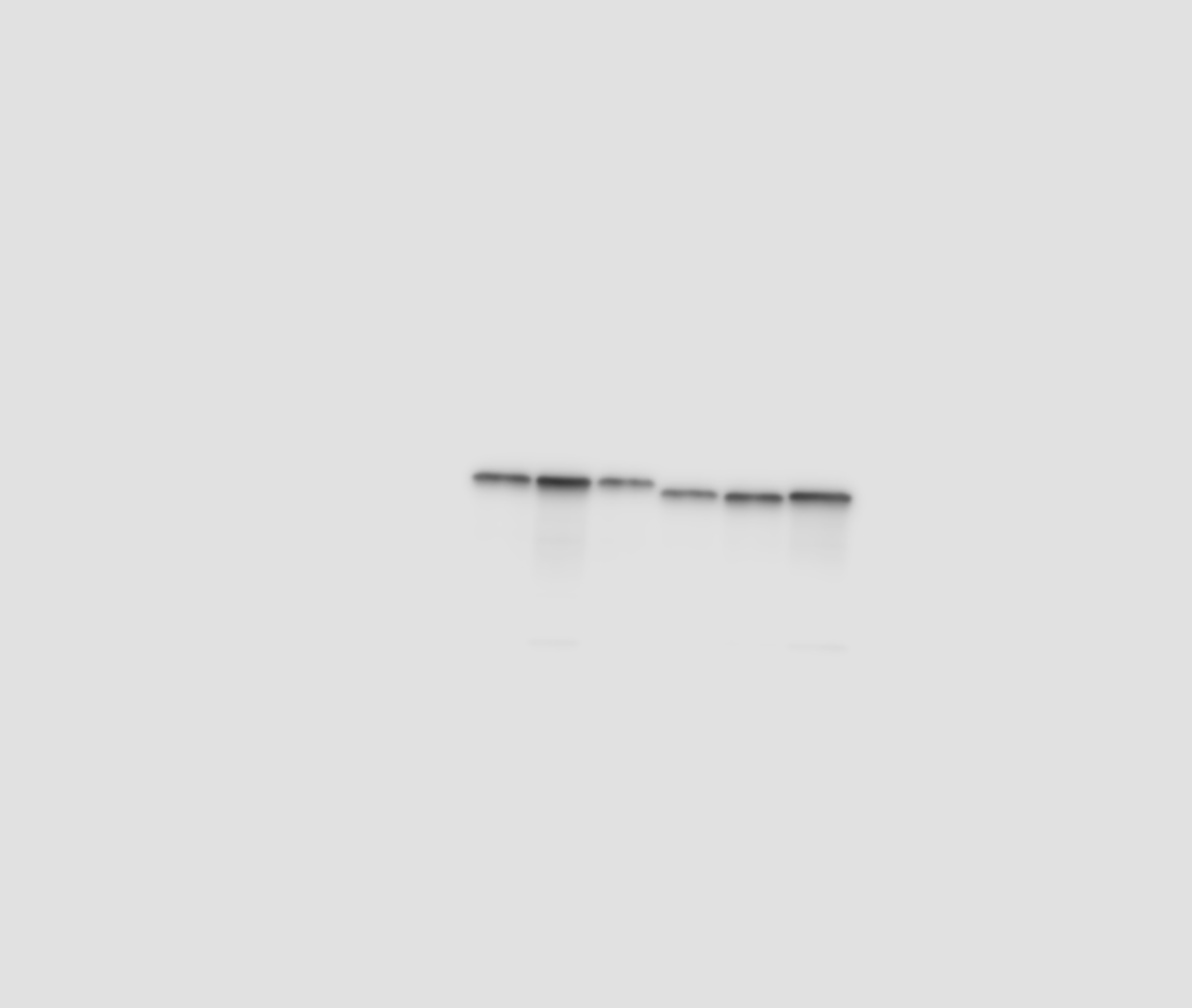

Supplement: Figure 4—source data 1. [file elife-83893-fig4-data1.zip › Figure 4-source data 1 /Figure 4-source data 1-raw files/Figure 4-source data 1-right panel-input-GFP channel.tif]

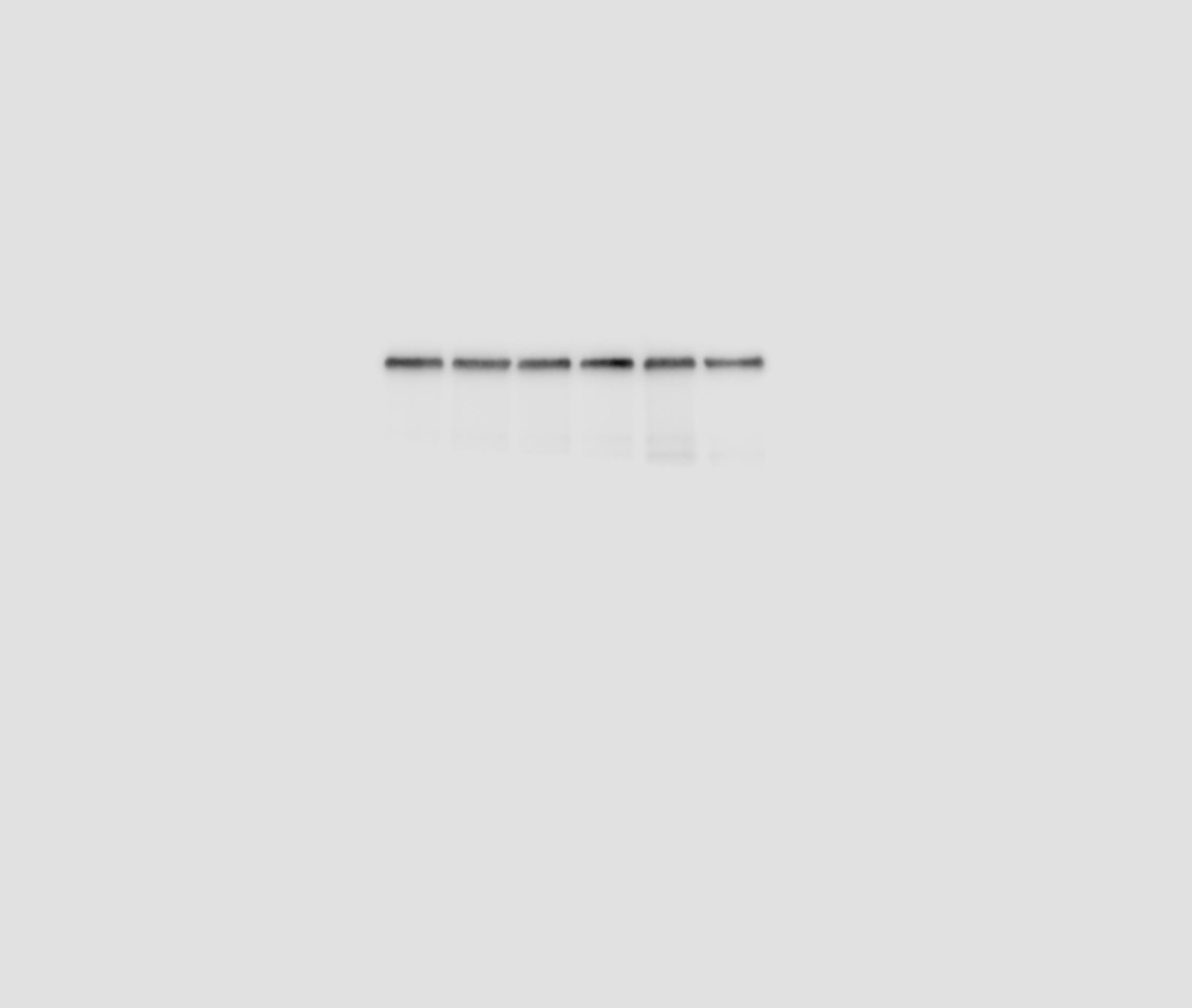

Supplement: Figure 4—source data 1. [file elife-83893-fig4-data1.zip › Figure 4-source data 1 /Figure 4-source data 1-raw files/Figure 4-source data 1-right panel-IP-FLAG channel.tif]

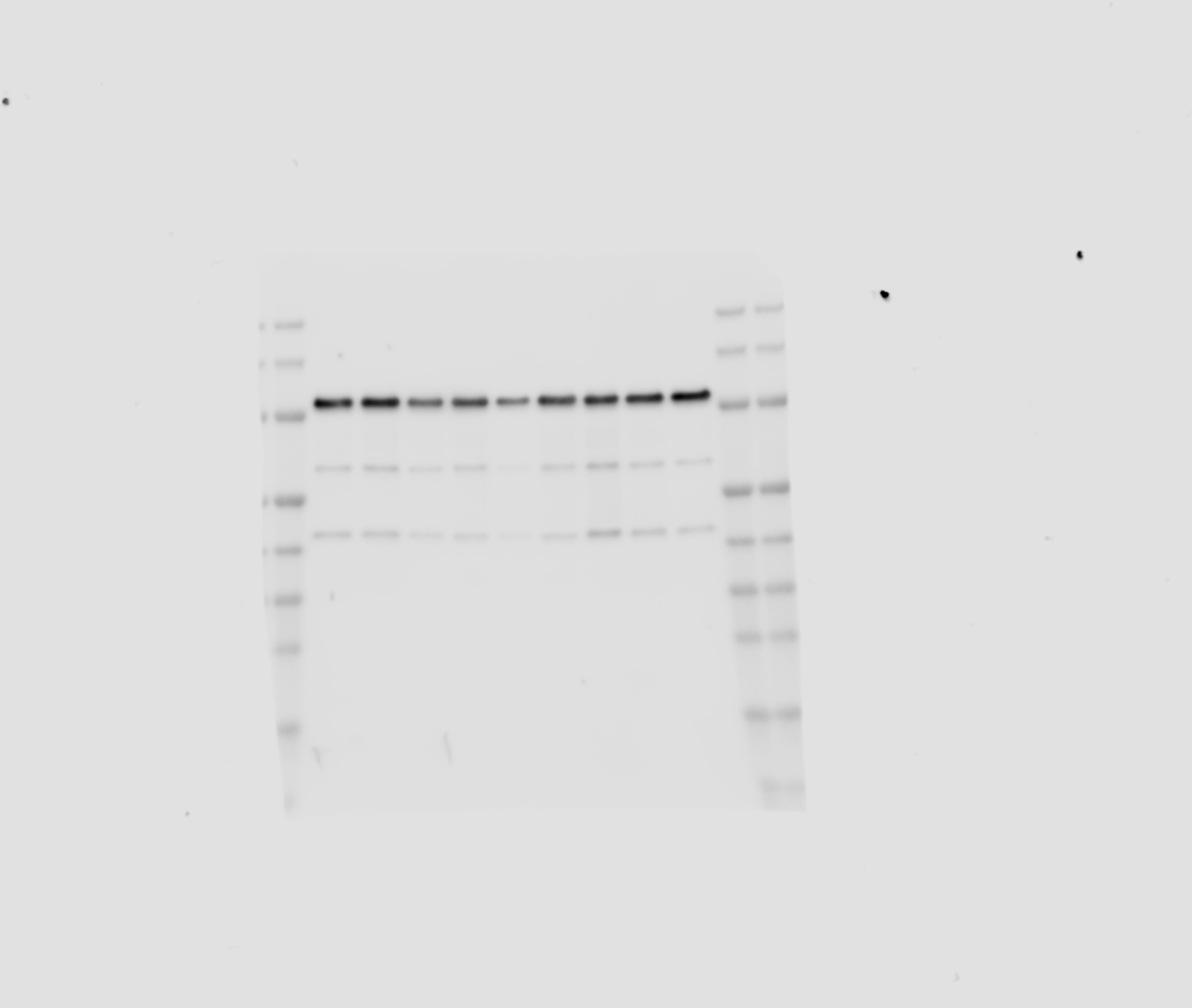

Supplement: Figure 4—source data 1. [file elife-83893-fig4-data1.zip › Figure 4-source data 1 /Figure 4-source data 1-raw files/Figure 4-source data 1-left panel-IP-FLAG channel.tif]

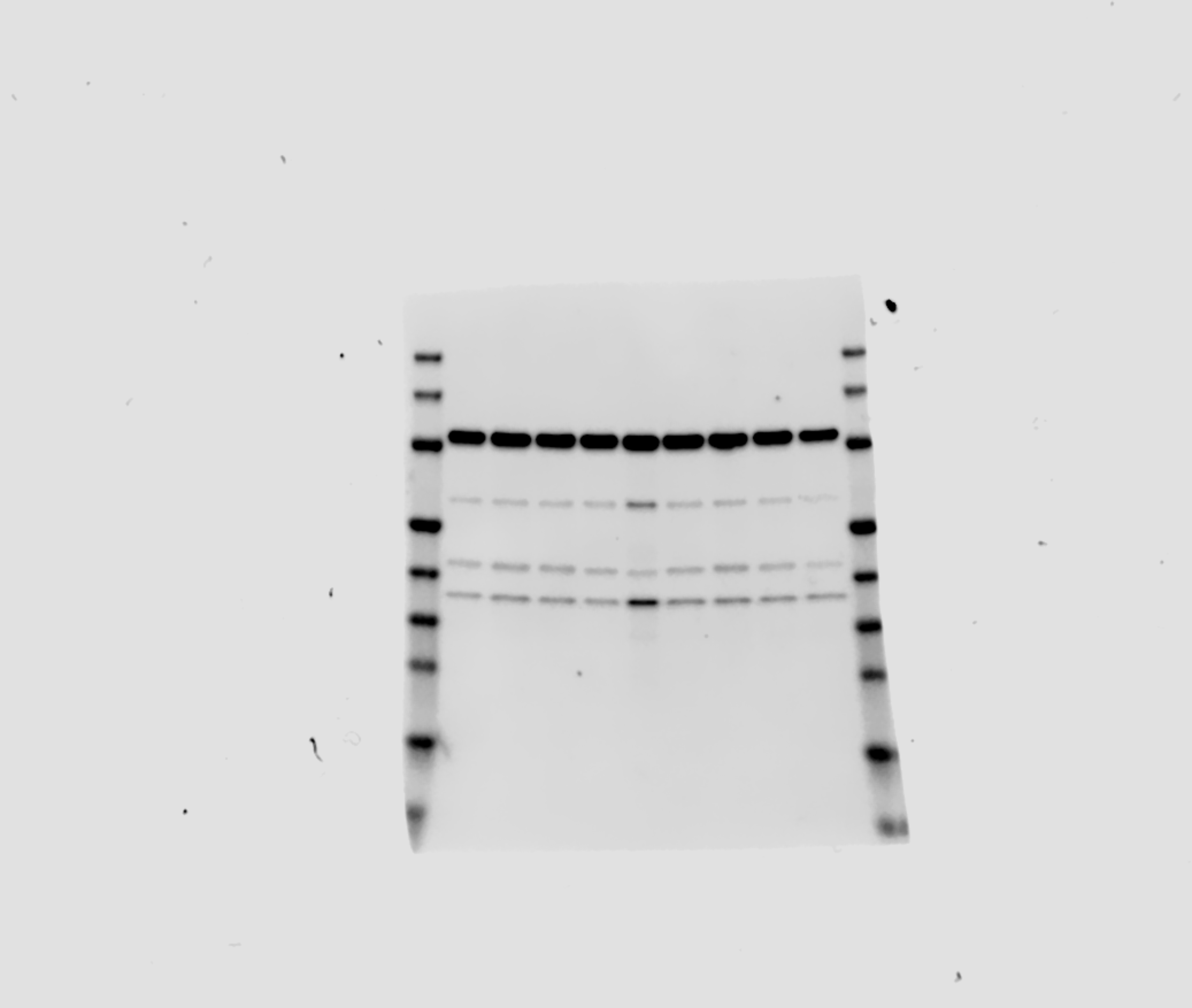

Supplement: Figure 4—source data 1. [file elife-83893-fig4-data1.zip › Figure 4-source data 1 /Figure 4-source data 1-raw files/Figure 4-source data 1-left panel-input- GAPDH channel.tif]

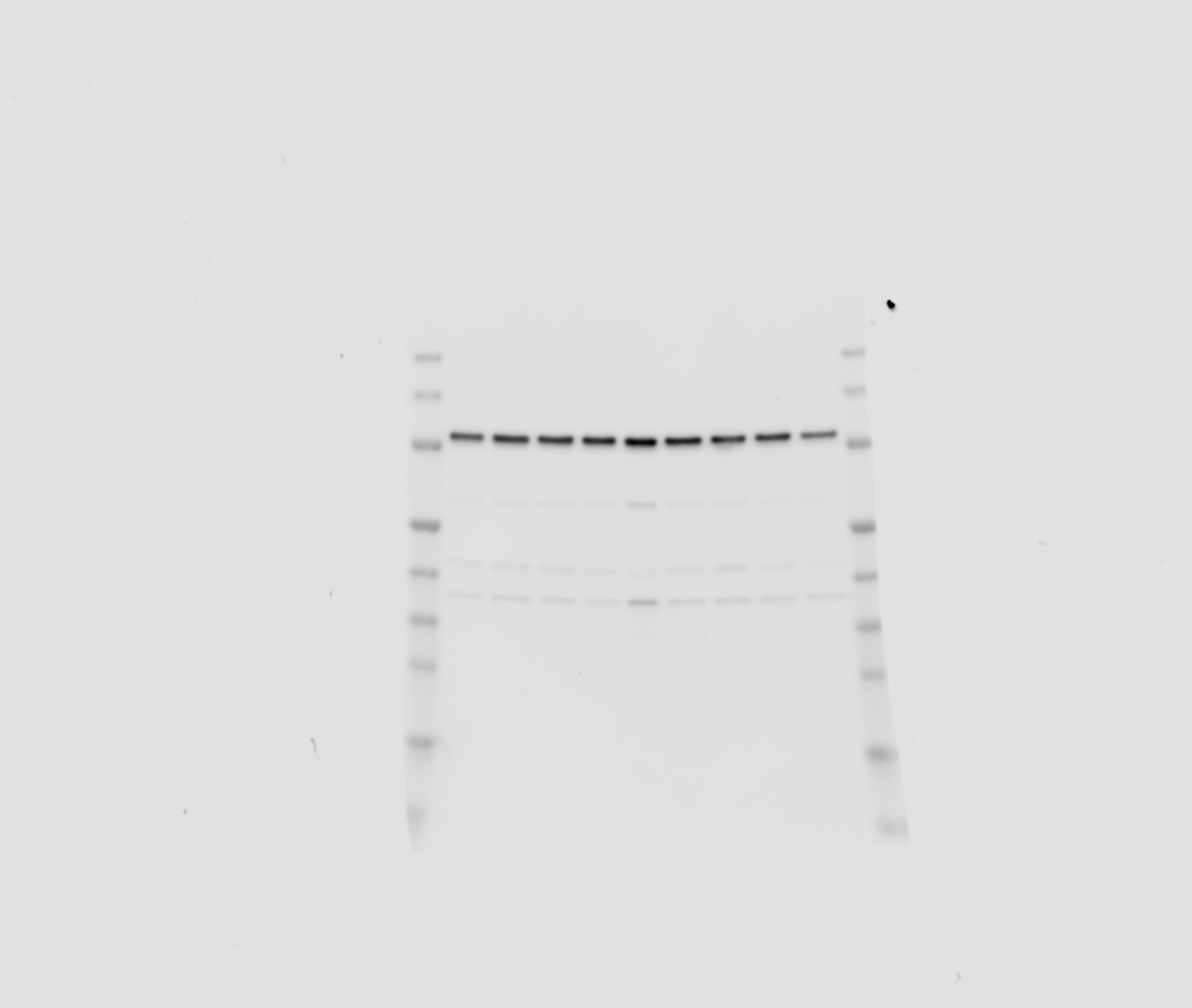

Supplement: Figure 4—source data 1. [file elife-83893-fig4-data1.zip › Figure 4-source data 1 /Figure 4-source data 1-raw files/Figure 4-source data 1-left panel-input-FLAG channel.tif]

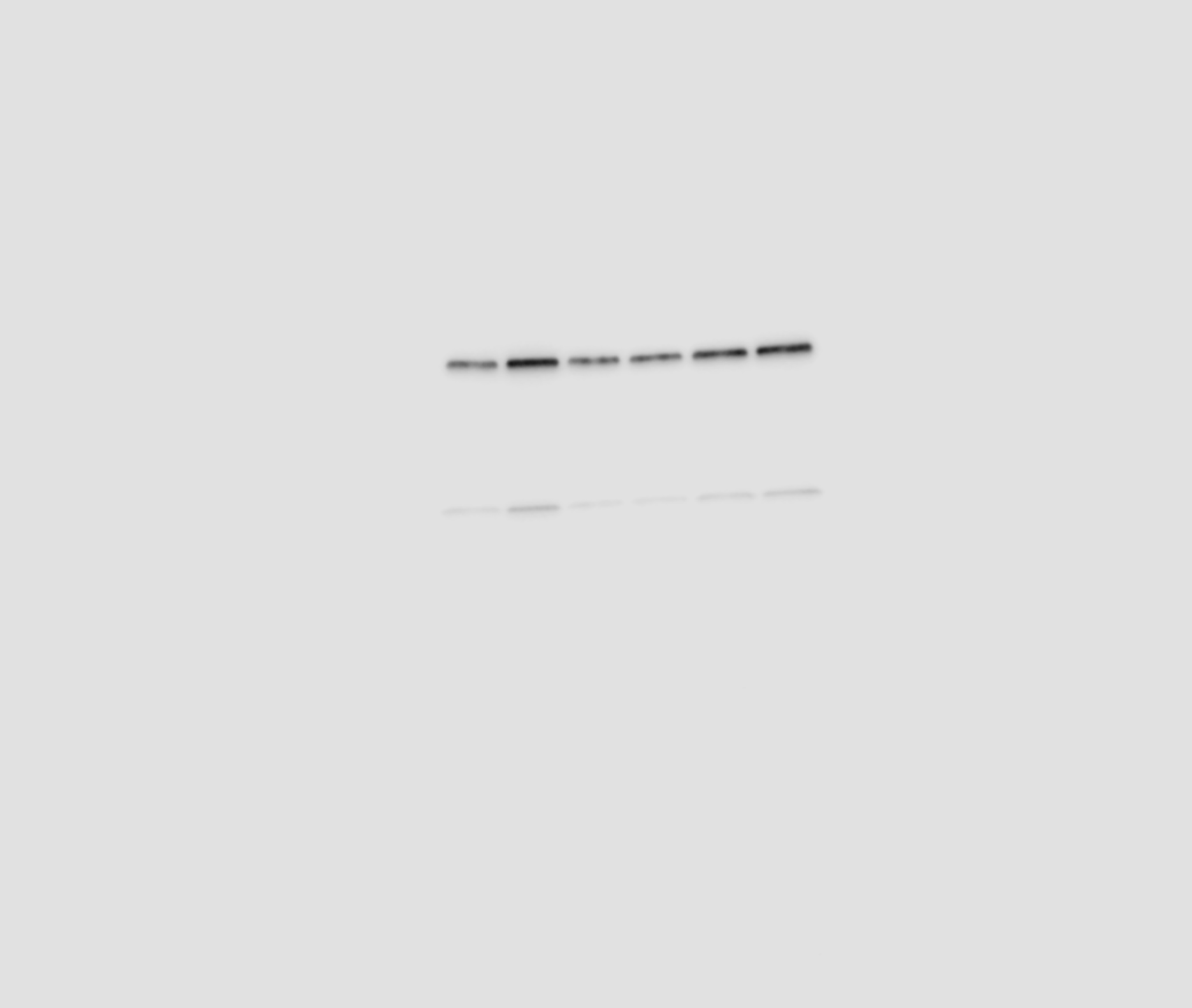

Supplement: Figure 4—source data 1. [file elife-83893-fig4-data1.zip › Figure 4-source data 1 /Figure 4-source data 1-raw files/Figure 4-source data 1-right panel-input-FLAG channel.tif]

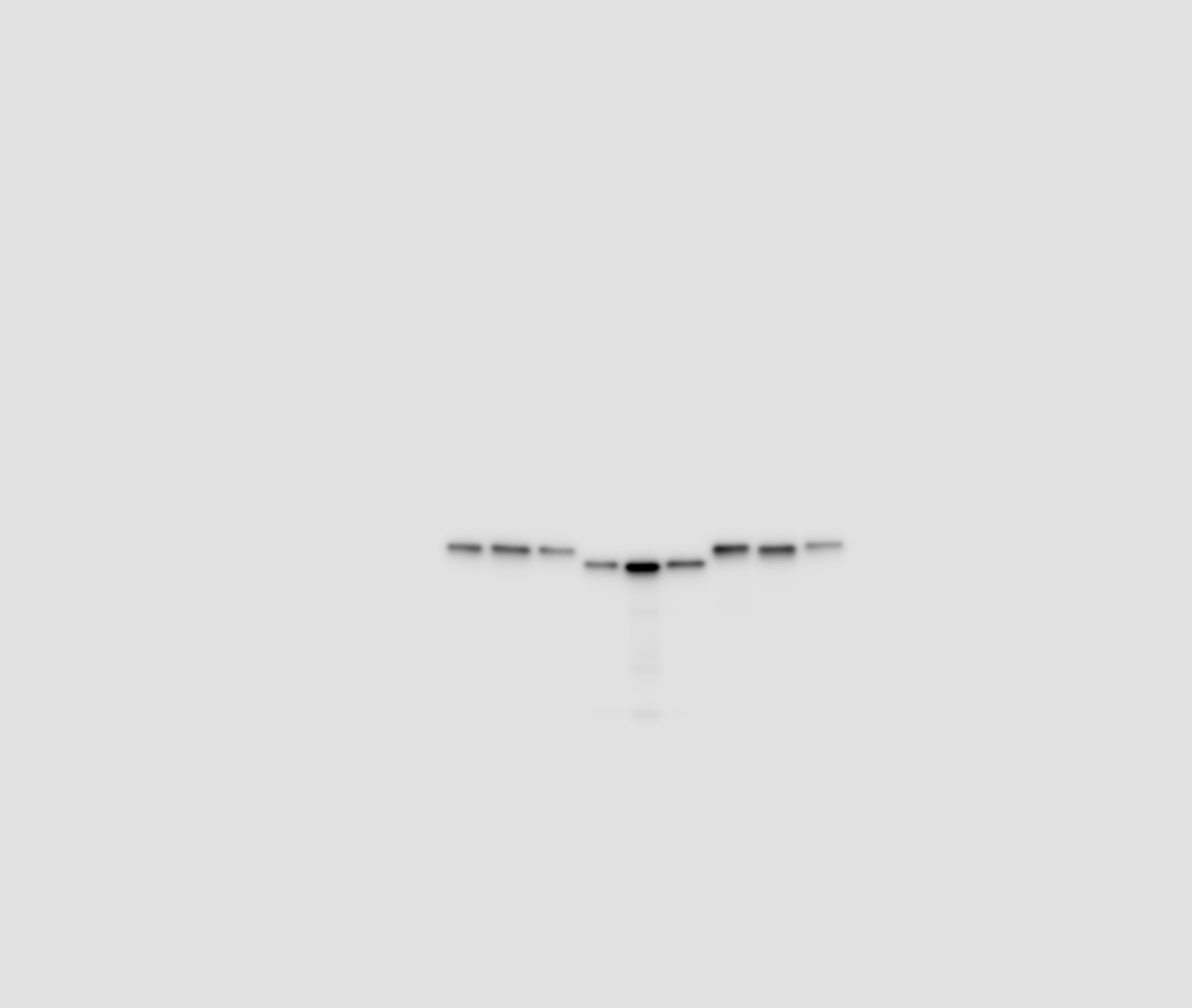

Supplement: Figure 4—source data 1. [file elife-83893-fig4-data1.zip › Figure 4-source data 1 /Figure 4-source data 1-raw files/Figure 4-source data 1-left panel-input-GFP channel.tif]

Figure 5A

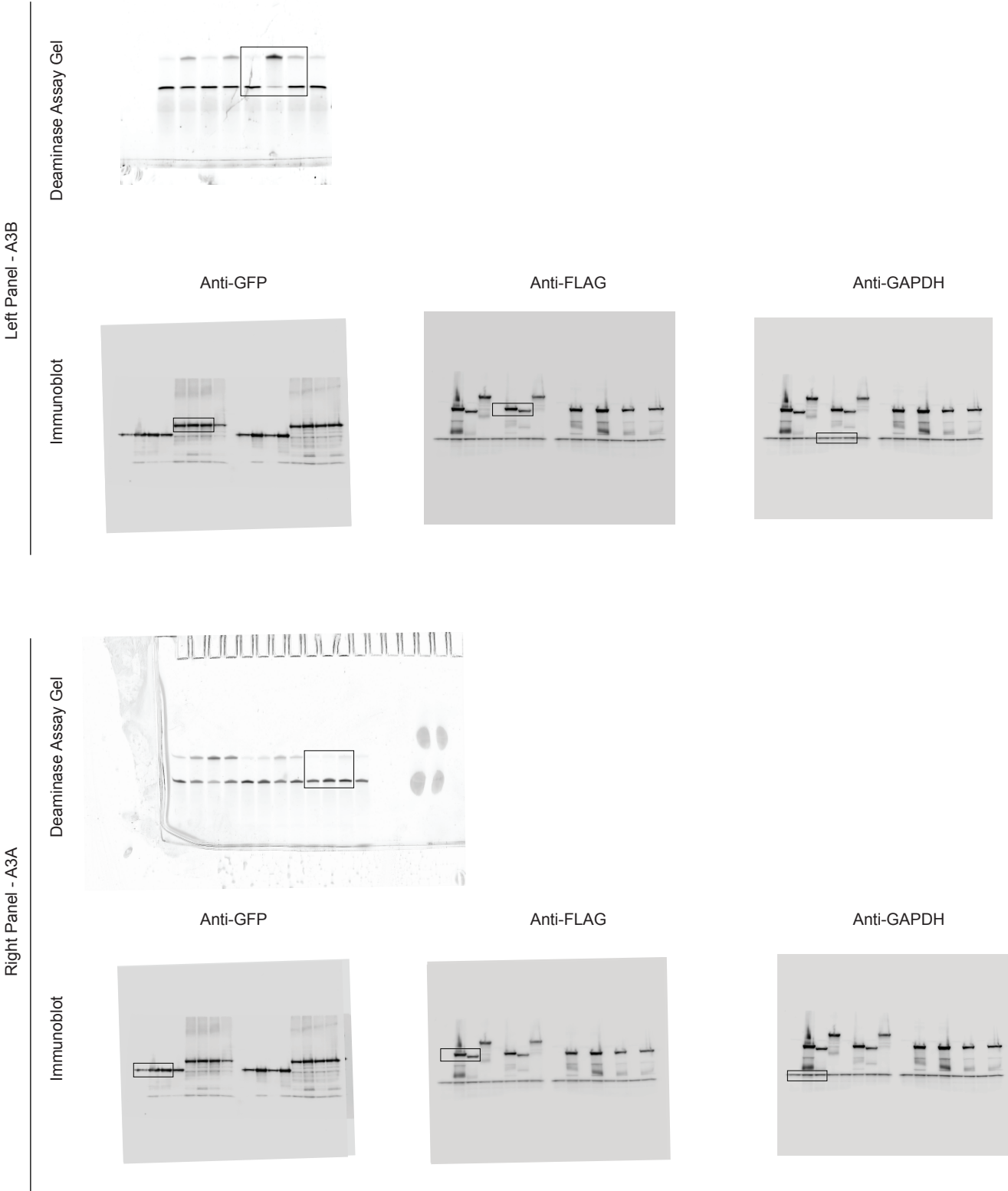

Supplement: Figure 5—source data 1. [file elife-83893-fig5-data1.zip › Figure 5-source data 1 /Figure 5-source data 1-uncropped.pdf]

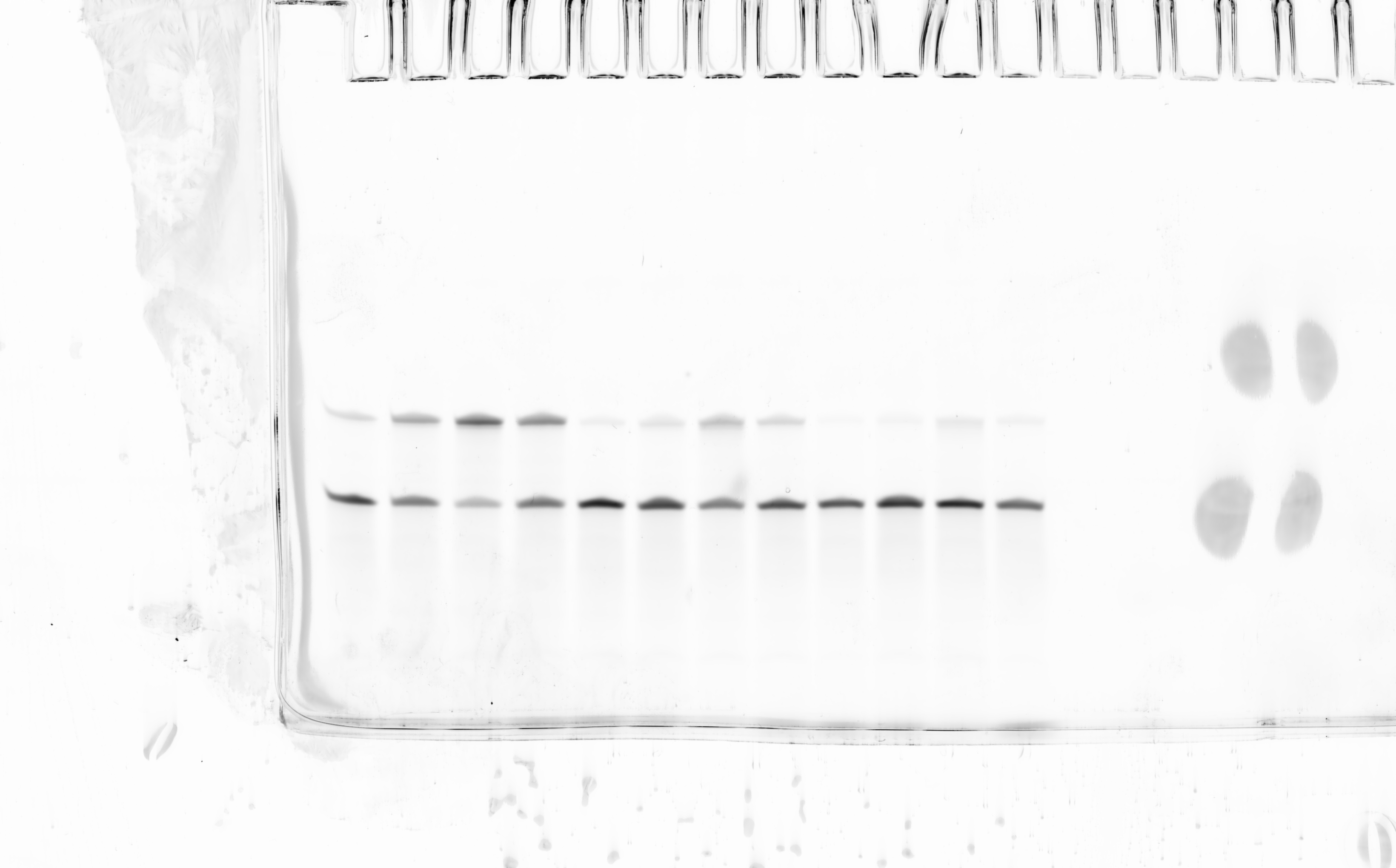

Supplement: Figure 5—source data 1. [file elife-83893-fig5-data1.zip › Figure 5-source data 1 /Figure 5-source data 1-raw files/Figure 5-source data 1-right panel-deaminase assay gel.tif]

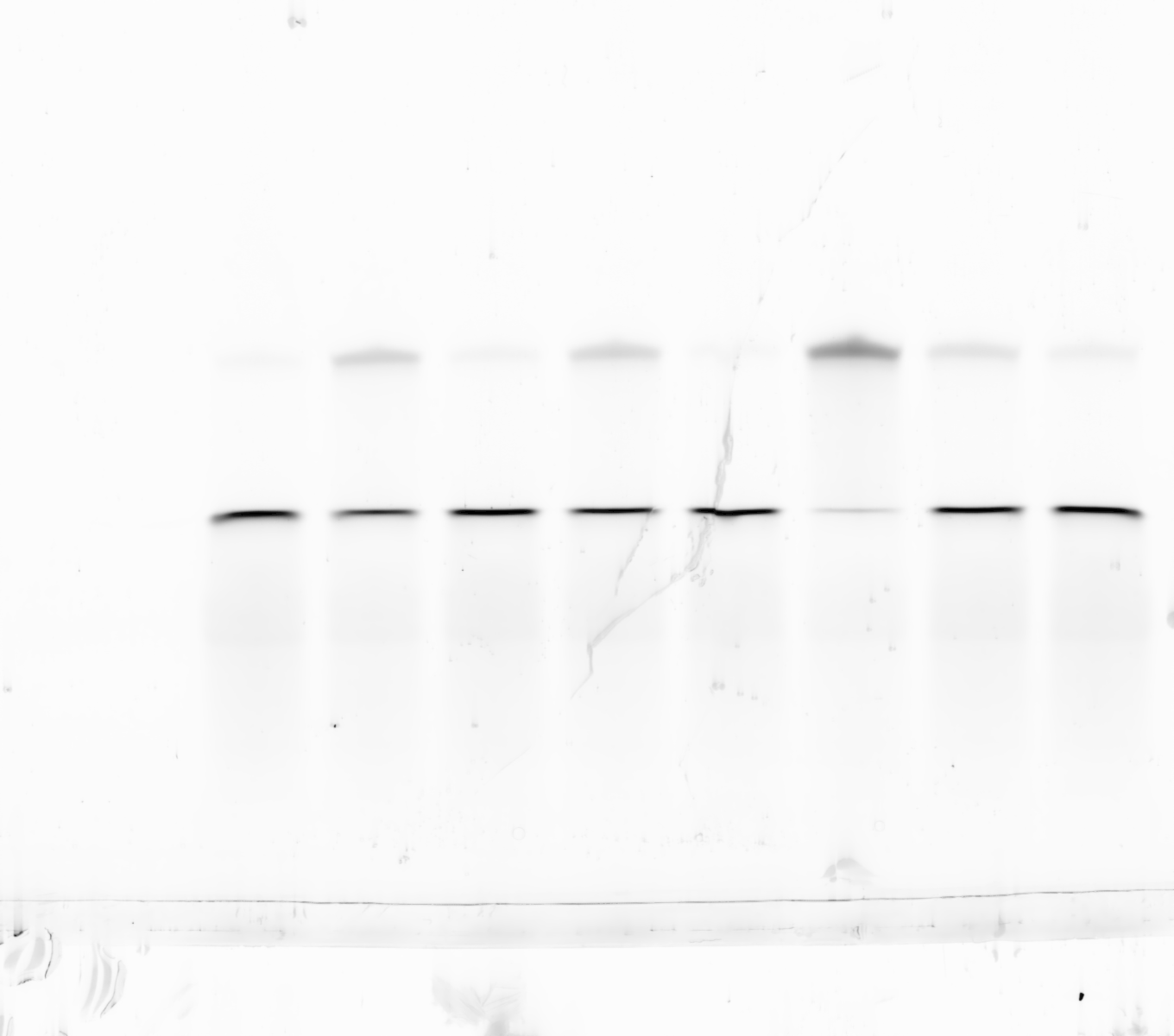

Supplement: Figure 5—source data 1. [file elife-83893-fig5-data1.zip › Figure 5-source data 1 /Figure 5-source data 1-raw files/Figure 5-source data 1-left panel-deaminase assay gel.tif]

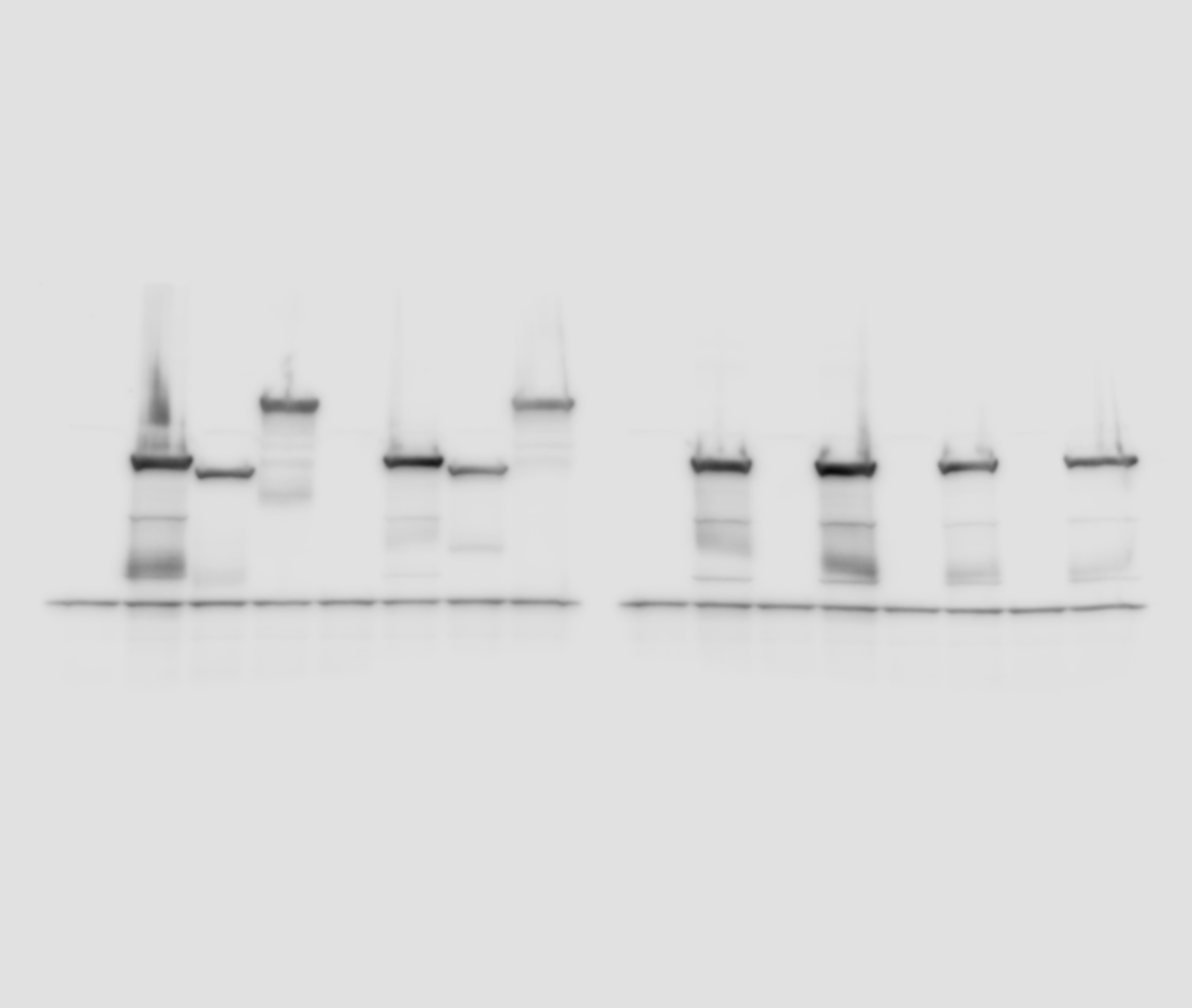

Supplement: Figure 5—source data 1. [file elife-83893-fig5-data1.zip › Figure 5-source data 1 /Figure 5-source data 1-raw files/Figure 5-source data 1-immunoblot-FLAG and GAPDH channel.tif]

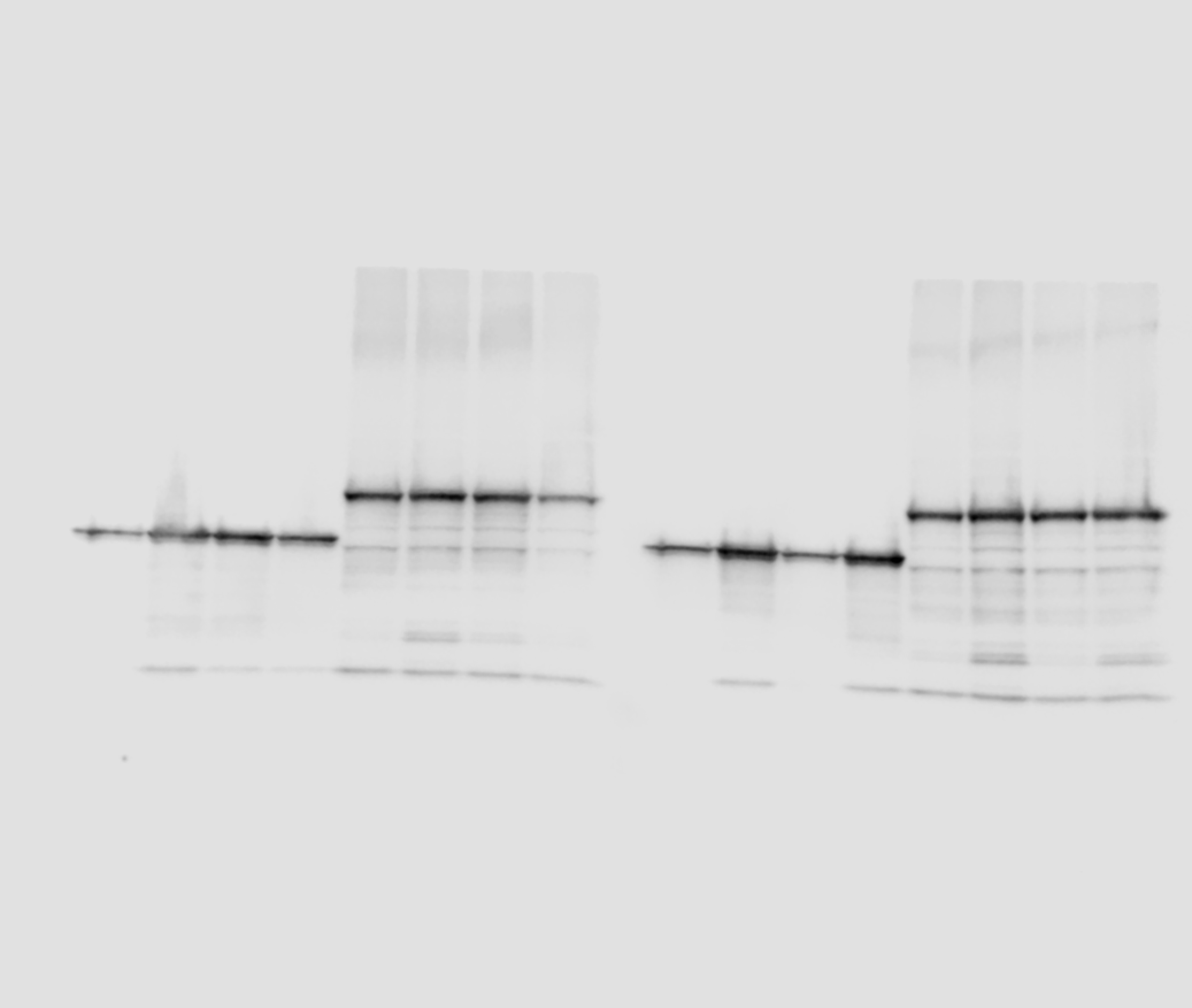

Supplement: Figure 5—source data 1. [file elife-83893-fig5-data1.zip › Figure 5-source data 1 /Figure 5-source data 1-raw files/Figure 5-source data 1-immunoblot-GFP channel.tif]

Figure 5C

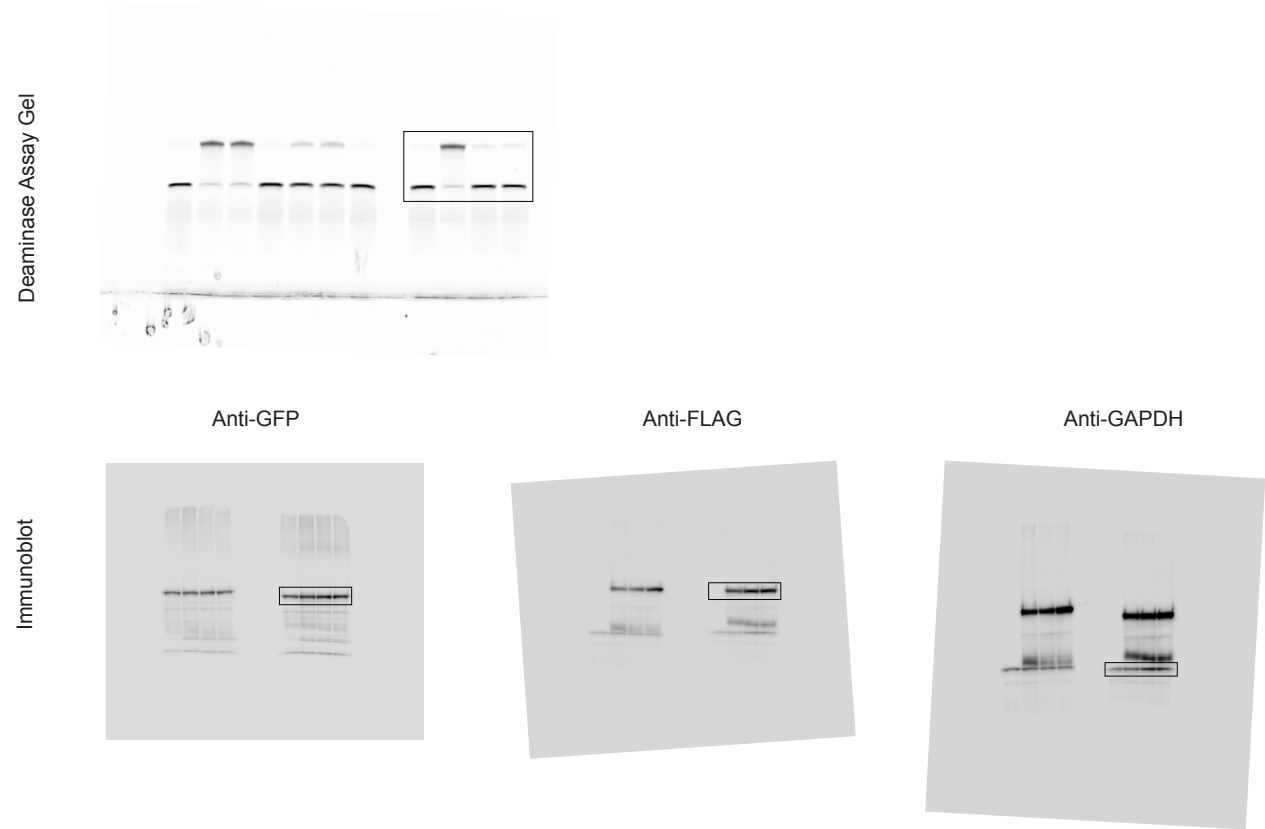

Supplement: Figure 5—source data 2. [file elife-83893-fig5-data2.zip › Figure 5-source data 2/Figure 5-source data 2-uncropped.pdf]

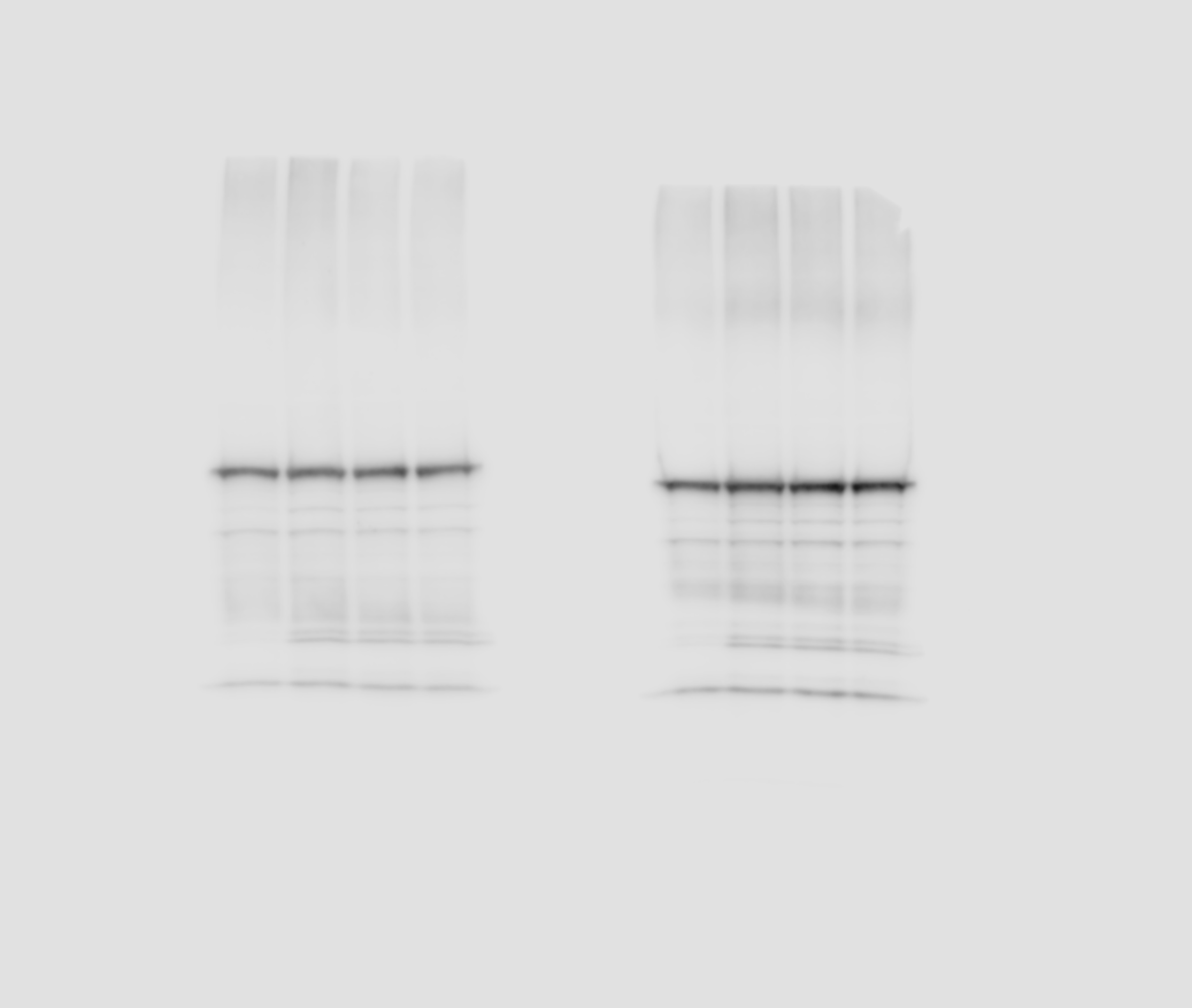

Supplement: Figure 5—source data 2. [file elife-83893-fig5-data2.zip › Figure 5-source data 2/Figure 5-source data 2-raw files/Figure 5-source data 2-immunoblot-GFP channel.tif]

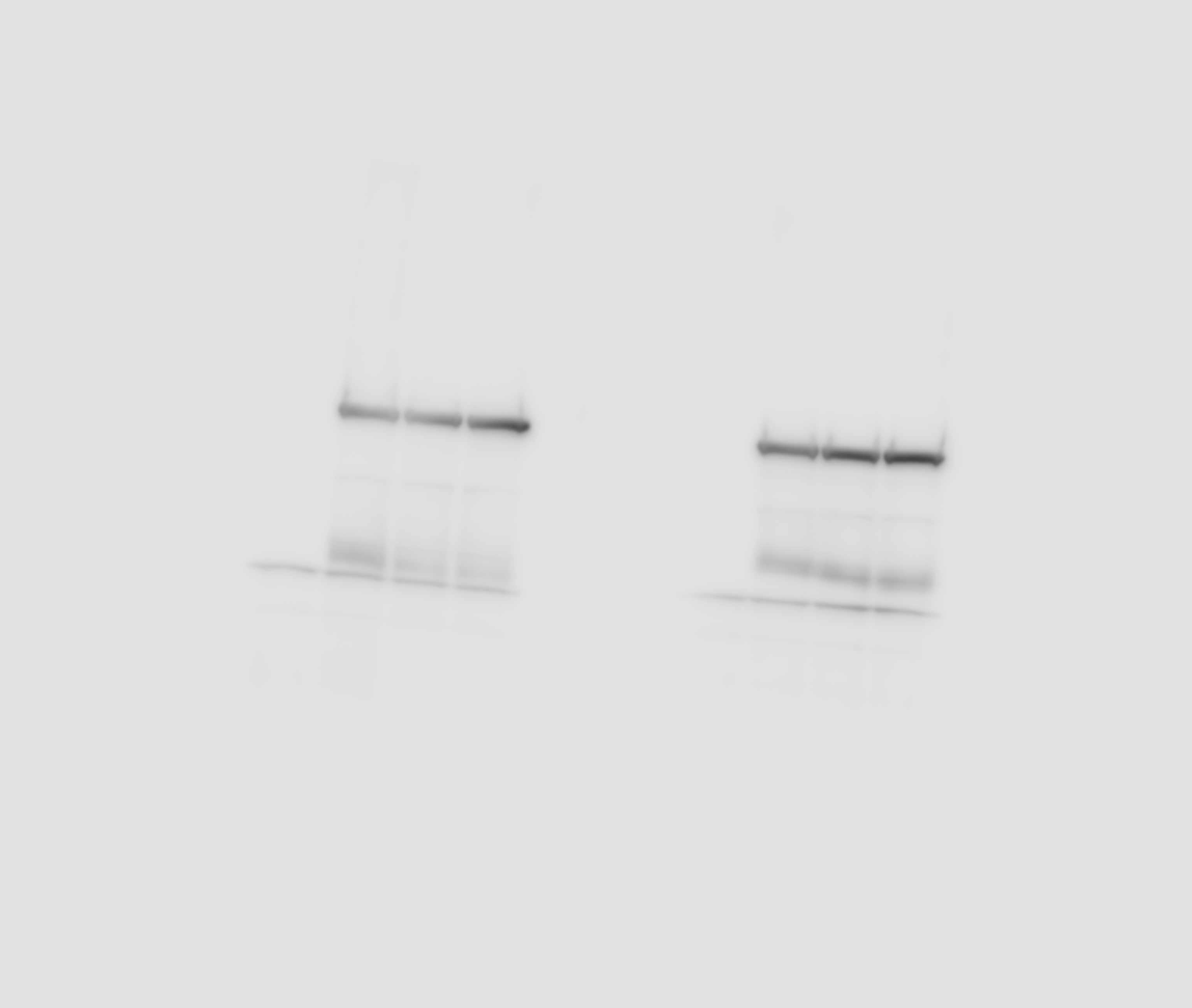

Supplement: Figure 5—source data 2. [file elife-83893-fig5-data2.zip › Figure 5-source data 2/Figure 5-source data 2-raw files/Figure 5-source data 2-immunoblot-FLAG channel.tif]

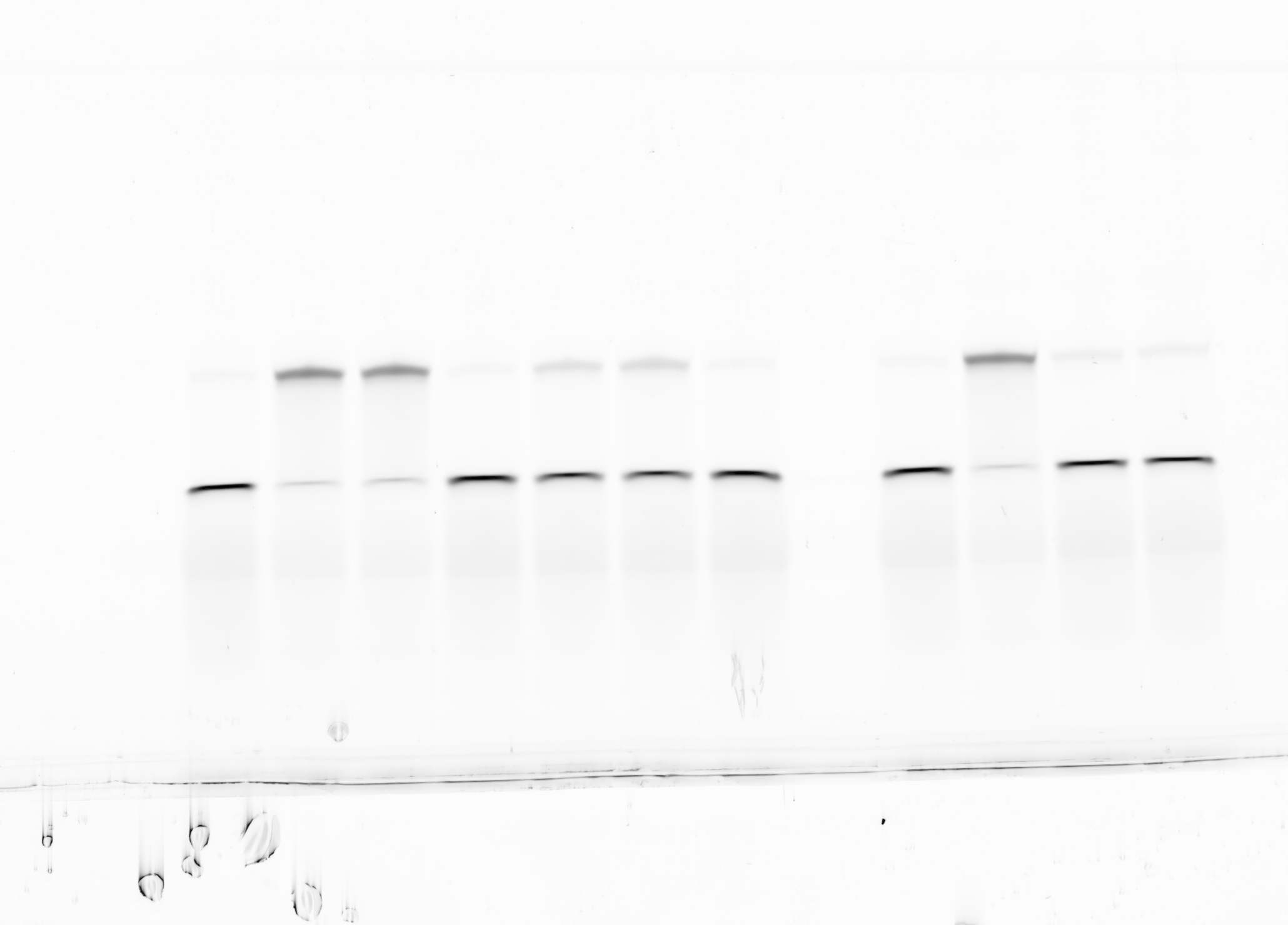

Supplement: Figure 5—source data 2. [file elife-83893-fig5-data2.zip › Figure 5-source data 2/Figure 5-source data 2-raw files/Figure 5-source data 2-deaminase assay gel.tif]

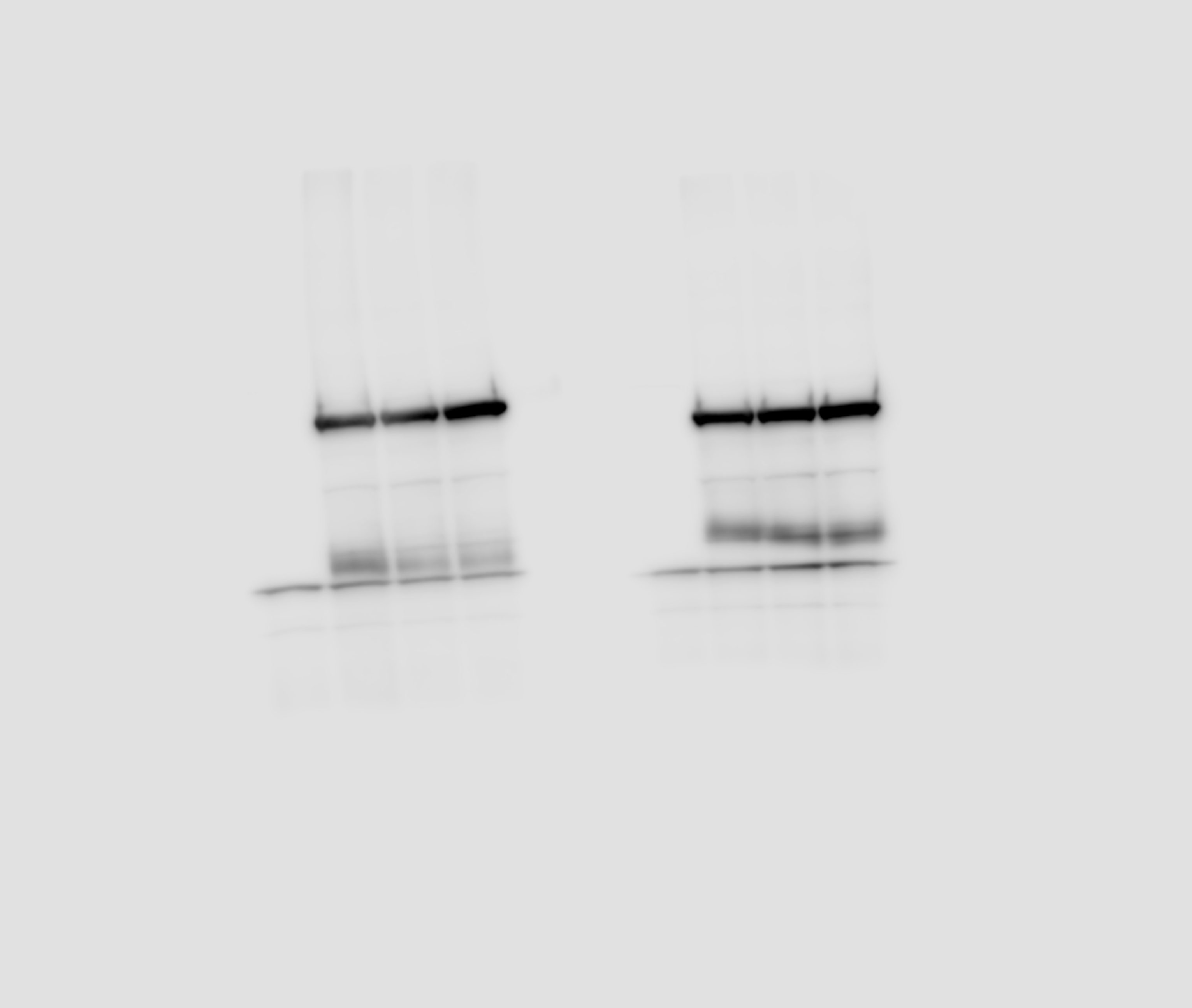

Supplement: Figure 5—source data 2. [file elife-83893-fig5-data2.zip › Figure 5-source data 2/Figure 5-source data 2-raw files/Figure 5-source data 2-immunoblot-GAPDH channel.tif.tif]

Figure 5D

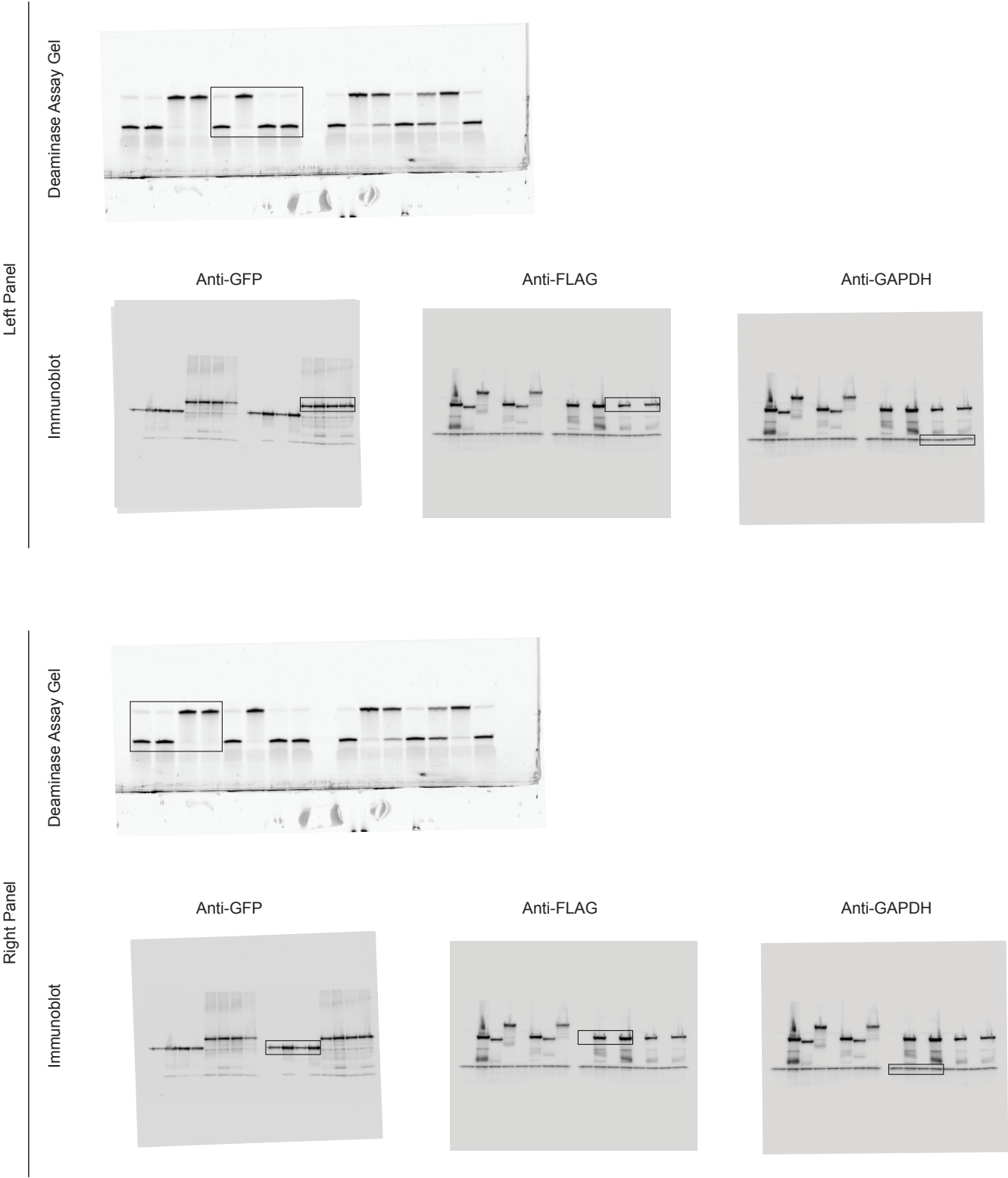

Supplement: Figure 5—source data 3. [file elife-83893-fig5-data3.zip › Figure 5-source data 3/Figure 5-source data 3-uncropped.pdf]

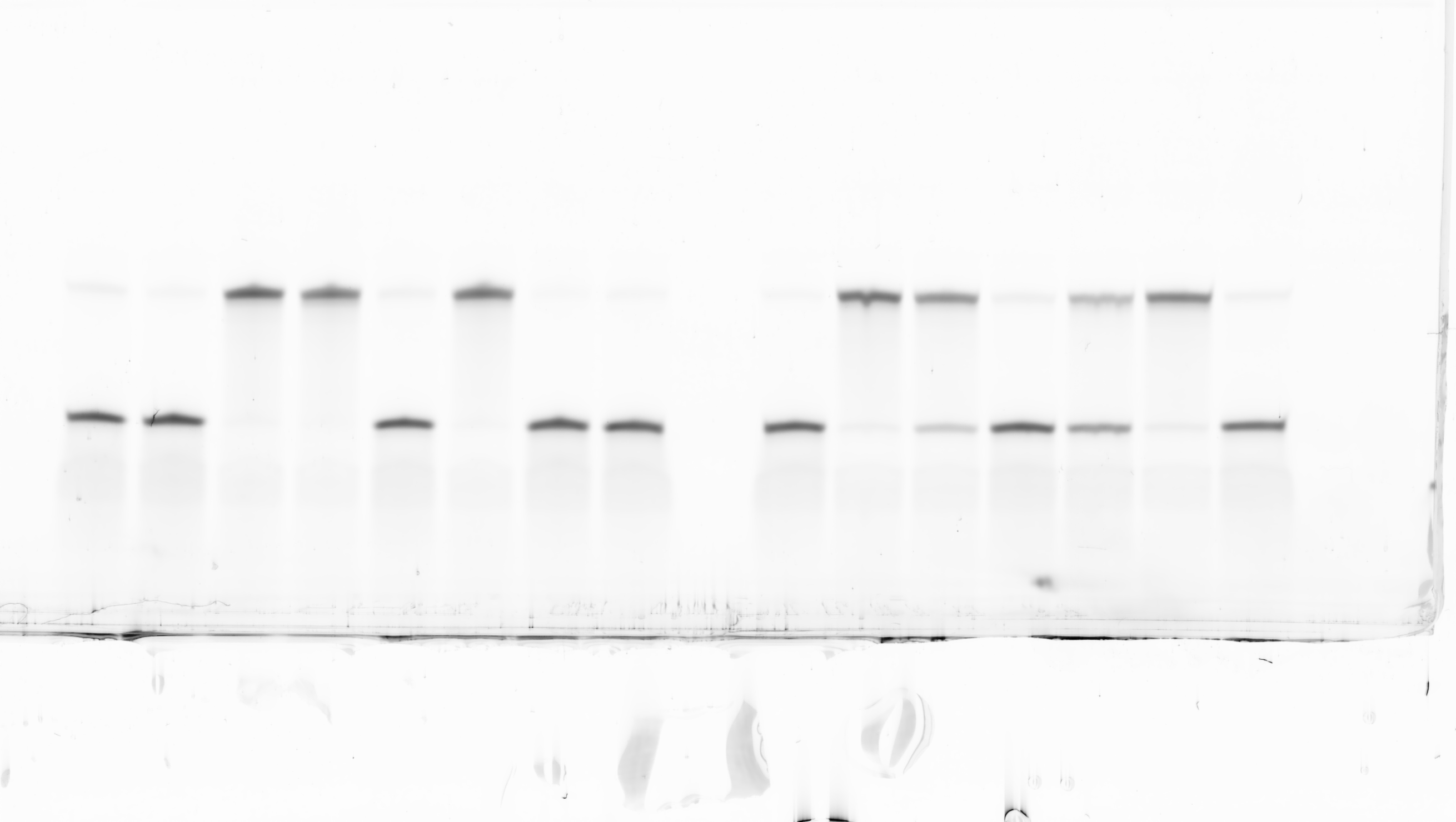

Supplement: Figure 5—source data 3. [file elife-83893-fig5-data3.zip › Figure 5-source data 3/Figure 5-source data 2-raw files/Figure 5-source data 3-deaminase assay gel.tif]

Figure 6C

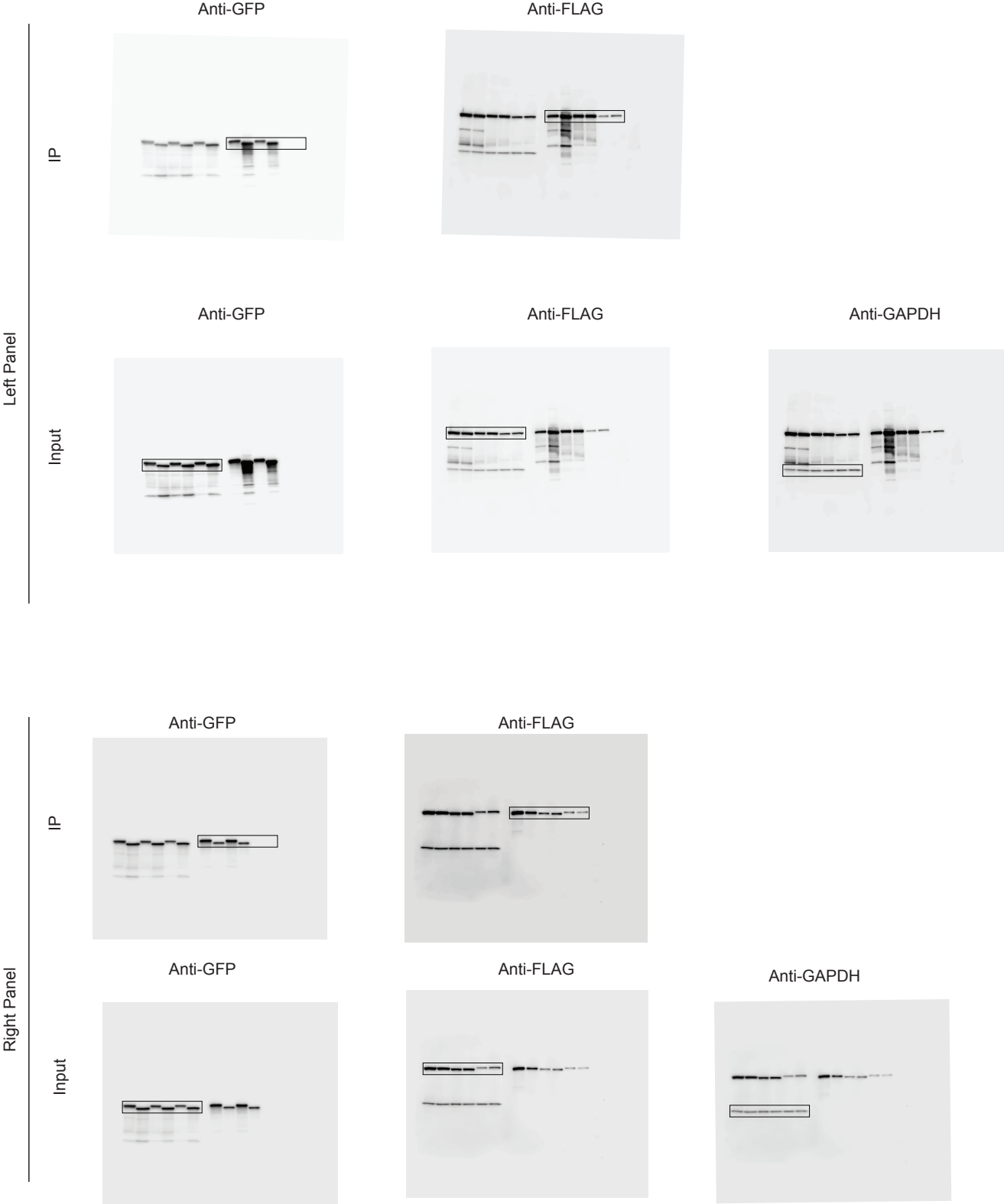

Supplement: Figure 6—source data 1. [file elife-83893-fig6-data1.zip › Figure 6-source data 1/Figure 6-source data 1-uncropped.pdf]

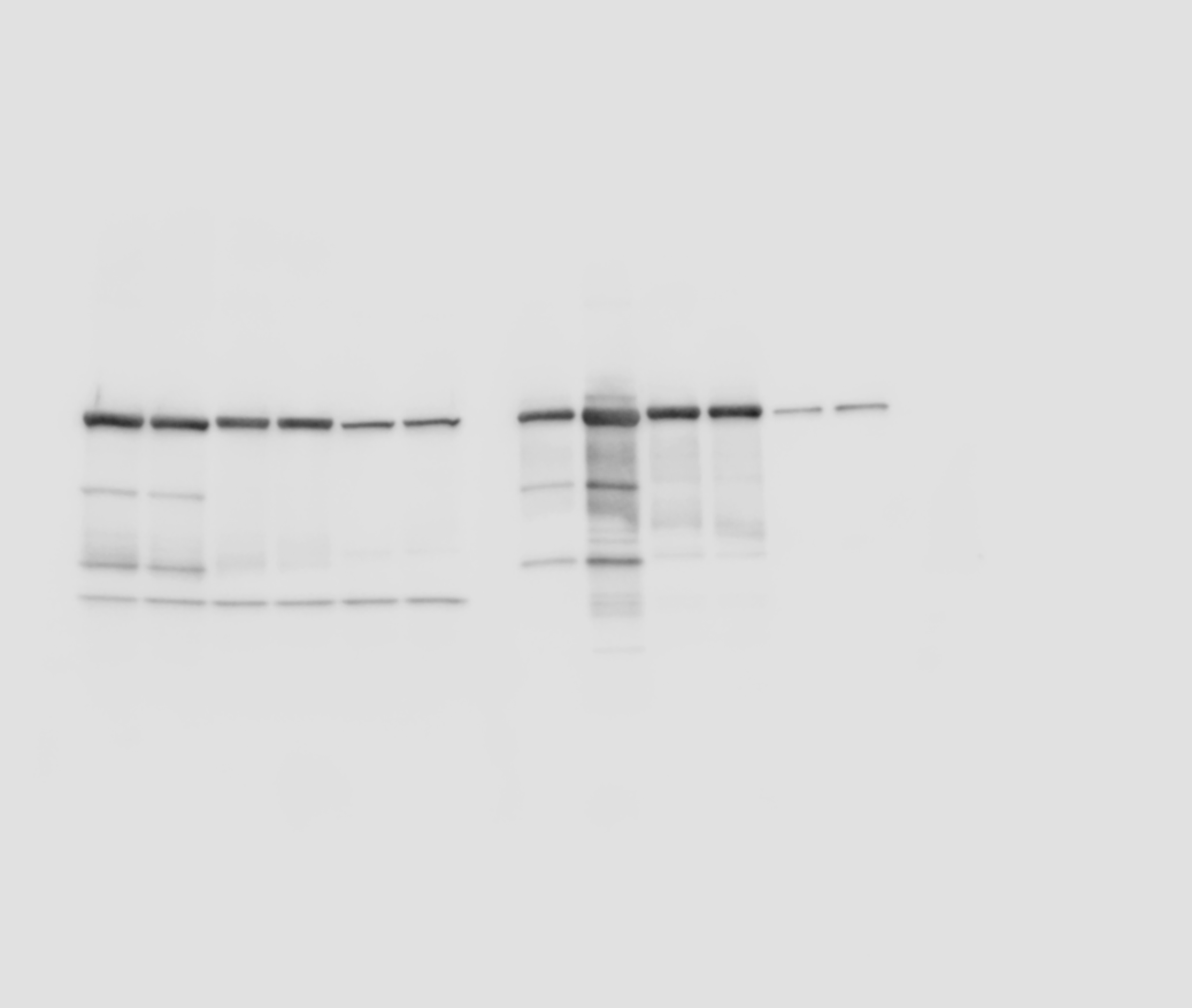

Supplement: Figure 6—source data 1. [file elife-83893-fig6-data1.zip › Figure 6-source data 1/Figure 6-source data 1-raw files/Figure 6-source data 1-left panel-FLAG and GAPDH channel.tif]

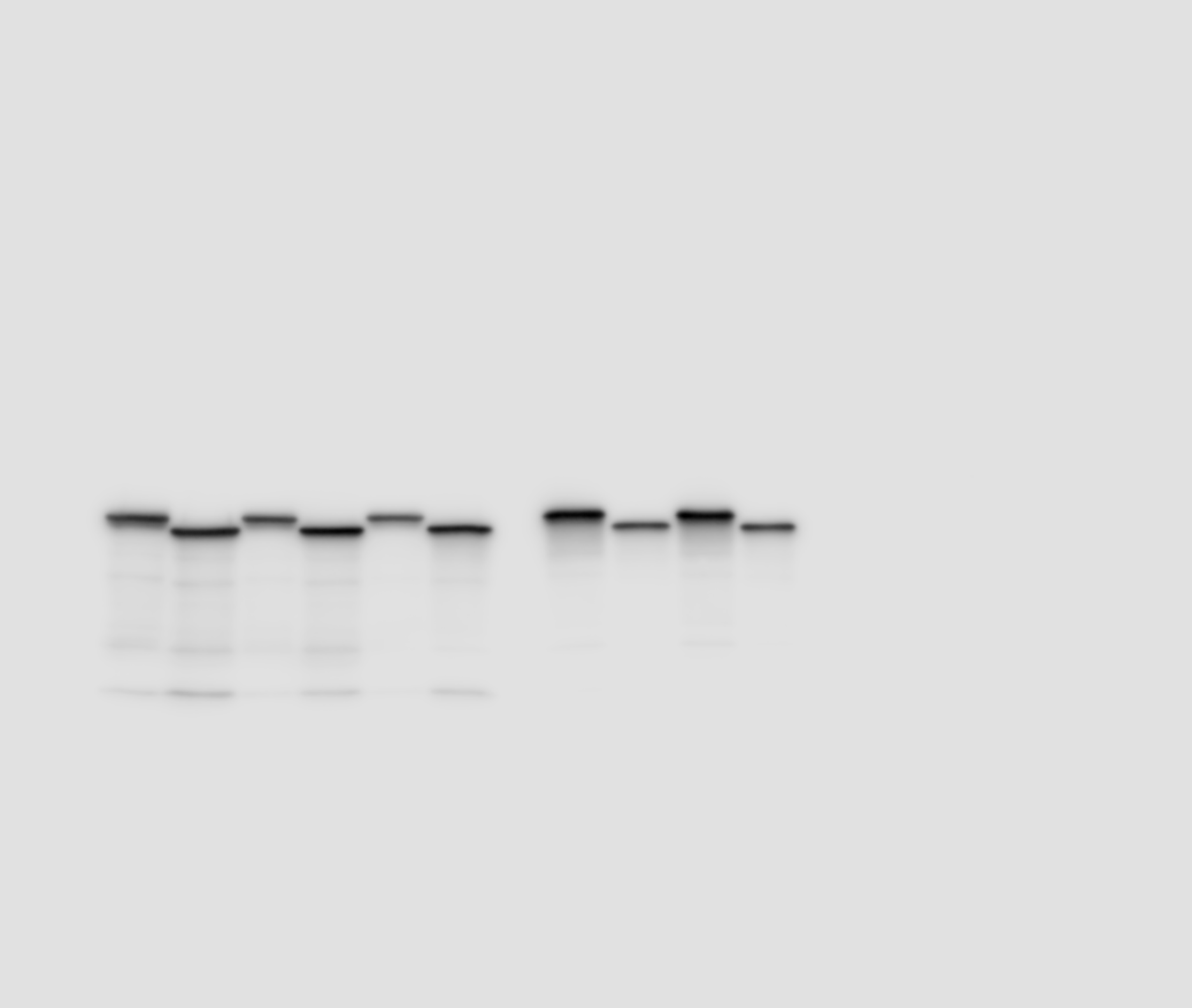

Supplement: Figure 6—source data 1. [file elife-83893-fig6-data1.zip › Figure 6-source data 1/Figure 6-source data 1-raw files/Figure 6-source data 1-right panel-GFP channel.tif]

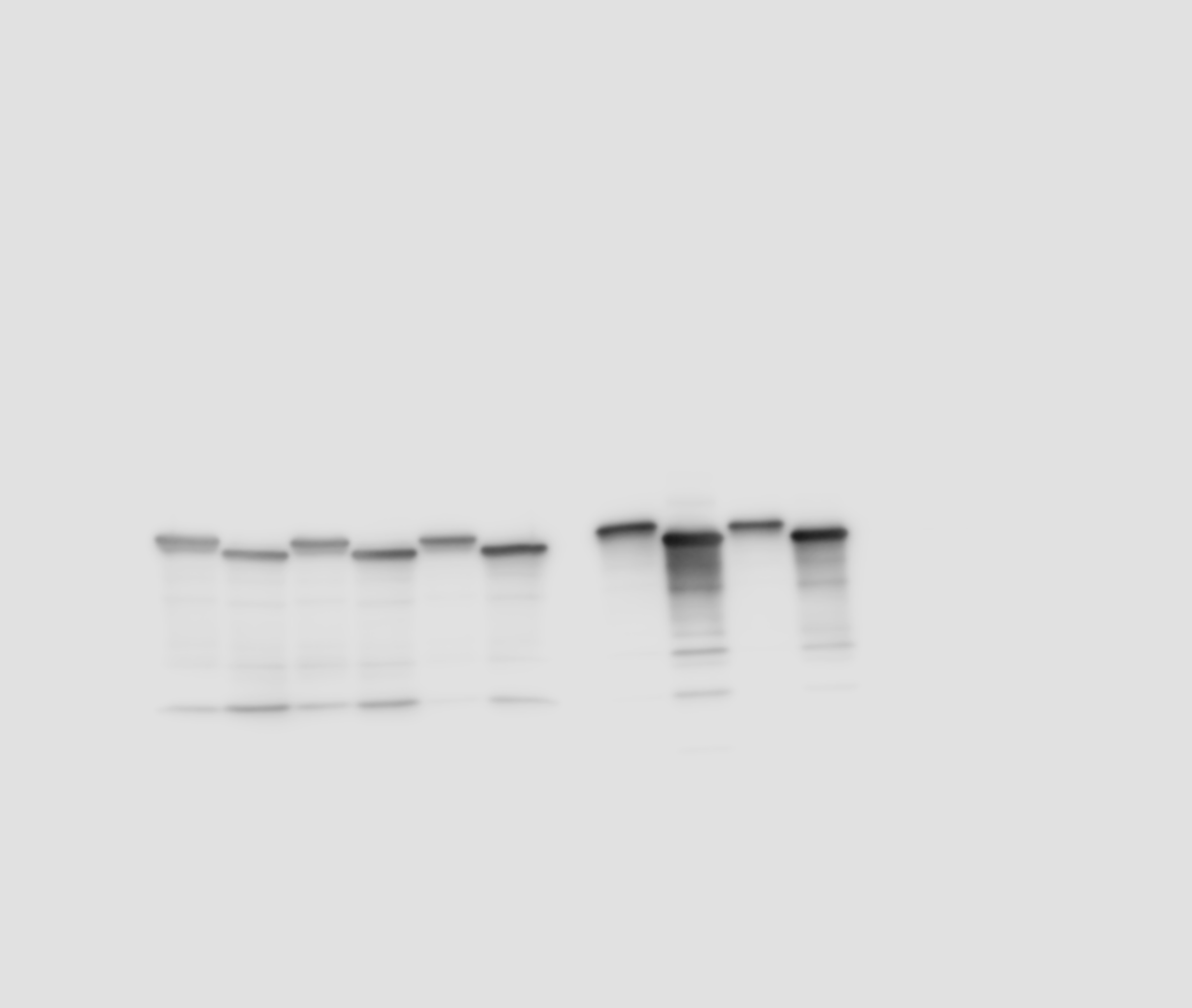

Supplement: Figure 6—source data 1. [file elife-83893-fig6-data1.zip › Figure 6-source data 1/Figure 6-source data 1-raw files/Figure 6-source data 1-left panel-GFP channel.tif]

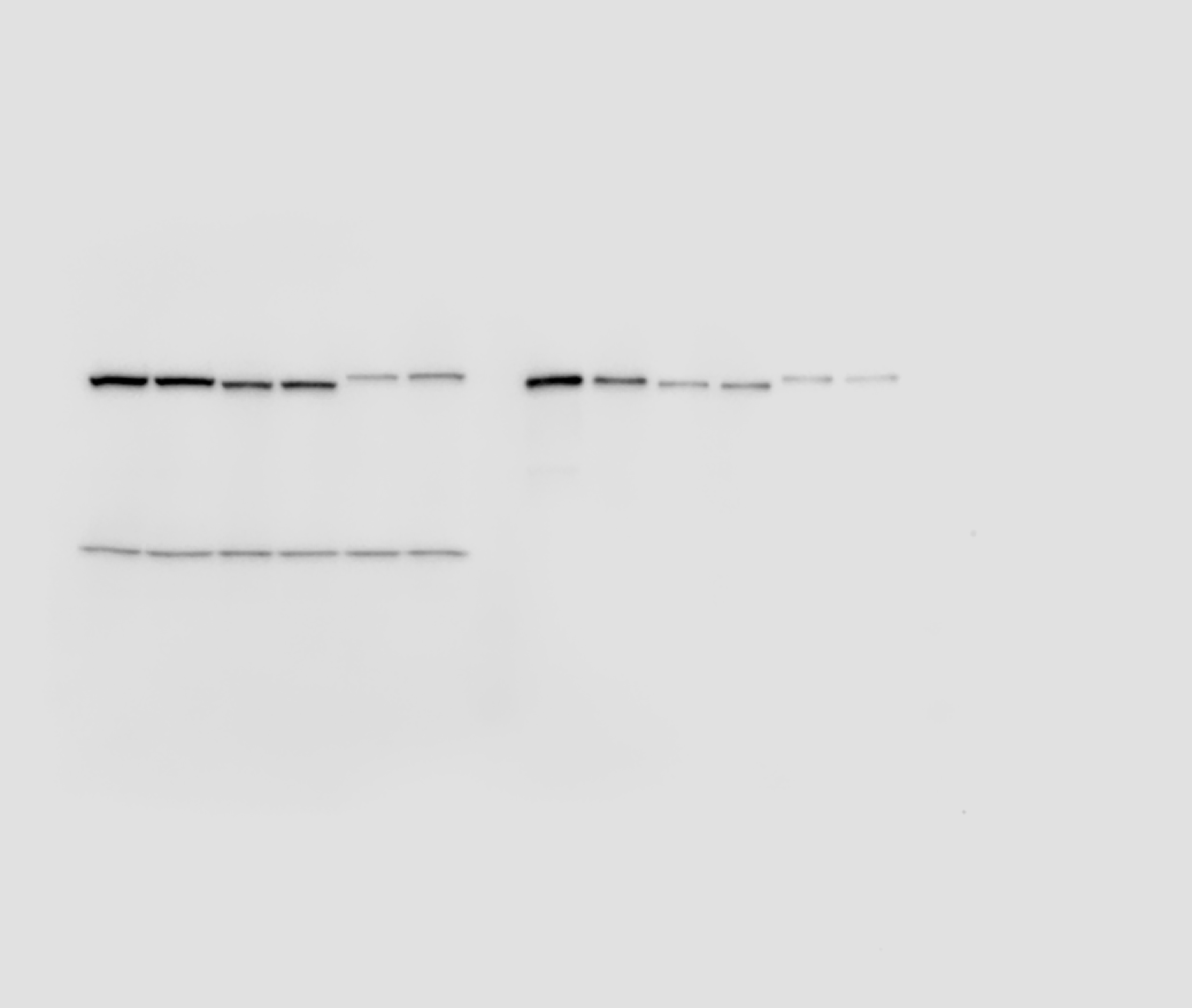

Supplement: Figure 6—source data 1. [file elife-83893-fig6-data1.zip › Figure 6-source data 1/Figure 6-source data 1-raw files/Figure 6-source data 1-right panel-FLAG and GAPDH channel.tif]

Figure 6E

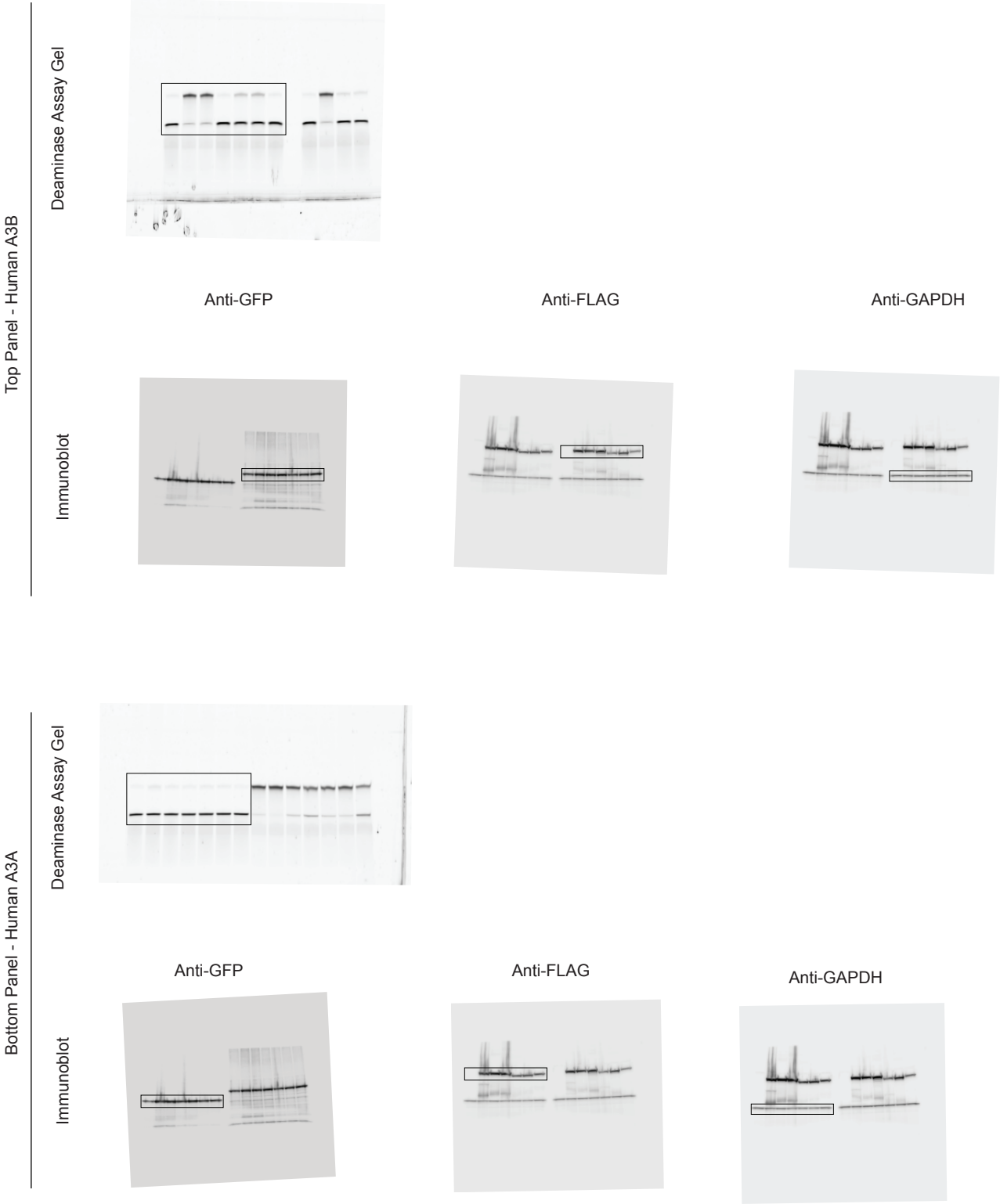

Supplement: Figure 6—source data 2. [file elife-83893-fig6-data2.zip › Figure 6-source data 2/Figure 6-source data 2-uncropped.pdf]

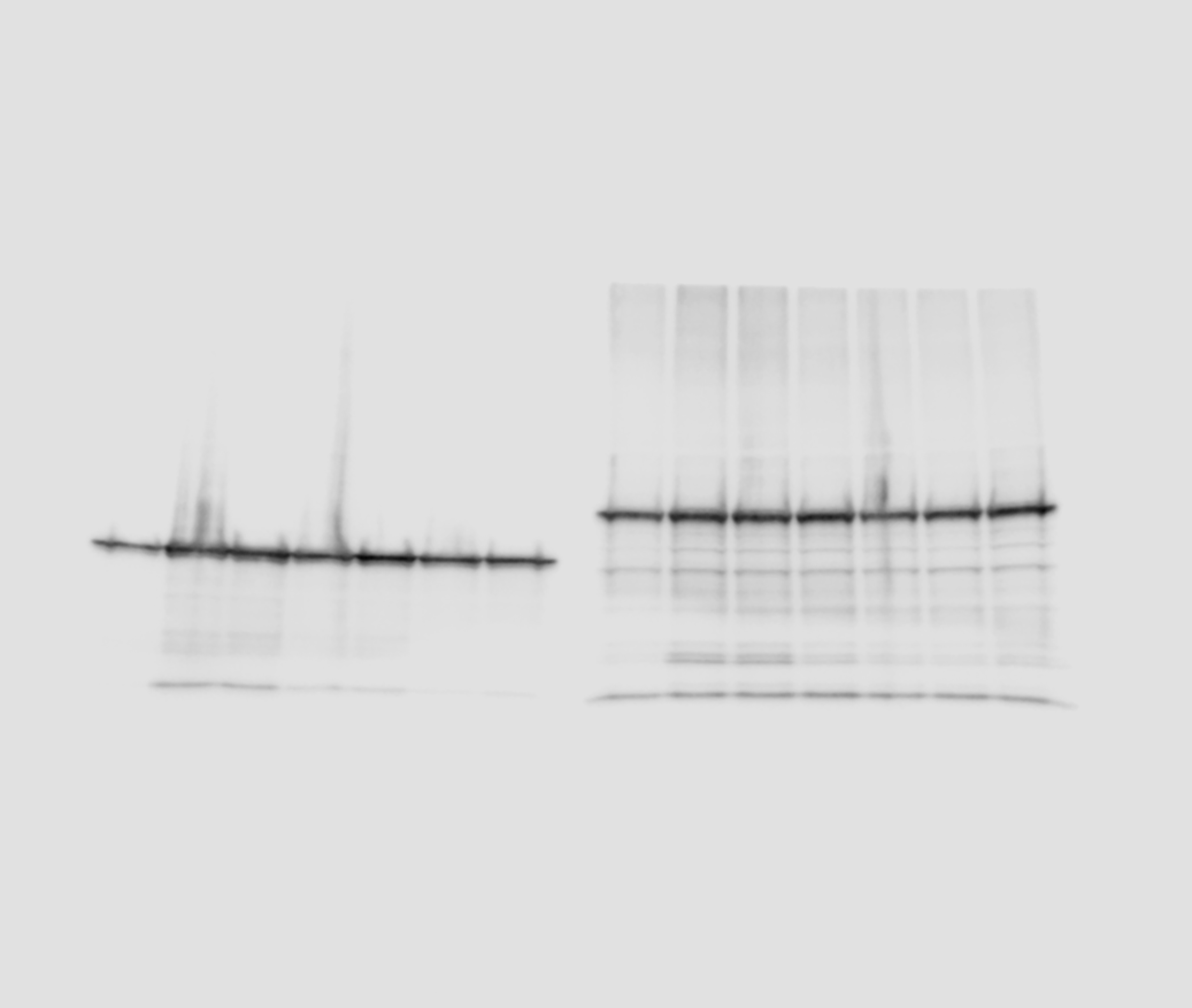

Supplement: Figure 6—source data 2. [file elife-83893-fig6-data2.zip › Figure 6-source data 2/Figure 6-source data 2-raw files/Figure 6-source data 2-immunoblot-GFP channel.tif]

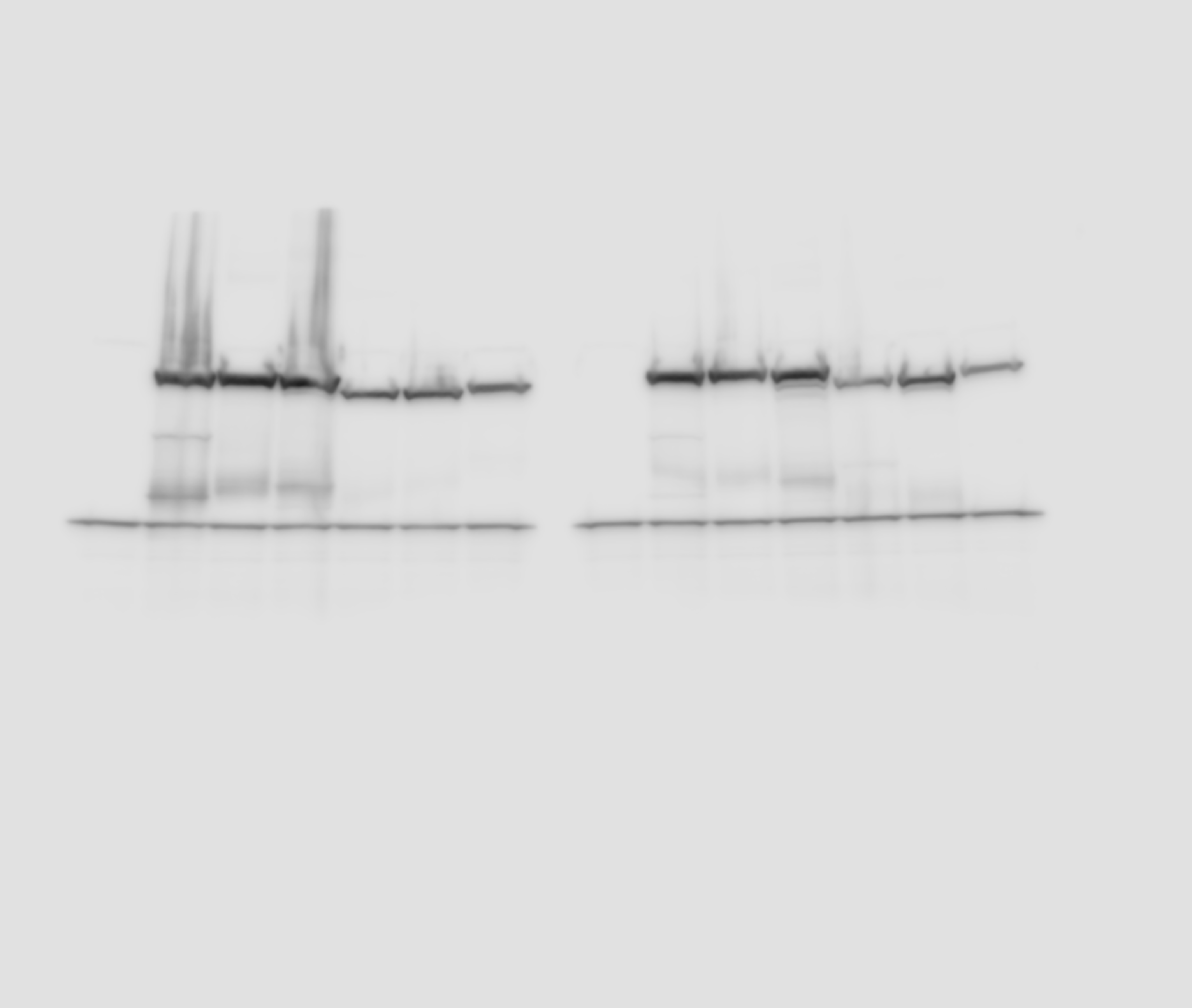

Supplement: Figure 6—source data 2. [file elife-83893-fig6-data2.zip › Figure 6-source data 2/Figure 6-source data 2-raw files/Figure 6-source data 2-immunoblot-FLAG and GAPDH channel.tif]

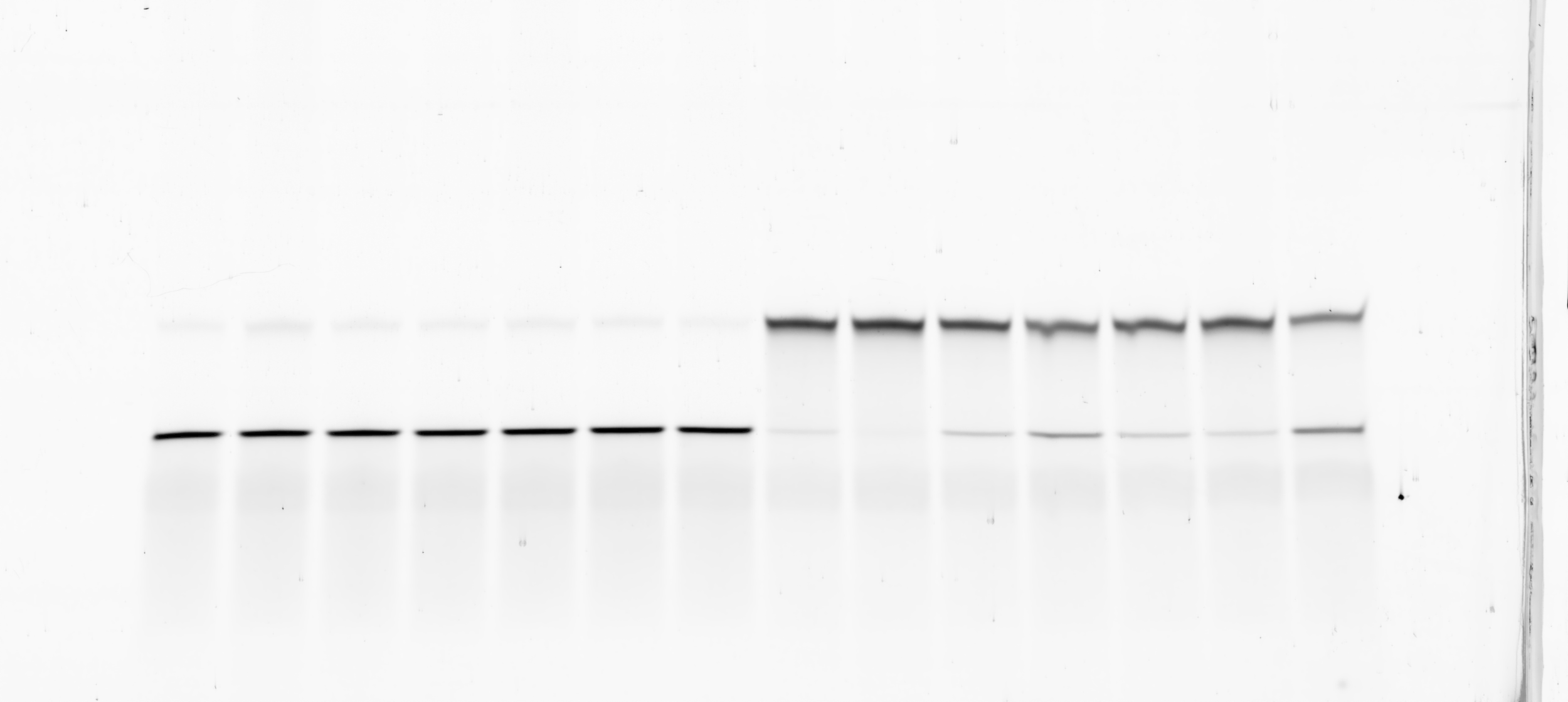

Supplement: Figure 6—source data 2. [file elife-83893-fig6-data2.zip › Figure 6-source data 2/Figure 6-source data 2-raw files/Figure 6-source data 2-bottom panel-deaminase assay gel.tif]

Figure 7A

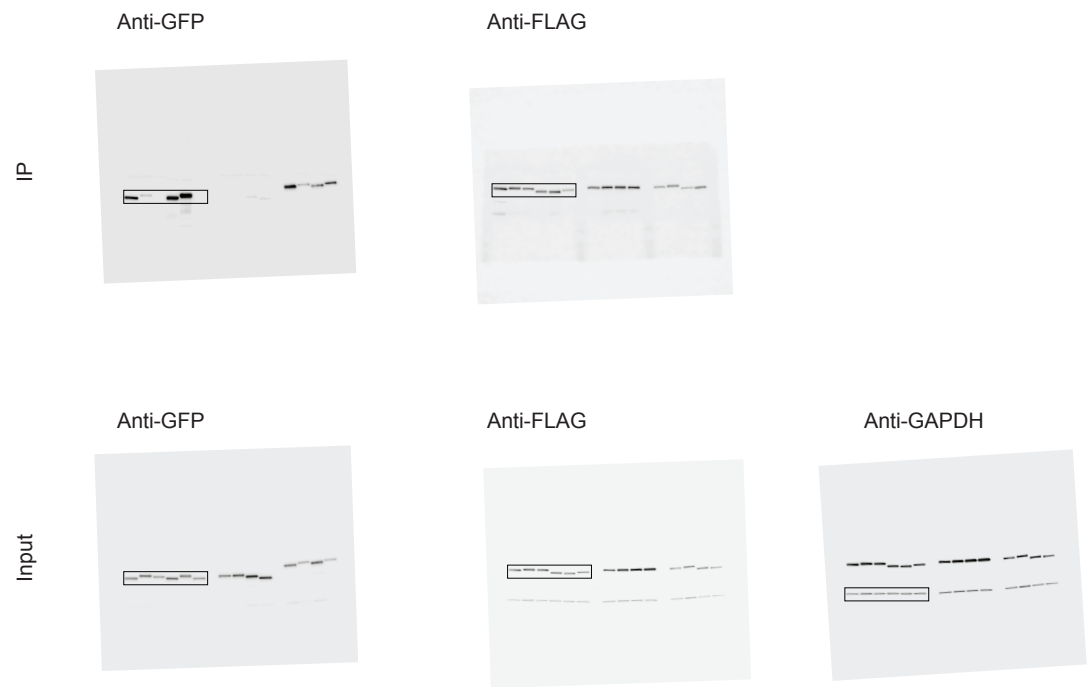

Supplement: Figure 7—source data 1. [file elife-83893-fig7-data1.zip › Figure 7-source data 1/Figure 7-source data 1-uncropped.pdf]

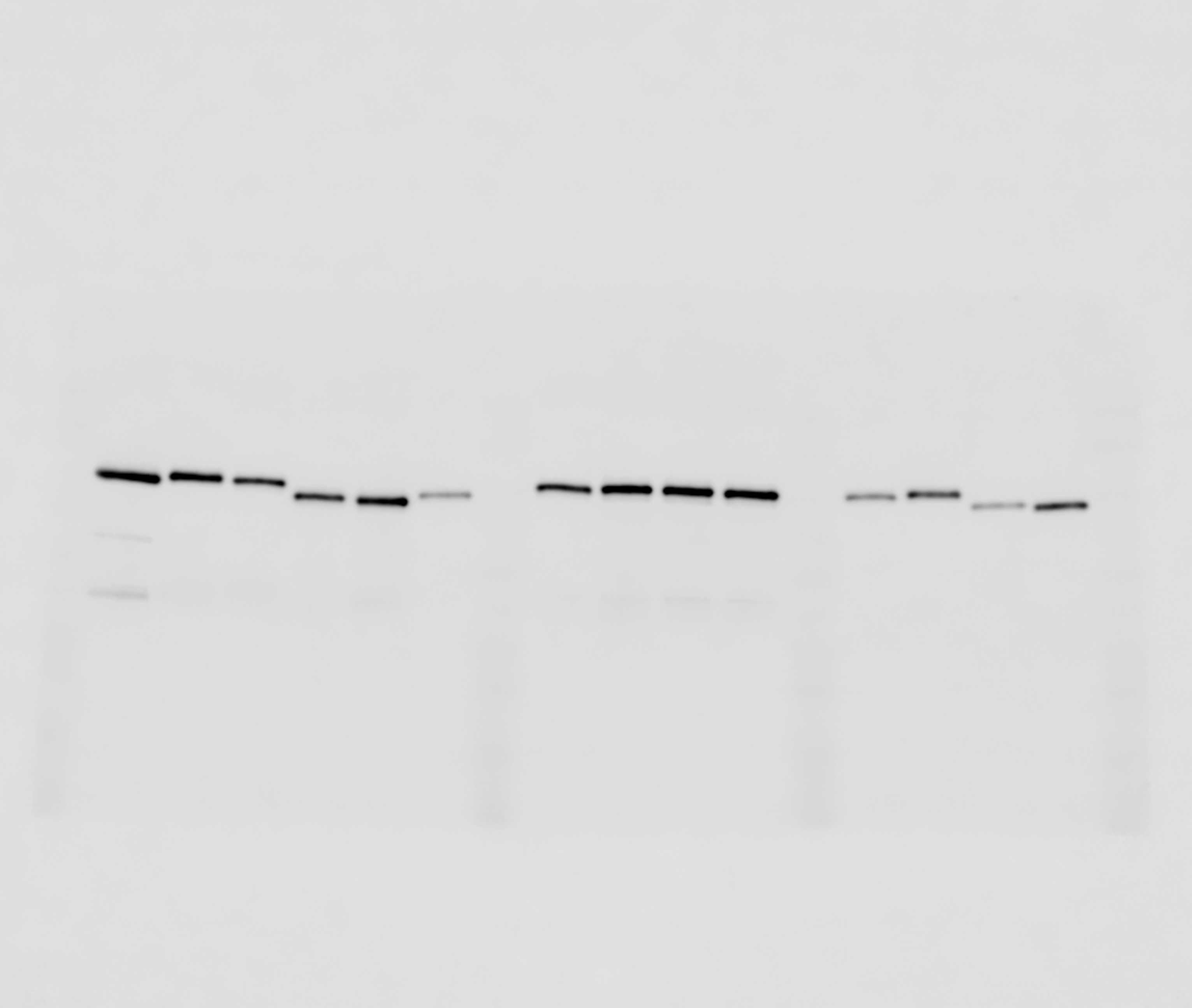

Supplement: Figure 7—source data 1. [file elife-83893-fig7-data1.zip › Figure 7-source data 1/Figure 7-source data 1-raw files/Figure 7-source data 1-IP-FLAG channel.tif]

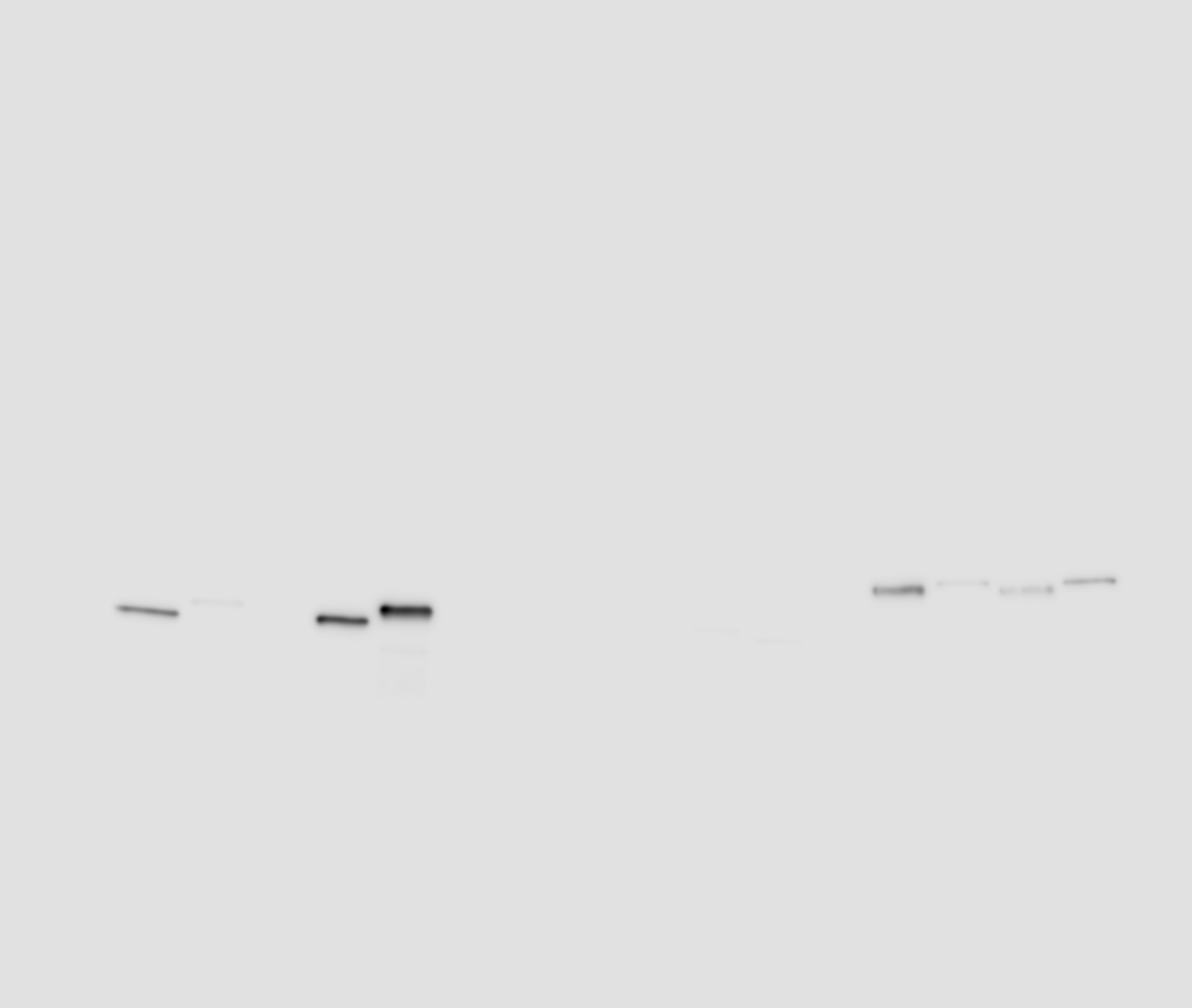

Supplement: Figure 7—source data 1. [file elife-83893-fig7-data1.zip › Figure 7-source data 1/Figure 7-source data 1-raw files/Figure 7-source data 1-IP-GFP channel.tif]

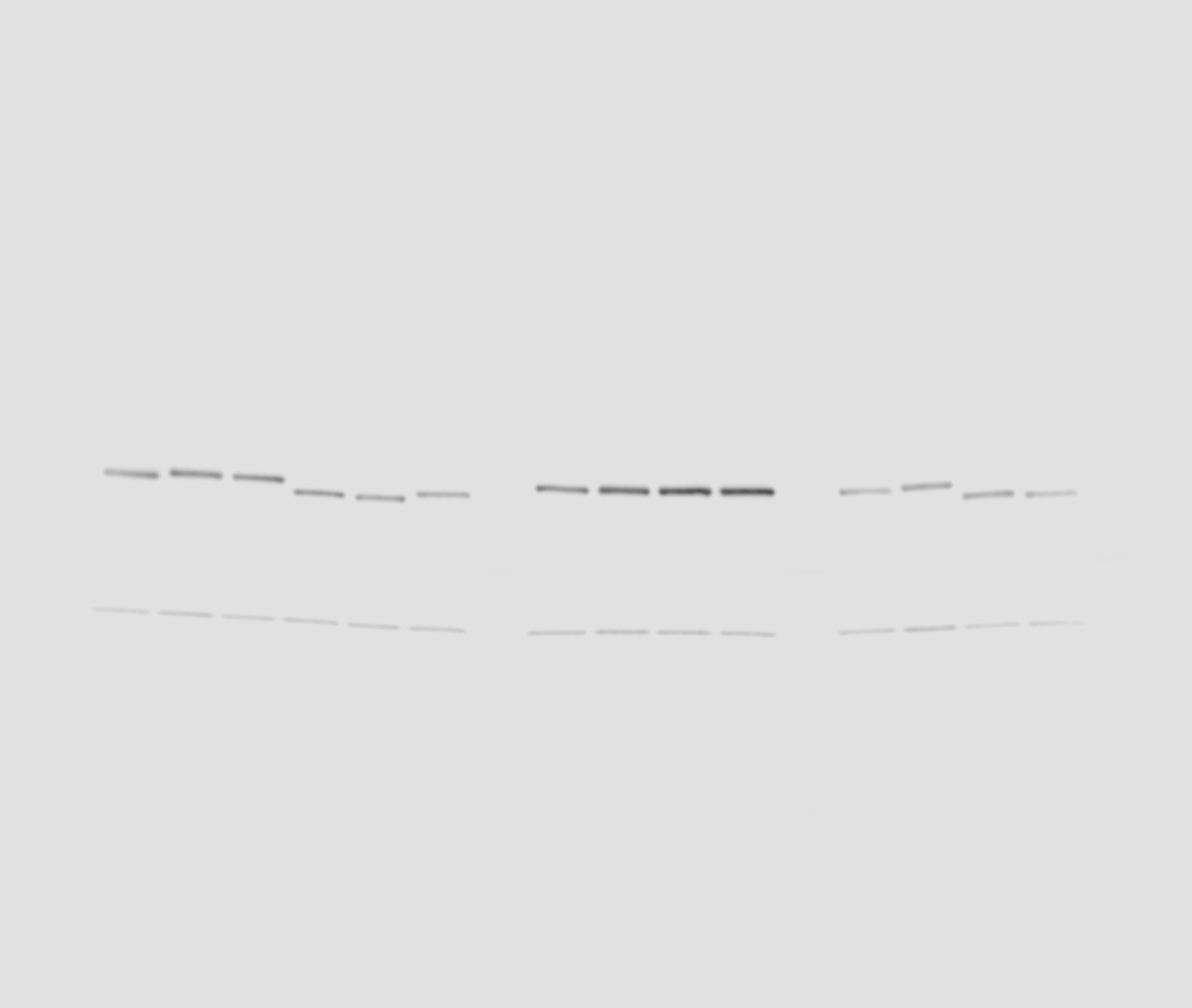

Supplement: Figure 7—source data 1. [file elife-83893-fig7-data1.zip › Figure 7-source data 1/Figure 7-source data 1-raw files/Figure 7-source data 1-input-FLAG and GAPDH channel.tif]

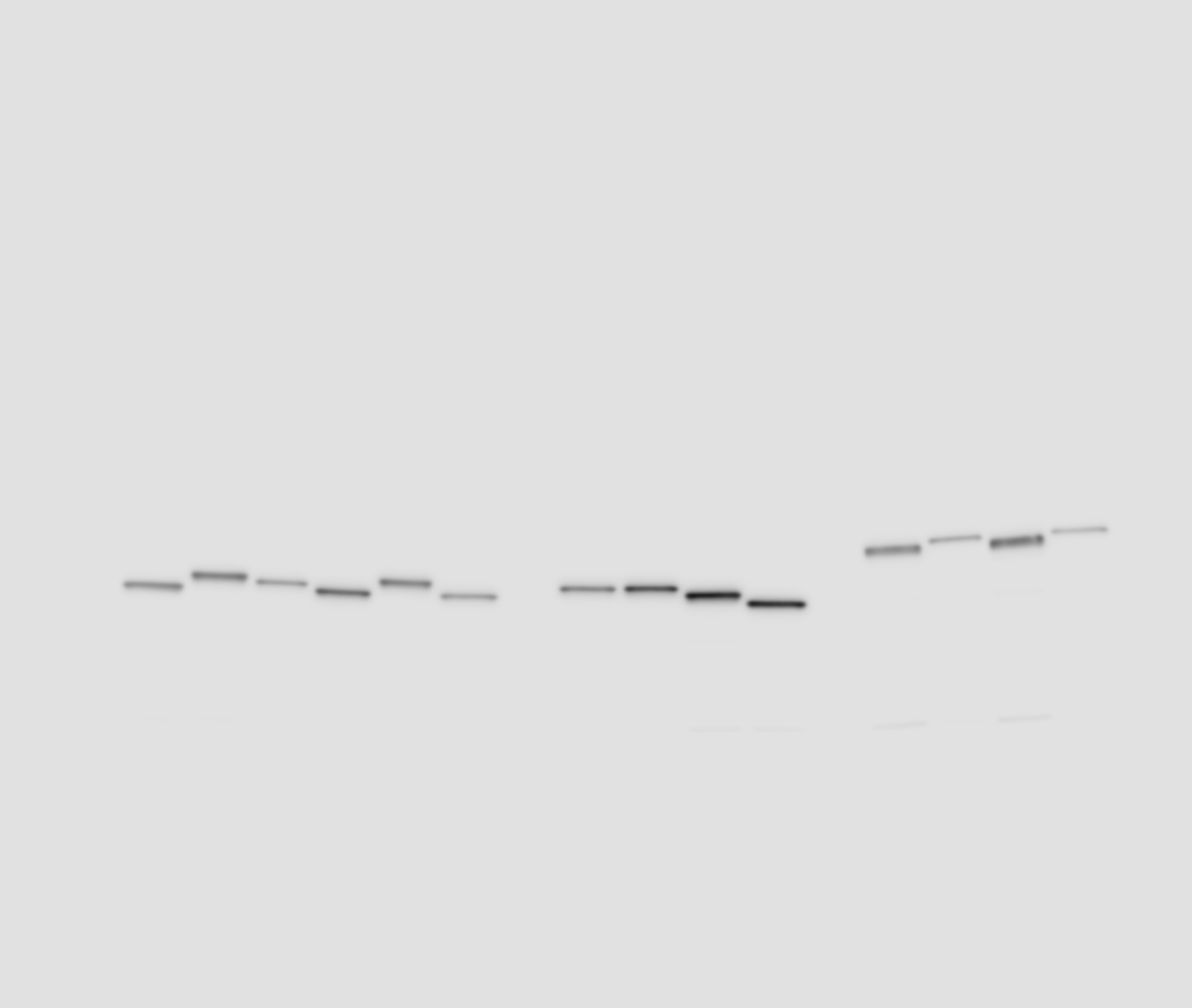

Supplement: Figure 7—source data 1. [file elife-83893-fig7-data1.zip › Figure 7-source data 1/Figure 7-source data 1-raw files/Figure 7-source data 1-input-GFP channel.tif]

Figure 7D

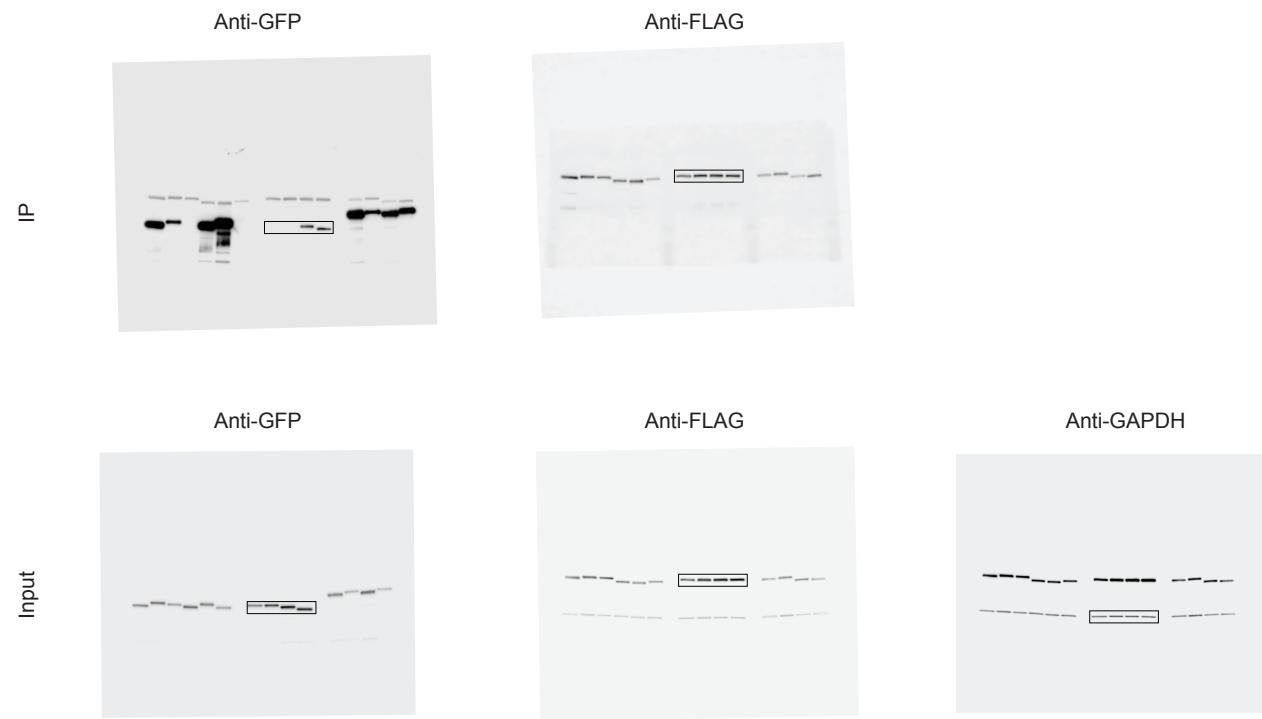

Supplement: Figure 7—source data 2. [file elife-83893-fig7-data2.zip › Figure 7-source data 2/Figure 7-source data 2-uncropped.pdf]

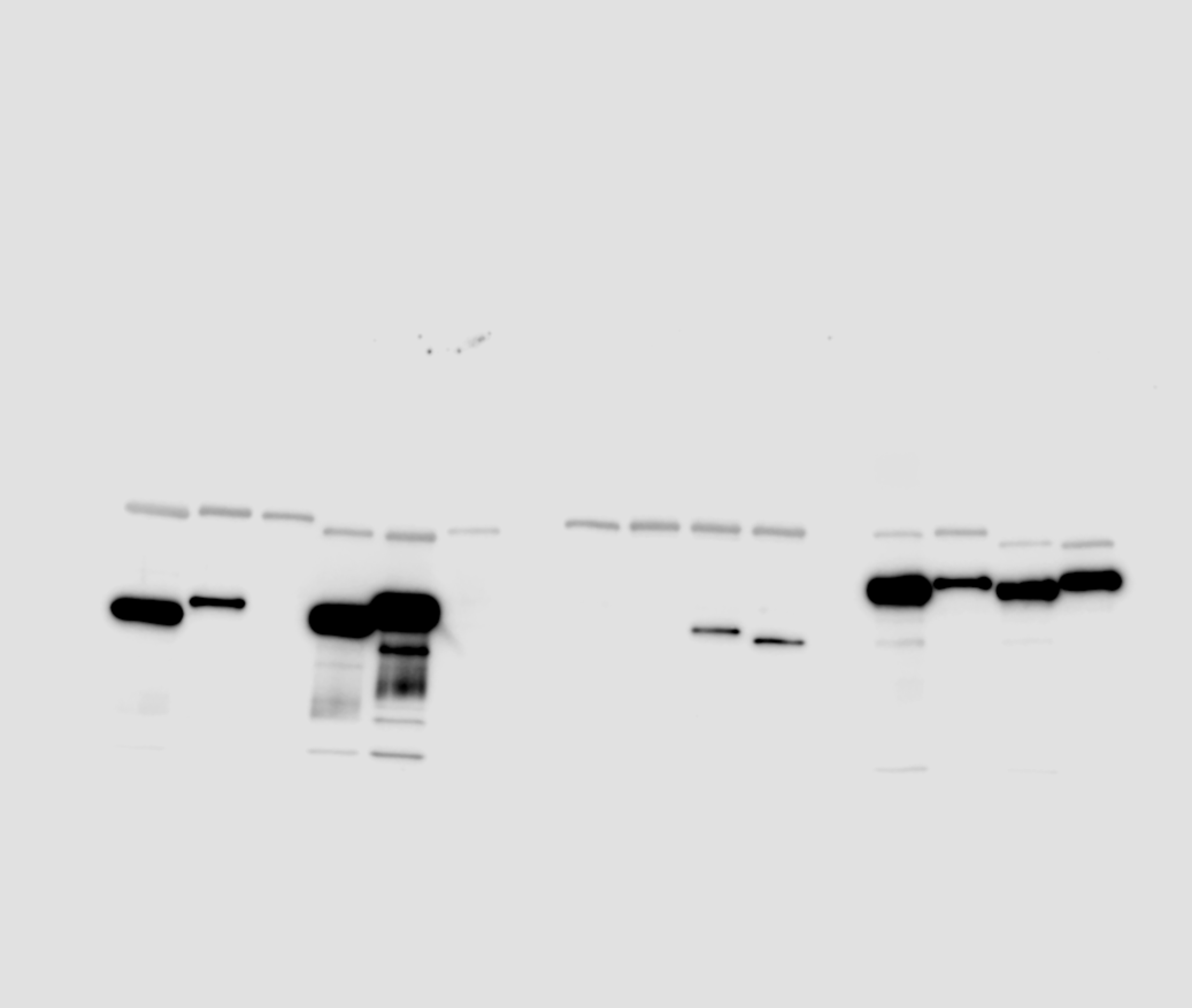

Supplement: Figure 7—source data 2. [file elife-83893-fig7-data2.zip › Figure 7-source data 2/Figure 7-source data 2-raw files/Figure 7-source data 2-IP-GFP channel.tif]

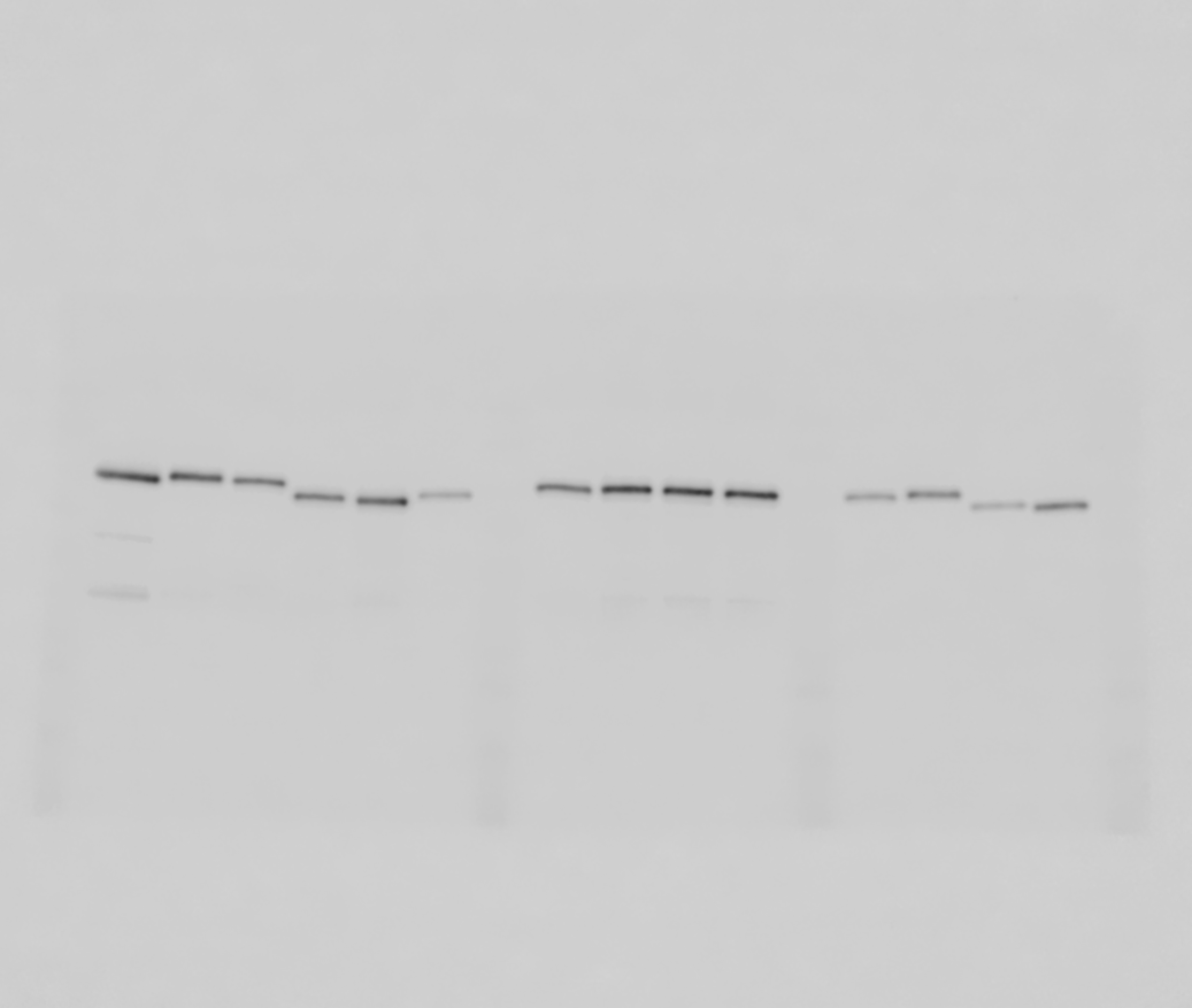

Supplement: Figure 7—source data 2. [file elife-83893-fig7-data2.zip › Figure 7-source data 2/Figure 7-source data 2-raw files/Figure 7-source data 2-IP-FLAG channel.tif]

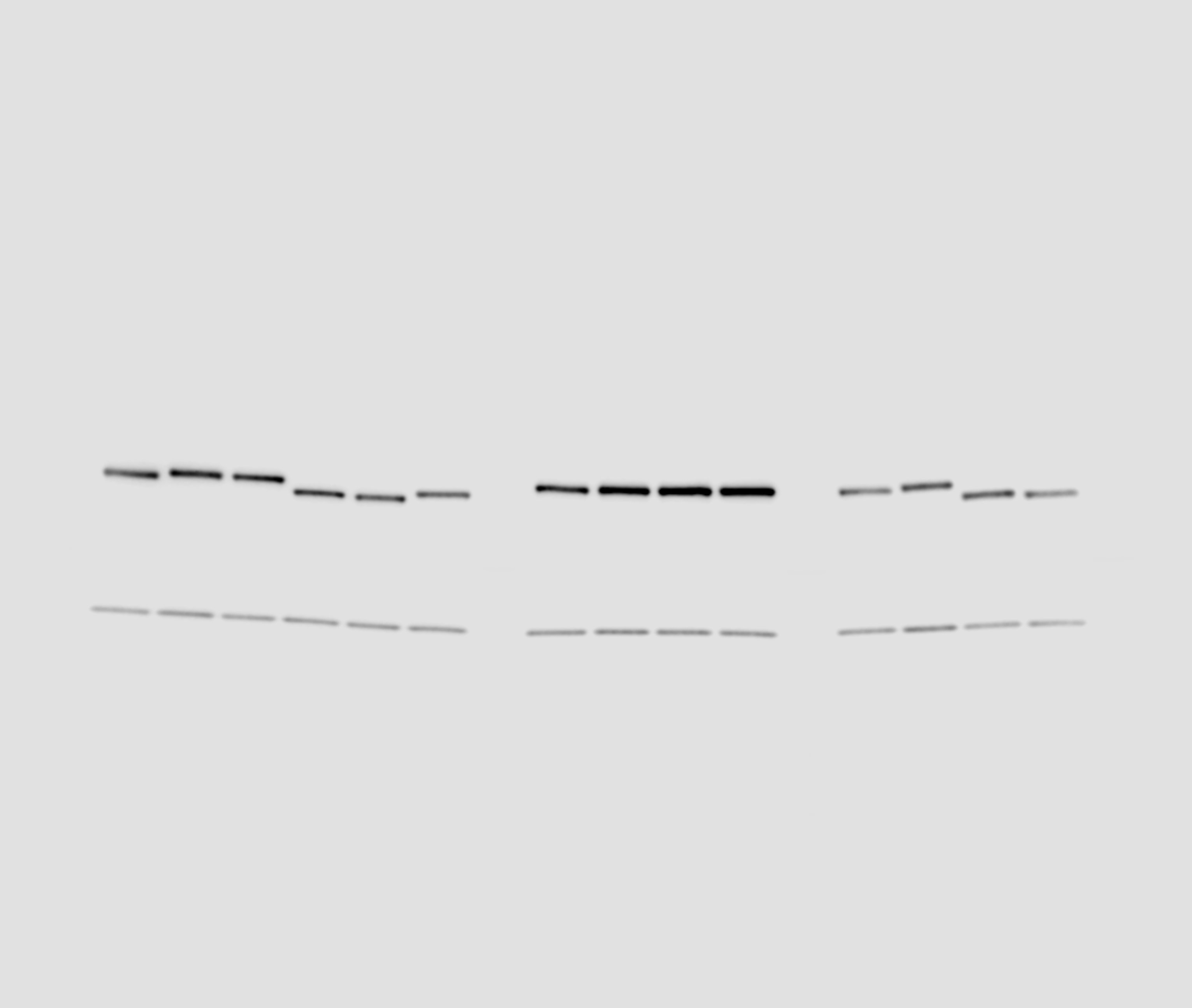

Supplement: Figure 7—source data 2. [file elife-83893-fig7-data2.zip › Figure 7-source data 2/Figure 7-source data 2-raw files/Figure 7-source data 2-input-FLAG and GAPDH channel.tif]

Figure 8B

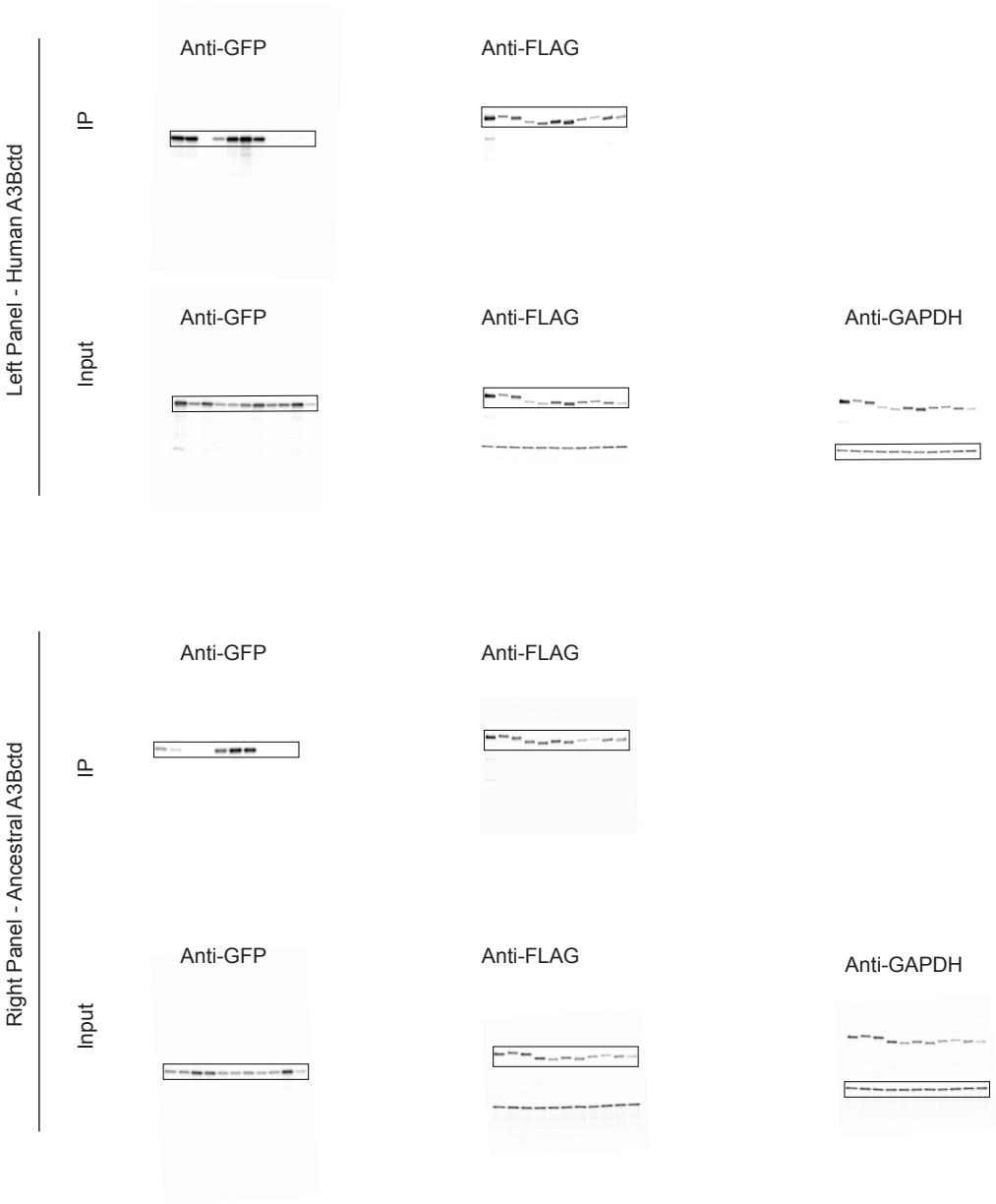

Supplement: Figure 8—source data 1. [file elife-83893-fig8-data1.zip › Figure 8-source data 1/Figure 8-source data 1-uncropped.pdf]

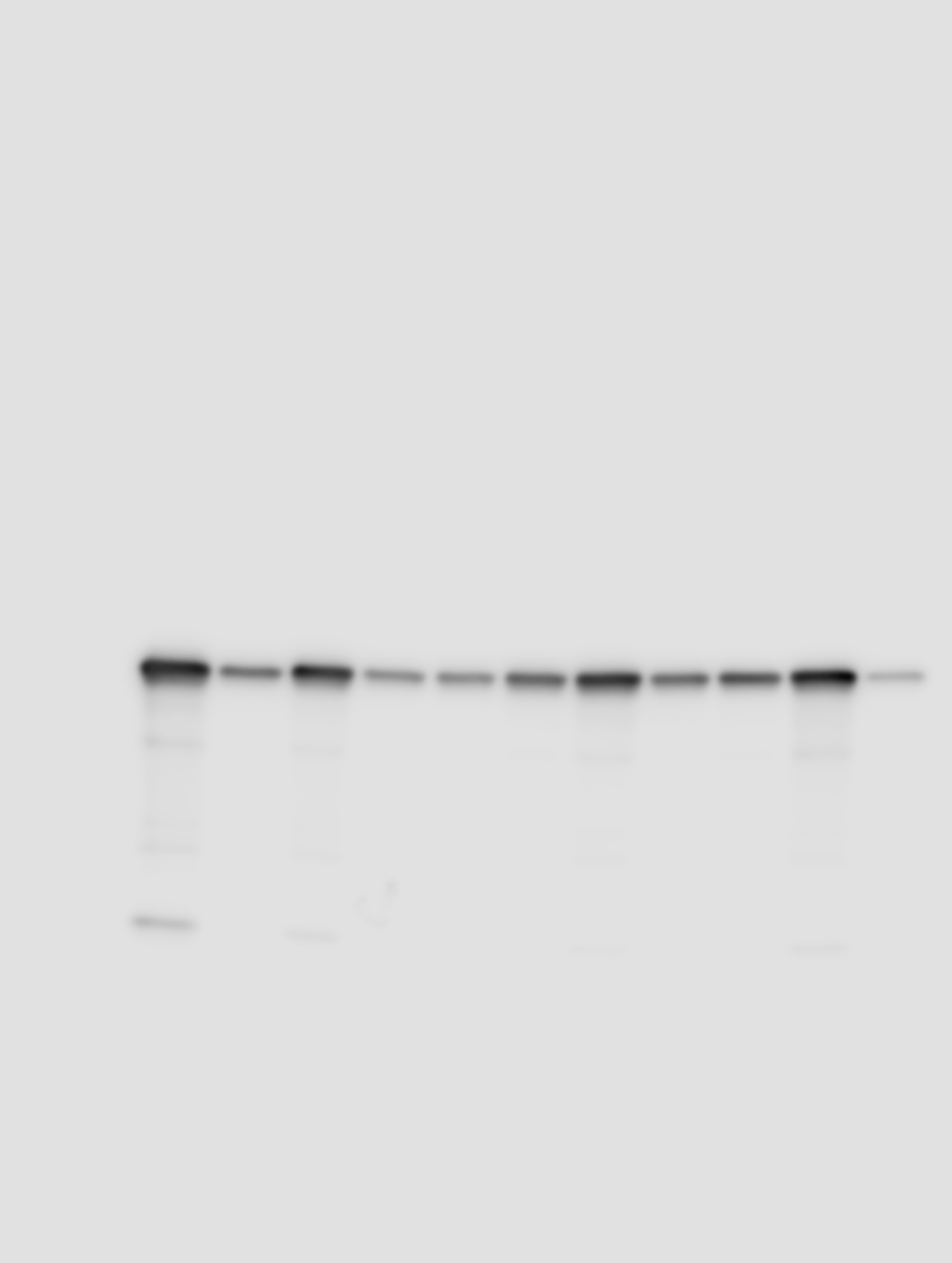

Supplement: Figure 8—source data 1. [file elife-83893-fig8-data1.zip › Figure 8-source data 1/Figure 8-source data 1-raw files/Figure 8-source data 1-left panel-input-GFP channel.tif]

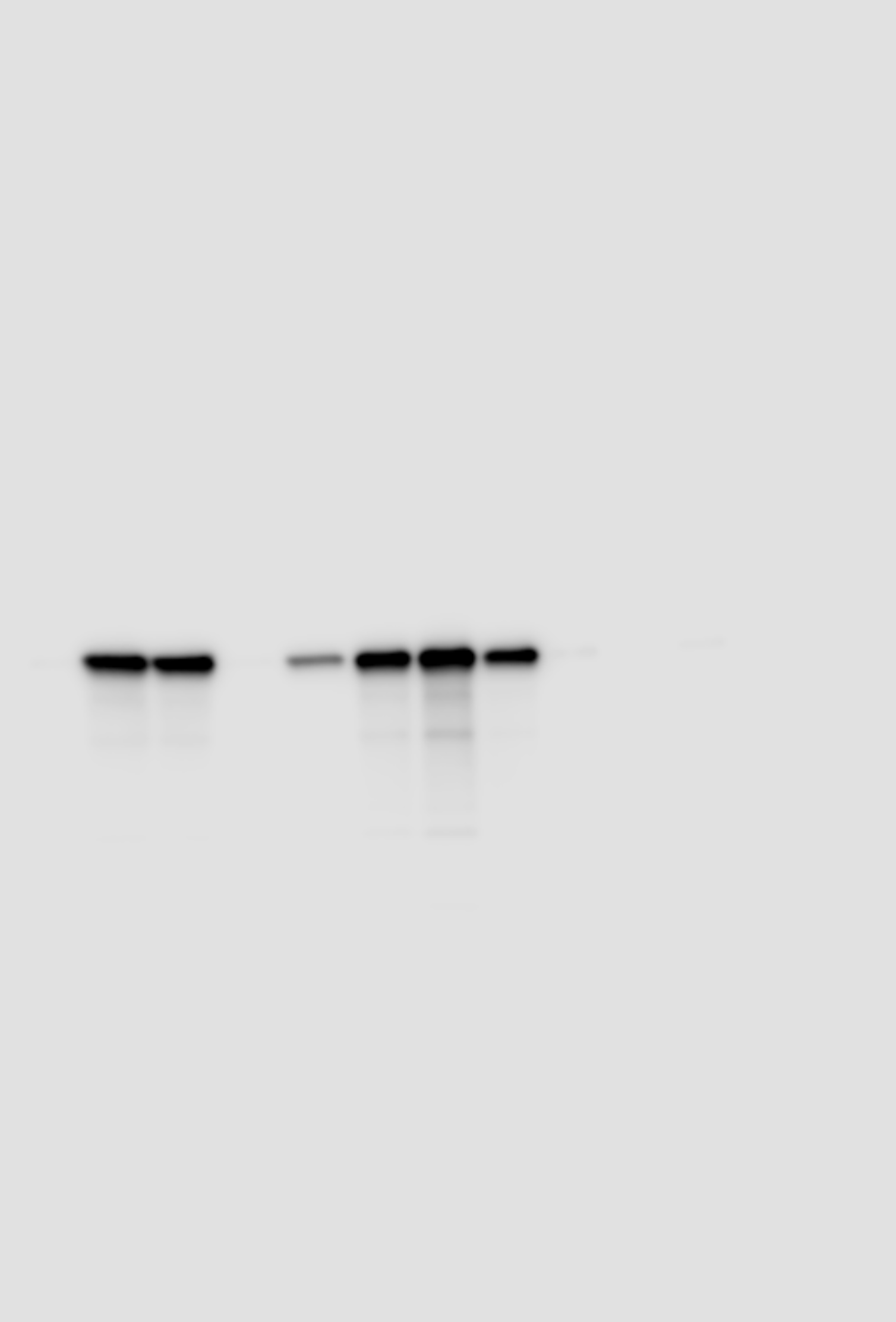

Supplement: Figure 8—source data 1. [file elife-83893-fig8-data1.zip › Figure 8-source data 1/Figure 8-source data 1-raw files/Figure 8-source data 1-left panel-IP-GFP channel.tif]

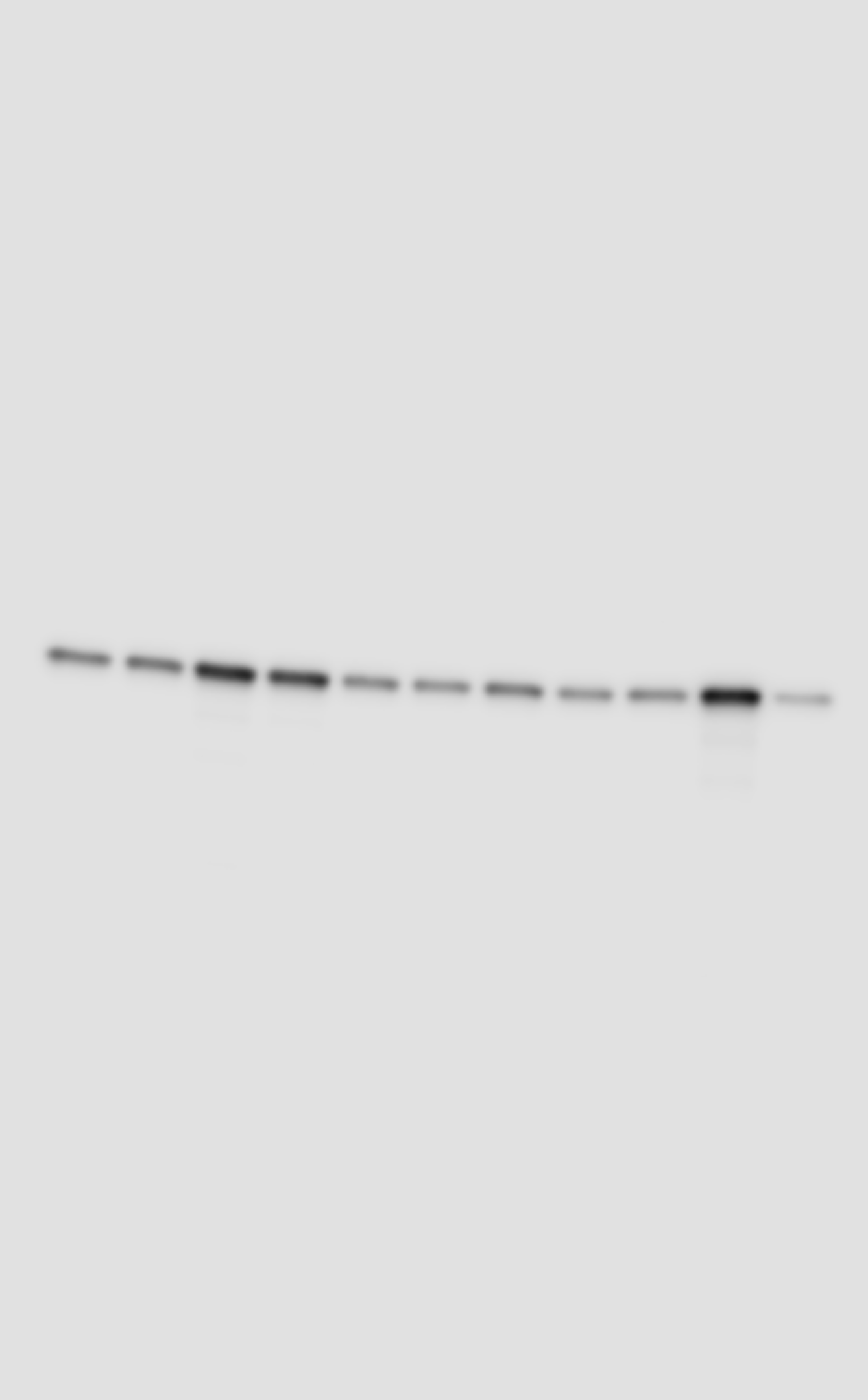

Supplement: Figure 8—source data 1. [file elife-83893-fig8-data1.zip › Figure 8-source data 1/Figure 8-source data 1-raw files/Figure 8-source data 1-right panel-input-GFP channel.tif]

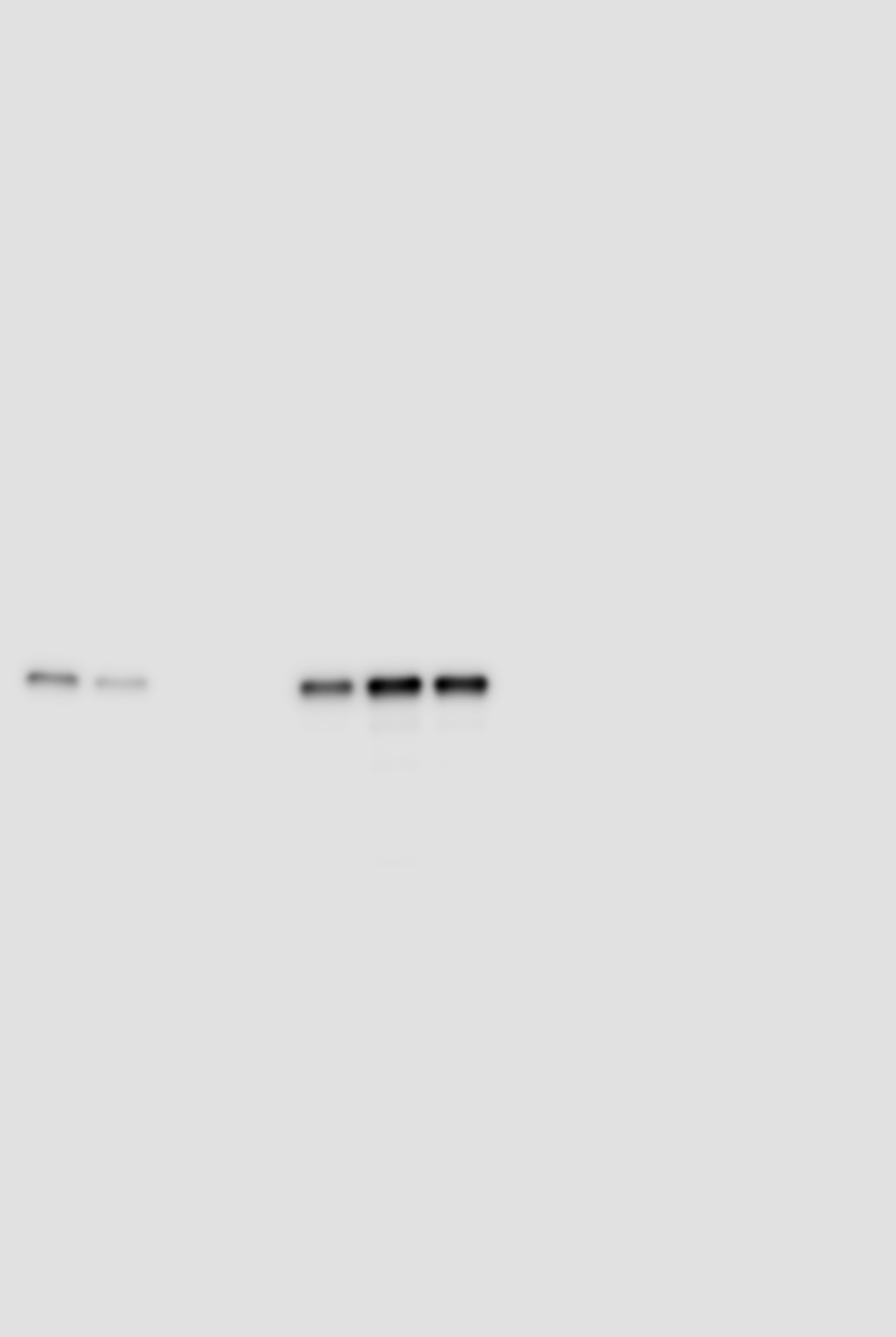

Supplement: Figure 8—source data 1. [file elife-83893-fig8-data1.zip › Figure 8-source data 1/Figure 8-source data 1-raw files/Figure 8-source data 1-right panel-IP-GFP channel.tif]

Figure 8D

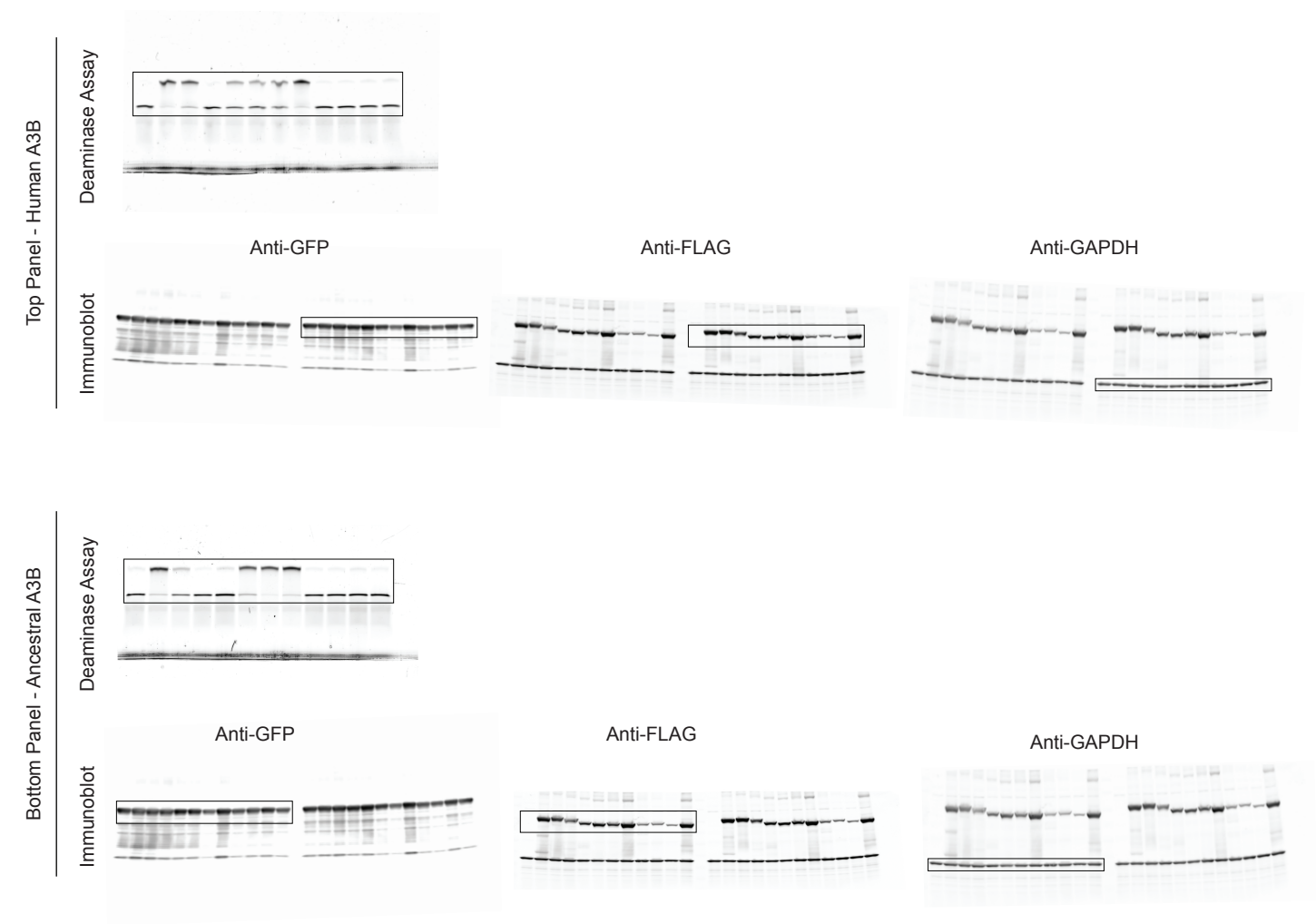

Supplement: Figure 8—source data 2. [file elife-83893-fig8-data2.zip › Figure 8-source data 2/Figure 8-source data 2-uncropped.pdf]

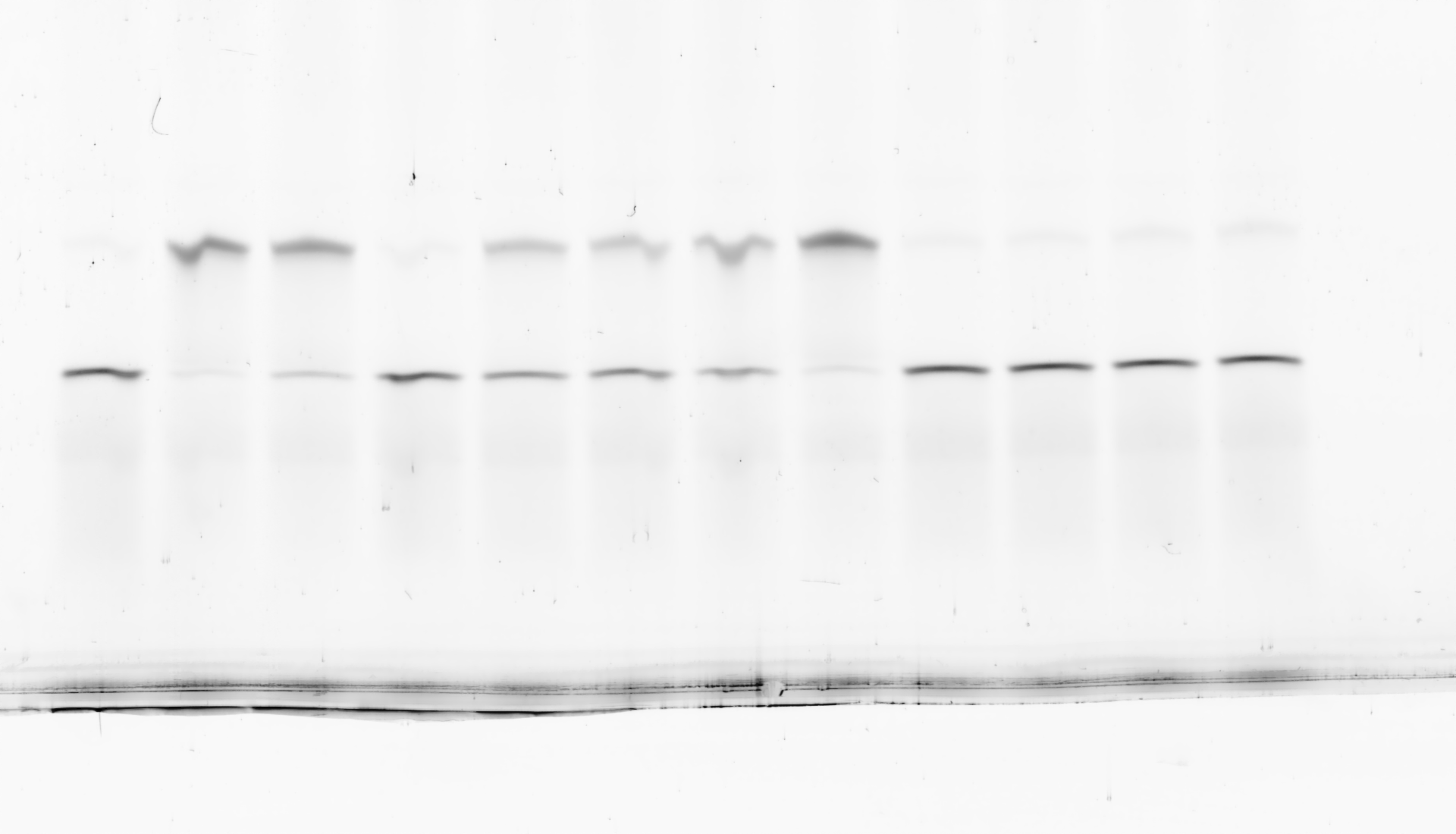

Supplement: Figure 8—source data 2. [file elife-83893-fig8-data2.zip › Figure 8-source data 2/Figure 8-source data 2-raw files/Figure 8-source data 2-deaminase assay gel-top panel.tif]

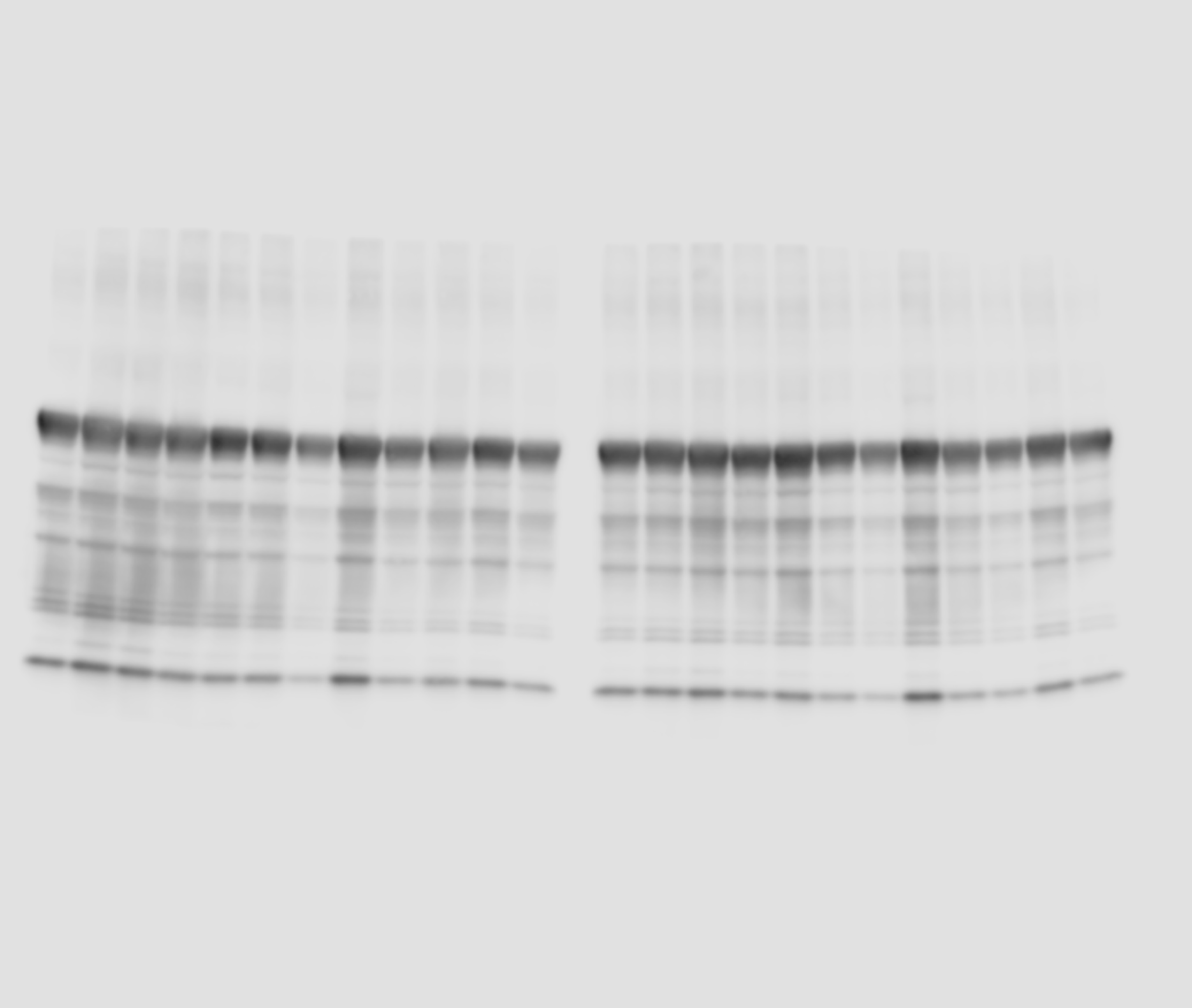

Supplement: Figure 8—source data 2. [file elife-83893-fig8-data2.zip › Figure 8-source data 2/Figure 8-source data 2-raw files/Figure 8-source data 2-immunoblot-GFP channel.tif]

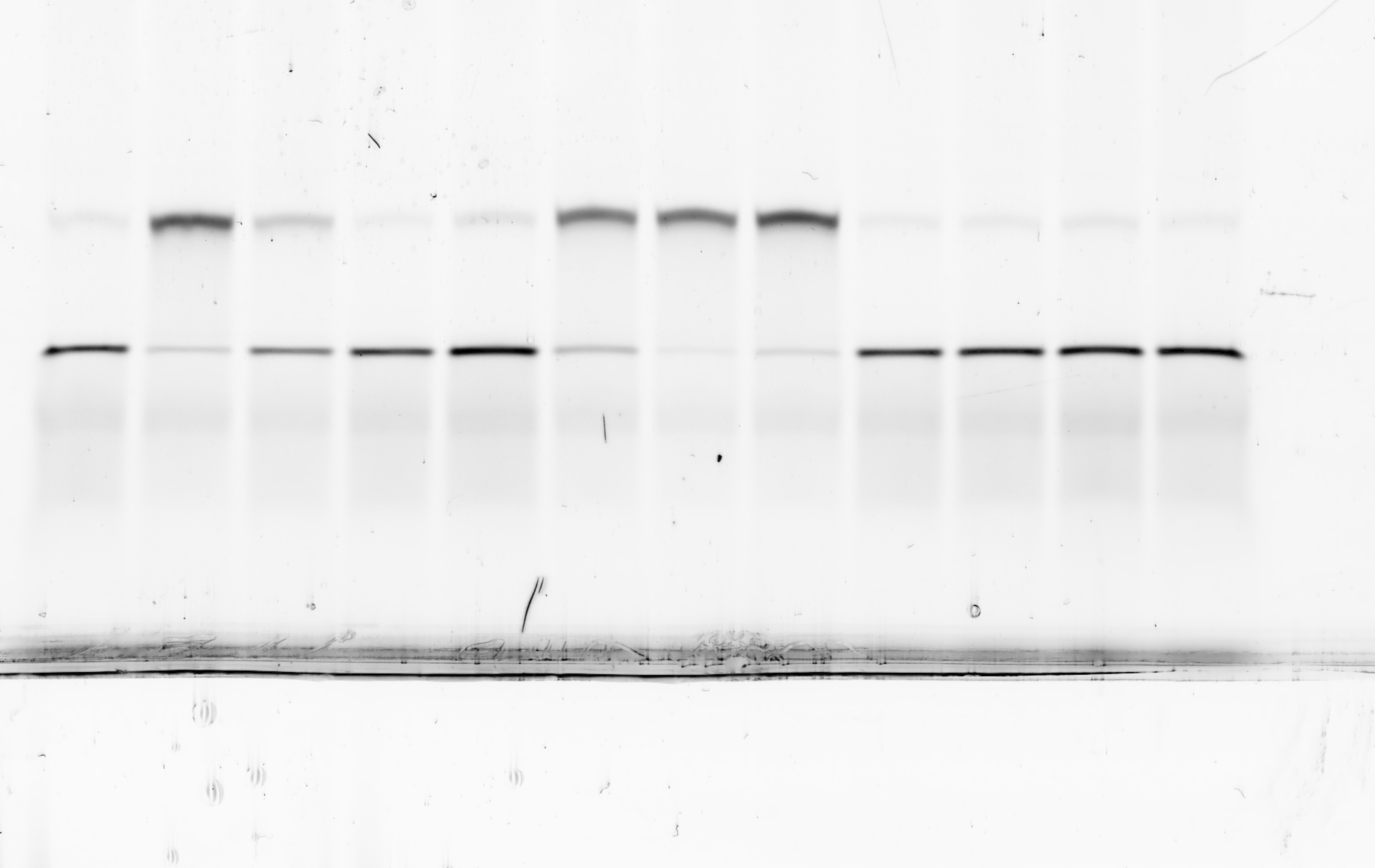

Supplement: Figure 8—source data 2. [file elife-83893-fig8-data2.zip › Figure 8-source data 2/Figure 8-source data 2-raw files/Figure 8-source data 2-deaminase assay gel-bottom panel.tif]

Figure 8—figure supplement 2, panel B

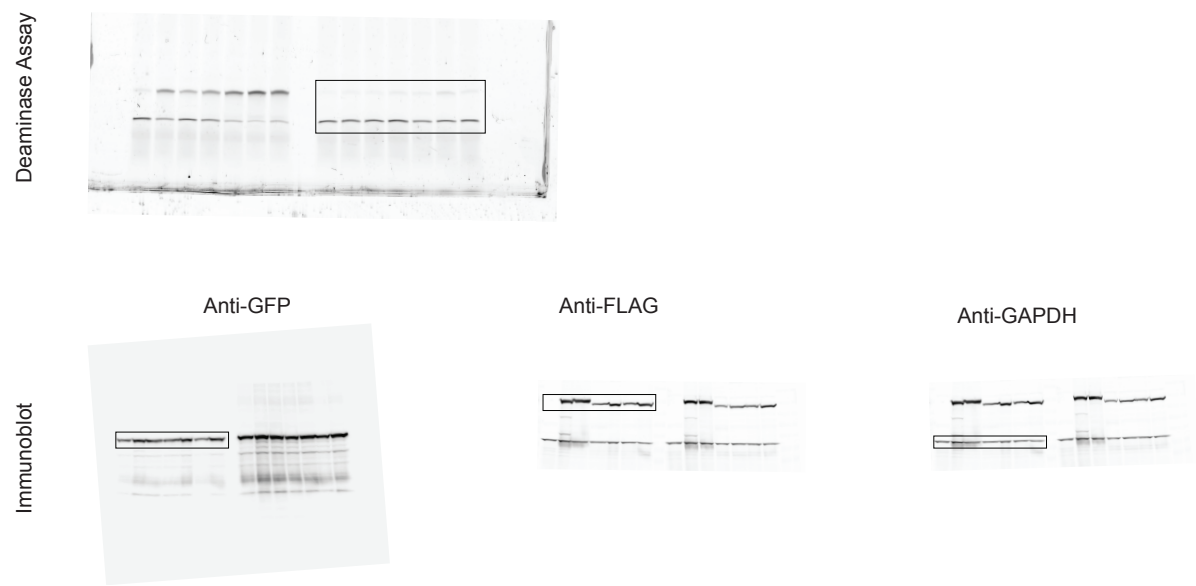

Supplement: Figure 8—figure supplement 2—source data 1. [file elife-83893-fig8-figsupp2-data1.zip › Figure 8-figure supplement 2-source data 1/Figure 8-figure supplement 2-source data 1-uncropped.pdf]

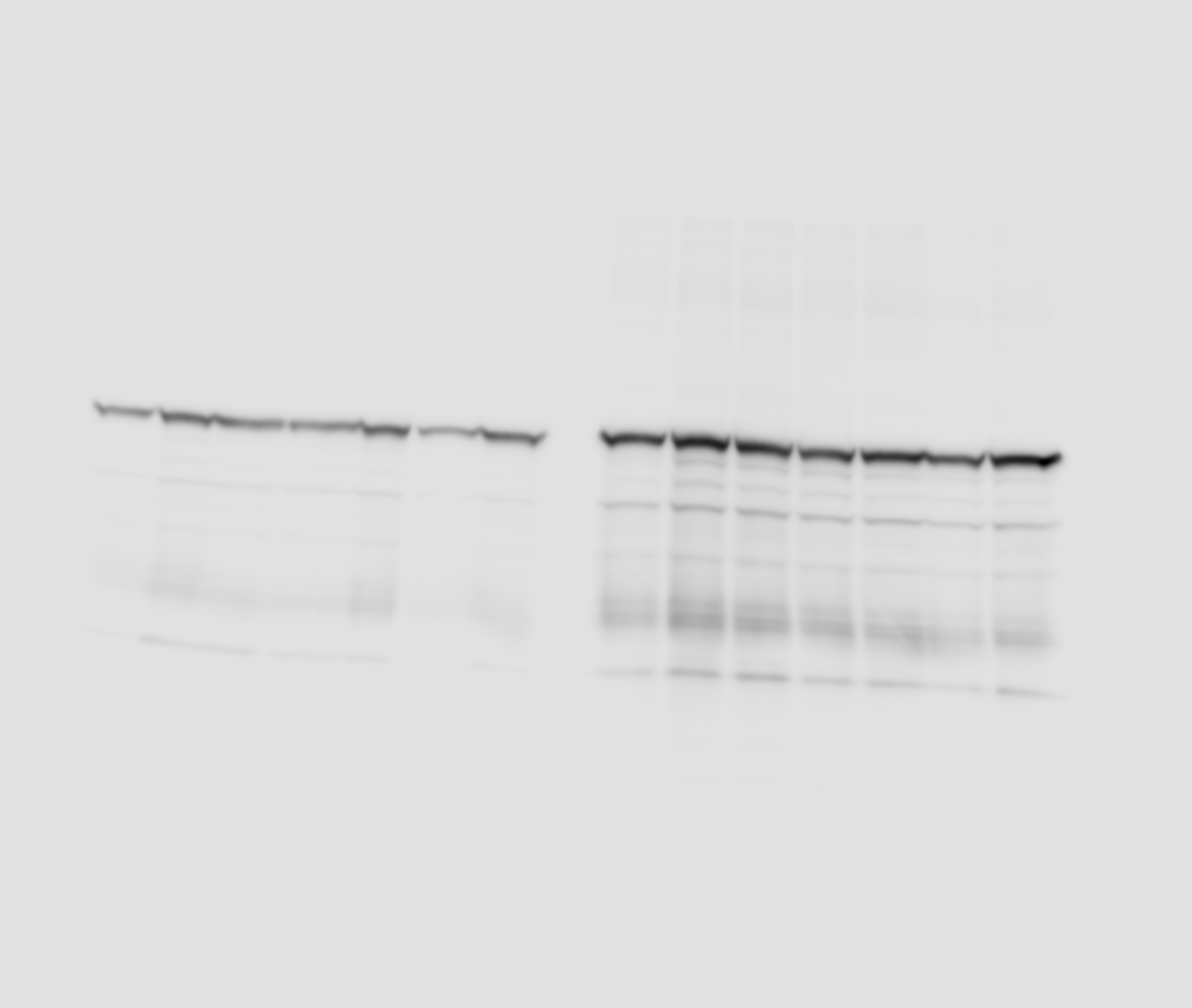

Supplement: Figure 8—figure supplement 2—source data 1. [file elife-83893-fig8-figsupp2-data1.zip › Figure 8-figure supplement 2-source data 1/Figure 8-figure supplement 2-source data 1-raw files/Figure 8-figure supplement 2-source data 1-immunoblot-GFP channel.tif]

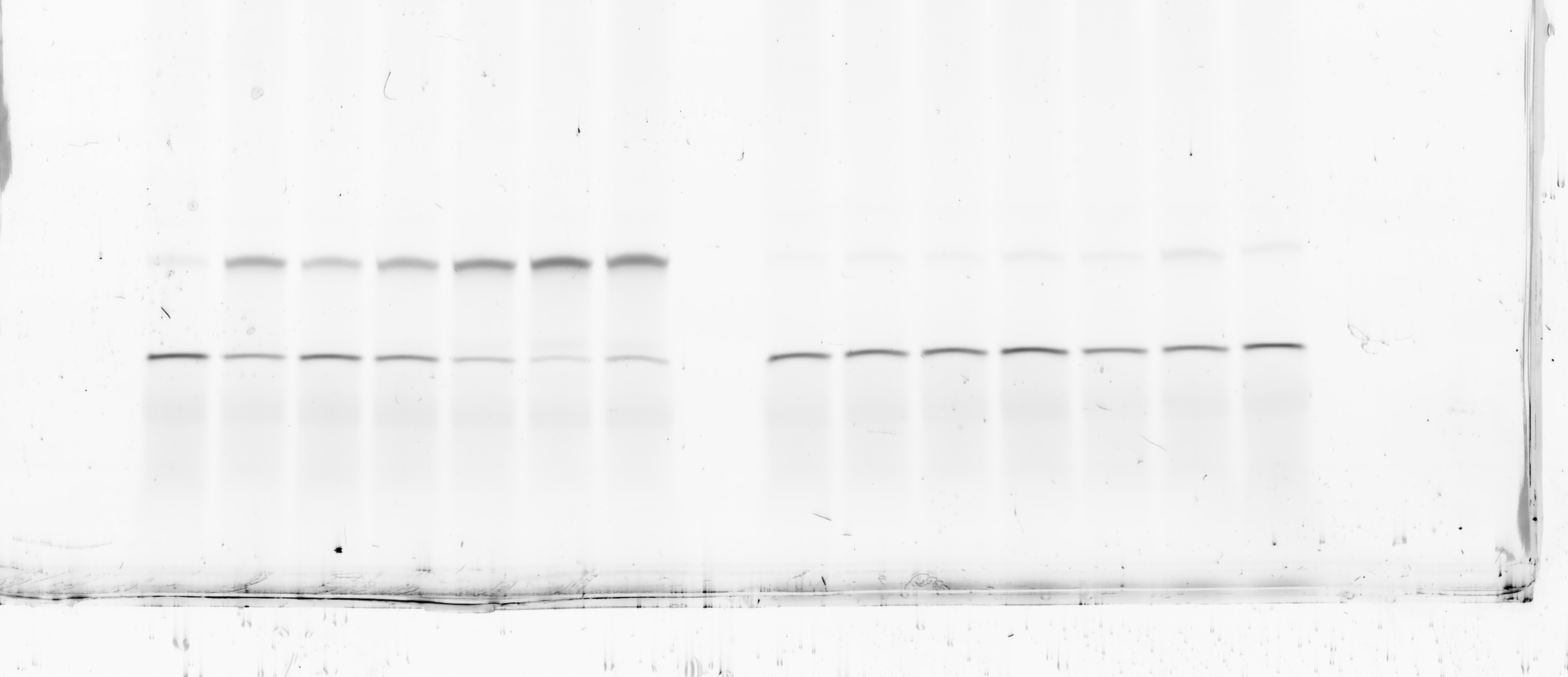

Supplement: Figure 8—figure supplement 2—source data 1. [file elife-83893-fig8-figsupp2-data1.zip › Figure 8-figure supplement 2-source data 1/Figure 8-figure supplement 2-source data 1-raw files/Figure 8-figure supplement 2-source data 1-deaminase assay gel.tif]

Figure 8—figure supplement 3, panel A

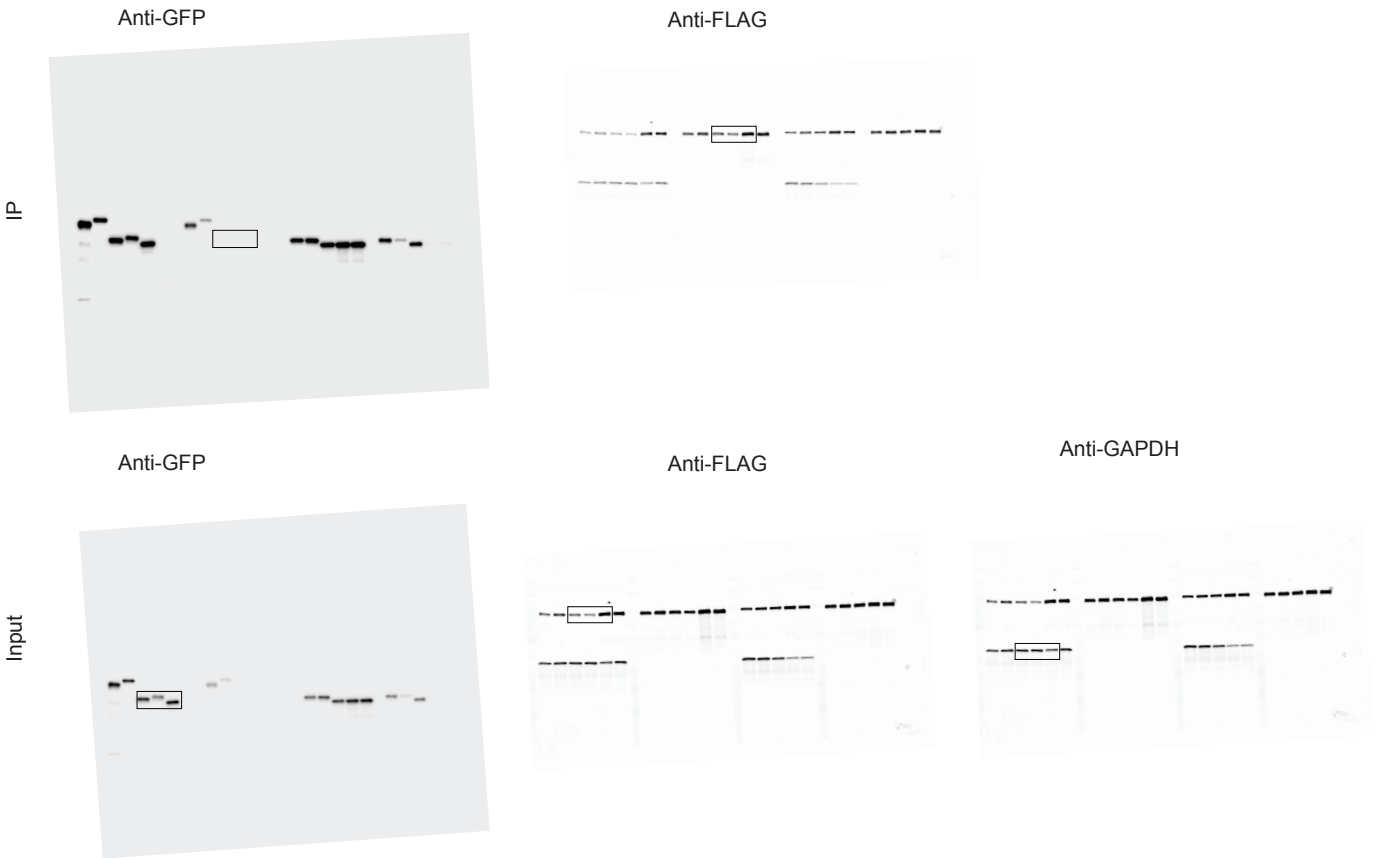

Supplement: Figure 8—figure supplement 3—source data 1. [file elife-83893-fig8-figsupp3-data1.zip › Figure 8-figure supplement 3-source data 1/Figure 8-figure supplement 3-source data 1-uncropped.pdf]

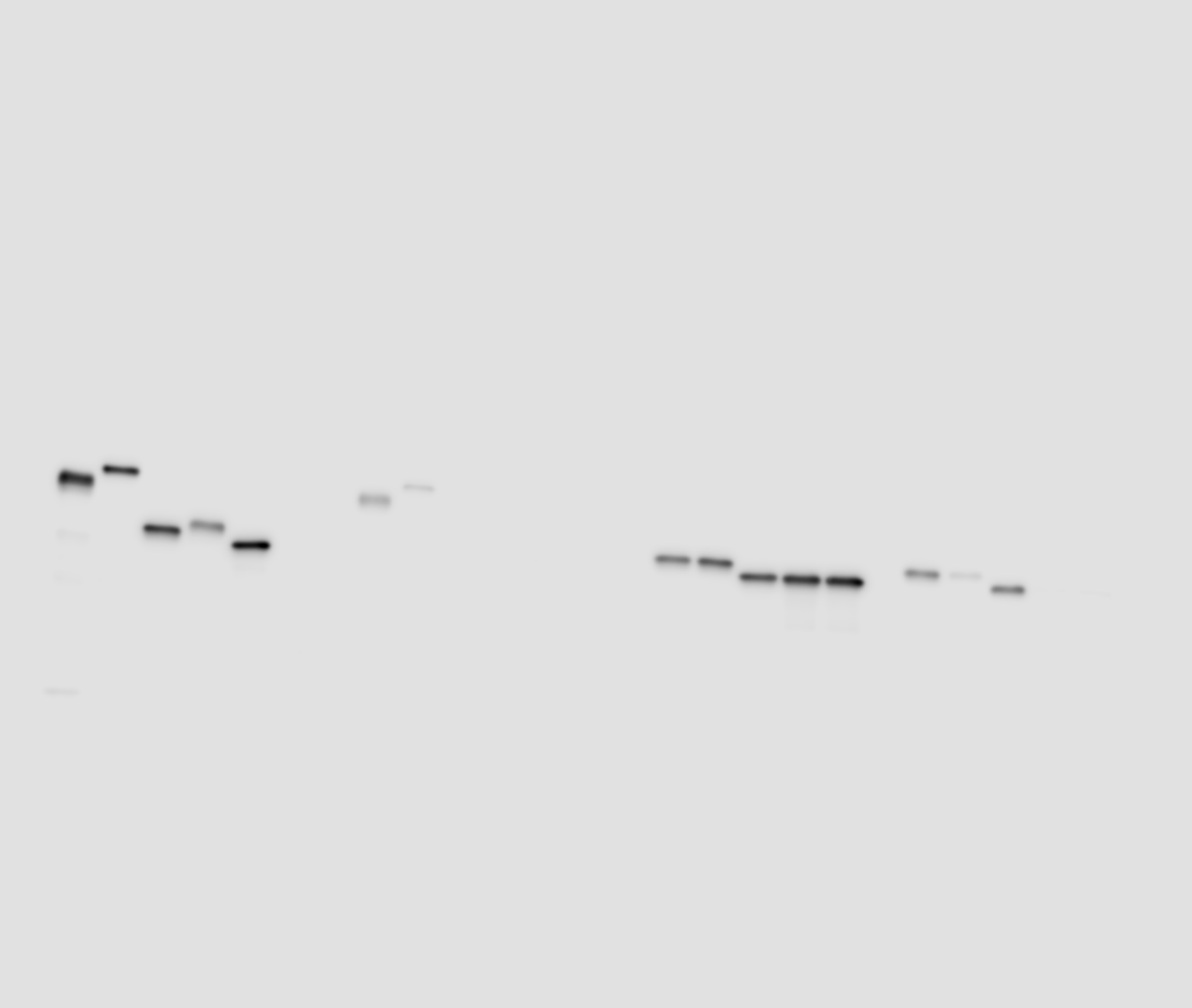

Supplement: Figure 8—figure supplement 3—source data 1. [file elife-83893-fig8-figsupp3-data1.zip › Figure 8-figure supplement 3-source data 1/Figure 8-figure supplement 3-source data 1-raw files/Figure 8-figure supplement 3-source data 1-GFP channel.tif]
